# Design of Promising Uranyl(VI) Complexes Thin Films with Potential Applications in Molecular Electronics

**DOI:** 10.1002/open.202300219

**Published:** 2024-01-05

**Authors:** César Raúl Monzón González, María Elena Sánchez Vergara, Milton Carlos Elías‐Espinosa, Sergio Arturo Rodríguez‐Valencia, Byron José López‐Mayorga, José León Castillo‐Arroyave, Rubén Alfredo Toscano, Octavio Lozada Flores, Cecilio Álvarez Toledano

**Affiliations:** ^1^ Instituto de Química Universidad Nacional Autónoma de México Circuito Exterior s/n. C.U. Delegación Coyoacán, C.P. 04510 Ciudad de México México; ^2^ Facultad de Ingeniería Universidad Anáhuac México Avenida Universidad Anáhuac 46, Col. Lomas Anáhuac Huixquilucan Estado de México 52786 México; ^3^ Tecnológico de Monterrey Escuela de Ingeniería y Ciencias Av. Carlos Lazo 100 Santa Fe, La Loma Ciudad de México México 01389; ^4^ Tecnológico de Monterrey Escuela de Ingeniería y Ciencias Calle del Puente Ejidos de Huipulco, Tlalpan Ciudad de México México 14380; ^5^ Tecnológico de Monterrey Escuela de Ingeniería y Ciencias Carr. Lago de Guadalupe Km. 3.5, Col. Margarita Maza de Juárez Atizapán de Zaragoza Estado de México México 52926; ^6^ Escuela de Química Facultad de Ciencias Químicas y Farmacia Universidad de San Carlos de Guatemala 11 avenida Ciudad de Guatemala Guatemala 01012; ^7^ Facultad de Ingeniería Universidad Panamericana Augusto Rodin 498 Ciudad de México 03920 México

**Keywords:** Electrical Properties, Optical Properties, Thin-Film, Uranyl Complexes, Young's Modulus

## Abstract

In this work, it is proposed the development of organic semiconductors (OS) based on uranyl(VI) complexes. The above by means of the synthesis and the characterization of the complexes by Infrared spectroscopy, Nuclear magnetic resonance spectroscopy, mass spectrometry, and X‐ray diffraction. Films of these complexes were deposited and subsequently, topographic and structural characterization was carried out by Scanning Electron Microscopy, X‐ray diffraction, and Atomic Force Microscopy. Additionally, the nanomechanical evaluation was performed to know the stiffness of uranyl films using their modulus of elasticity. Also, the optical characterization took place in the devices and their bandgap value ranges between 2.40 and 2.93 eV being the minor for the film of the uranyl complex with the N on pyridine in position 4 (**2 c**). Finally, the electrical behavior of the uranyl(VI) films was evaluated, and important differences were obtained: the uranyl complex with the N on pyridine in position 2 (**2 a**) film is not influenced by changes in lighting and its current density is in the order of 10^−3^ A/cm^2^. The film with uranyl complex with the N on pyridine in position 3 (**2 b**) and **2 c** presents a greater current flow under lighting conditions and two orders of magnitude larger than in film **2 a**. In these films **2 b** and **2 c**, ohmic behavior occurs at low voltages, while at high voltages the charge transport changes to space‐charge limited current behavior.

## Graphical abstract

Oxo uranyl(VI) complexes were successfully synthesized, characterized and deposited by thermal evaporation and optical and electrical features of films were examined. The bandgap for the films is in the Interval of 2.39 and 2.79 eV and the films present ohmic electrical behavior, with maximum current values of order of 10^−3^ A. These uranyl films can be used in Molecular Electronics applications.

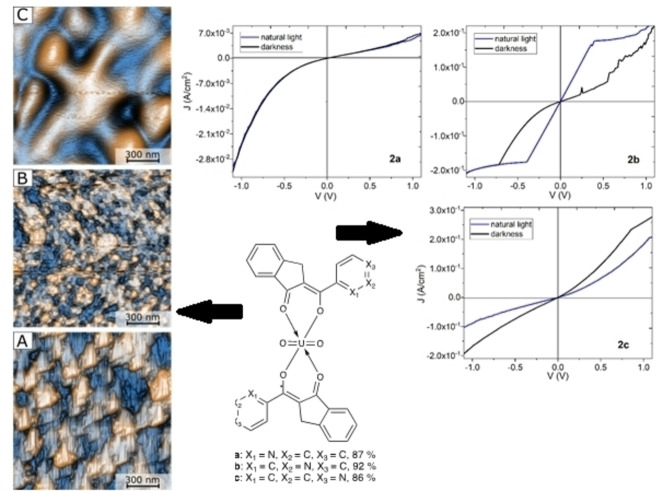

## Introduction

Organic semiconductors (OS) have become an option to be used in the manufacture of optoelectronic devices. According to Catania et al.,^[1]^ the possibility of depositing organic semiconductor films on a variety of substrate materials is essential for the ongoing trend to fabricate electronic devices on substrates with tailored properties. One of these properties is mechanical flexibility, which has become attractive because of the possibility of having stable semiconductor films on non‐rigid surfaces. These conformable electronics are achieved by ultra‐thin lightweight and transparent polymeric substrates.[^2^, ^3^, ^4^, ^5^] Unachievable for rigid electronics, the mechanical properties of these systems are a key factor: Young's modulus, ductility, resilience, rigidity, and strength, provide indications on which applications could be tackled.^[1]^ In addition to the polymeric substrates and their characteristics mentioned above, semiconductor films comprise a fundamental part of the building of electronic devices.^[1]^ Thanks to the nanomechanical properties in the OS, it is possible to create flexible films with high resistance to fracture that don't lose efficiency electronically. The application of surface technologies focused on the manufacture of semiconductor films has had a great impact on studies of mechanics, as well as in electronic issues, which have directly influenced, the miniaturization of electronic components.[^6^, ^7^, ^8^, ^9^, ^10^, ^11^, ^12^, ^13^, ^14^, ^15^, ^16^, ^17^] Multiple techniques allow the manufacture of OS films, deposition by vacuum thermal deposition is one of the most used in the manufacture of optoelectronic devices.[^18^, ^19^, ^20^, ^21^, ^22^, ^23^] During thermal deposition, the OS is brought to a vapor phase by heating it at low pressures (10^−6^–10^−8^ torr).^[24]^ As the vapor reaches the substrate, thin film formation proceeds through the nucleation and growth of molecules of the deposited semiconductor.^[24]^ The formation of films is a process that can be influenced by many factors, such as semiconductor properties, deposition parameters, and substrates used. These factors result in film microstructure ranging from the formation of single crystals, to polycrystalline structures, and amorphous films.^[24]^ From the above, it follows that it is necessary to develop organic semiconductors that can be deposited as thin films on substrates of different types.

**Figure 1:**
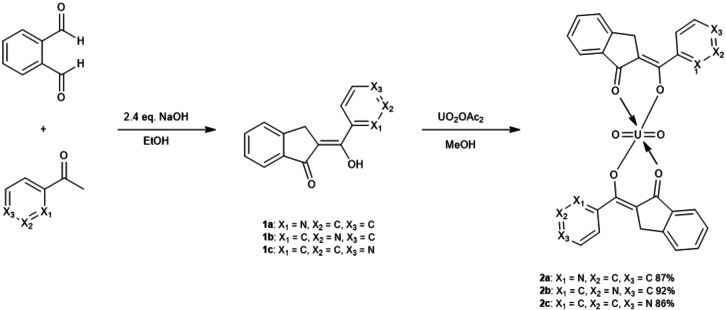
Synthesis of uranyl(VI) complexes.

An original option is presented in this work, in which the synthesis, characterization, and deposition on flexible and rigid substrates of new semiconductors based on uranyl(VI) complexes with different indanones is proposed. Some research groups have undertaken the task of investigating uranium complexes to evaluate their optical properties and/or applications in sensors,^[25]^ which can also modulate their use in photophysical devices, due to their oxidizing properties in the presence of certain wavelengths of light that allow their use as a catalyst,[^26^, ^27^, ^28^, ^29^, ^30^] addition reaction, C−H activation, organic degradation, ring‐opening polymerization, etc..[^31^, ^32^, ^33^] Besides, the use of organic ligands has been used for the investigation of new uranyl complexes, there are numerous reports of uranyl complexes with carboxylates,^[34]^ neutral ligands such as Schiff bases,^[35]^ and diketones,^[36]^ among others. Our research group has developed a new method for the synthesis of hydroxymethylidene indanones that have an enolizable structure and a behavior similar to that of diketones. The indanones have shown bioactive properties.^[37]^ They have also been used as ligands for the synthesis of different coordination complexes of Fe, Cu, B, Sn, and V with which thin films showing semiconducting properties were prepared.[^38^, ^39^, ^40^, ^41^, ^42^] One of the most common forms of uranium is UO_2_^2+^ which forms very stable complexes, oxo uranium complexes usually have a coordination number of 6 or 7 and can have a range of oxidation states from +3 to +6. These types of compounds are photochemically excitable under the ultraviolet and blue light spectrum, so some research has focused on the use of these materials as molecular components capable of promoting the oxidation of organic substrates.^[30]^ However, there are practically no studies on thin films of these types of compounds and their application as OS in optoelectronic devices. The film growth of OS of the uranyl complexes is far from being understood, and it is for the above, that in this work we propose the synthesis, characterization, and deposit of new uranyl(VI) complexes. We used three indanones with pyridinic fragments **1 a** and **1 c** that we have previously described^[37]^ and a new hydroxymethylidene indanone (**1 b**) that was fully characterized (see supplementary information). The uranyl(VI) complexes (**2 a**, **2 b**, and **2 c**) synthesized from these precursors, were subsequently deposited as thin films on different kinds of substrates, using the vacuum thermal deposition technique. The films deposited were morphologically and nanomechanically characterized, to finally, evaluate and compare their optical and electrical behavior and determine their possible application to organic electronic. The uranyl(VI) complexes analyzed in this study are those shown in Figure 1, it can be observed that the difference between the complexes is the position of the nitrogen in the pyridine of the hydroxymethylidenindanone.

## Materials and Methods

### General Information

All reagents and solvents were obtained from commercial suppliers (Sigma‐Aldrich) and used without further purification. Melting points were obtained on a Melt‐Temp II apparatus. The compounds were characterized by FTIR, dissolved in spectroscopic grade KBr (Sigma‐Aldrich), and recorded on a Bruker Tensor 27 spectrometer. The FTIR in films deposited on silicon substrate was carried out using a Nicolet iS5‐FT spectrometer. The ^1^H and ^13^C NMR spectra of the compounds in the DMSO solution were recorded on a Bruker 500 Ascend spectrometer at 700.0 and 175.0 MHz, respectively. Mass spectra were recorded with a JEOL‐JMS‐X103 Spectrometer, by the fast atom bombardment (FAB^+^) technique and using a matrix of poly(ethylene glycol) 600, and with High‐Resolution PI Mass Spectrometer (SQ‐MS‐GC) Agilent Tech. A suitable X‐ray quality crystal of one uranyl complex (**2 b**) was grown by slow evaporation of a dimethyl sulfoxide at room temperature. The UV‐vis spectra of uranyl(VI) complexes in methanol solution were obtained on a Genesys 10S UV‐Vis spectrophotometer. The study of the thermal degradation was carried out using a LECO Cornerstone thermogravimetric analyzer TGA801. In each trial, a 0.02 g constant mass sample was placed in a 10 μL ceramic crucible, then heated from room temperature to 750 °C, using a nitrogen atmosphere and a total flow rate of 10 mL min^−1^. Additionally, the uranyl complexes in powders and the films were examined by X‐ray diffraction analysis (XRD) using the θ–2θ technique in a Rigaku Miniflex 600 diffractometer with Cu Kα (λ=1.5406 Å), at 40 kV, 20 mA. For the morphological characterization of the film, a Hitachi Tabletop Microscope TM3030 was used at 5 kV. EDS and elemental mapping were carried out in a JEOL JCM‐6000 NeoScope Benchtop Scanning Electron Microscope at 15 kV. Atomic Force Microscopy (AFM) for the films on quartz and indium tin oxide coated polyethylene terephthalate (PET‐ITO) substrate was performed on a *SU3500* microscope (Hitachi, Ltd.) and an XE7 microscope (Park Systems) located inside an acoustic insulation chamber. The optical properties of the deposited samples were obtained in the 200–1100 nm wavelength range using a UV‐Vis 300 Unicam spectrophotometer equipped with an integrating sphere. For the evaluation of electrical properties, the four‐point collinear method was used, with a programmable voltage source, a sensing station with a lighting controller circuit from Next Robotix, and an auto‐ranging Keithley 4200‐SCS‐PK1 pico‐ammeter was employed.

Deposition Number 2260655 (for **2 b**) contains the supplementary crystallographic data for this paper. These data are provided free of charge by the joint Cambridge Crystallographic Data Centre and Fachinformationszentrum Karlsruhe Access Structures service.

### Synthesis and Characterization

*Synthesis of hydroxymethylidene indanones* (**1 a, 1 c**). According to Figure 1 and previous reports by some of the authors of this work,^[37]^ *o*‐phthalaldehyde was added to an ethanolic solution of sodium hydroxide (1.5 eq.) with 1 equivalent of the corresponding acetophenone. The reaction mixture was stirred at 0 °C for 3 hours and then poured into a mixture of ice and commercial hydrochloric acid (until pH 3 was reached). The resulting solid was filtered and purified by column chromatography using hexane/ethyl acetate as eluent. The new **1 b** indanone was synthesized using the same methodology starting from 1‐(pyridin‐3‐yl)ethan‐1‐one and was fully characterized showing the expected signals for this kind of compound (see support information).

*Synthesis of uranyl(VI) complexes (**2 a**–**c**)*. According to Figure 1, a solution of the hydroxymethylidene indanone derivate (2 eq.) and uranyl acetate (1 eq.) in MeOH was heated under reflux for 1 hour. The precipitate formed is filtered and washed with methanol and diethyl ether.

**Ligand 1 b**: C_15_H_11_NO_2_, M_r_=237.26 g mol^−1^ **1 b** was prepared starting from *o*‐phtalaldehyde (1.00 g, 7.47 mmol) and 1‐(pyridin‐3‐yl)ethan‐1‐one (0.85 mL, 7.77 mmol) and was obtained as a yellow solid., m.p.=128 °C. Yield: 1.7710 G, 7.46 mmol (66 %). **IR**: *ν*=1608, 1556, 1471, 738 cm^−1^. ^**1**^ **H NMR**: (300 MHz, Chloroform‐*d*): δ=3.76 (2H, s, H‐16), 7.20‐7.49 (4H, m, H‐13, H‐12, H‐14, H‐4), 7.71 (1H, d, J=4.9, 1.4 Hz, H‐11), 8.06 (1H, dt, J=8.1, 1.9 Hz, H‐13) 8.56 (1H, dd, J=4.9 Hz, H‐16), 8.99 (1H, d, J=1.8 Hz, H‐12. ^**13**^ **C NMR**: (75 MHz, Chloroform‐*d*): 31.9 (C‐16), 110.2 (C‐8), 123.6 (C‐11, C‐12), 125.8 (C‐14), 127.7, (C‐4), 130.9 (C‐6), 133.8 (C‐13), 135.3 (C‐5), 137.6 (C‐10), 148.5 (C‐15), 149.1 (C‐1), 151.7 (C‐3), 167.7 (C‐7), 196.1 (C‐9). **MS (DART^+^)**: *m/z* (%): 238 (100), [M+1]^+^. **MS (ESI)**: *m/z* (%): 238.08529 (100) [M^+^+1].

**Complex 2 a**: C_30_H_20_N_2_O_6_U, M_r_=742.18 g mol^−1^ **2 a** was prepared starting from **1 a** (0.1 g, 0.42 mmol) and uranyl acetate (0.1073 g, 0.51 mmol) and was obtained as a red solid., m.p. >250 °C. **IR**: *ν*=1593, 1575, 1556, 1451, 912, 731, 248 cm^−1^. ^**1**^ **H NMR**: (700 MHz, DMSO‐*d_6_*): δ=3.32 (2H, s, H‐16), 7.66 (1H, m, H‐12), 7.71 (1H, m, H‐13), 7.74 (1H, t, J=3 Hz, H‐4) 7.80 (1H, d, J=3 Hz, H‐5), 8.28 (1H, s, H‐11), 8.91 (1H, s, H‐2). ^**13**^ **C NMR**: (175 MHz, DMSO‐*d_6_*): δ=34.8 (C‐16), 114.3 (C‐8), 123.4 (C‐11), 124.9 (C‐8), 126.2 (C‐10), 126.5 (C‐3), 127.9 (C‐12), 133.2 (C‐4), 140.1 (C‐6), 149.7 (C‐2), 150.2 (C‐2), 157.9 (C‐15), 179.5 (C‐7), 193.5 (C‐9). **MS (FAB^+^)**: *m/z* (%): 743 (1) [C_30_H_21_N_2_O_6_U]^+^. **MS (ESI)**: *m/z* (%): 821.2144 (15) [M^+^+DMSO], 662.1478 (100) [M^+^−237 (indanone)+2 DMSO], 584.1331 (19) [M^+^−237 (indanone)+DMSO].

**Complex 2 b**: C_30_H_20_N_2_O_6_U, M_r_=742.18 g mol^−1^ **2 b** was prepared starting from **1 b** (0.1 g, 0.42 mmol) and uranyl acetate (0.1073 g, 0.51 mmol) and was obtained as a red solid., m.p. >250 °C. **IR**: *ν*=1594, 1564, 1469, 1453, 988, 737, 264 cm^−1^. ^**1**^ **H NMR**: (700 MHz, DMSO‐*d_6_*): δ=3.33 (2H, s, H‐16), 7.67 (1H, m, H‐14), 7.74–7.76 (2H, m, H4, H12), 8.26 (1H, m, H‐13), 8.73 (H, s, H‐5), 8.87 (H, H‐3). ^**13**^ **C NMR**: (175 MHz, DMSO‐*d_6_*): δ=33.4 (C‐16), 114.2 (C‐8), 123.5 (C‐13), 124.2 (C‐3), 126.5 (C‐12), 128.1 (C‐14), 133.2 (C‐4), 135.8 (C‐6), 136.9 (C‐5), 140.1 (C‐10), 149.1 (C‐15), 150.1 (C‐1), 152.1 (C‐3), 181.5 (C‐7), 191.3 (C‐9). **MS (FAB^+^)**: *m/z* (%): 743 (1) [C_30_H_21_N_2_O_6_U]^+^. **MS (ESI)**: *m/z* (%): 662.1493 (100) [M^+^−237 (indanone)+2 DMSO], 584.1349 (19) [M^+^−237 (indanone)+DMSO].

**Complex 2 c**: C_30_H_20_N_2_O_6_U, M_r_=742.18 g mol^−1^ **2 c** was prepared starting from **1 c** (0.1 g, 0.42 mmol) and uranyl acetate (0.1073 g, 0.51 mmol) and was obtained as a red solid., m.p. >250 °C. **IR**: *ν*=1592, 1574, 1450, 902, 733, 264 cm^−1^. ^**1**^ **H NMR**: (700 MHz, DMSO‐*d_6_*): δ=4.1 (2H, s, H‐16), 7.53 (1H, t, H‐12), 7.68 (1H, d, H‐14), 7.72 (1H, d, H‐13), 7.84 (1H, d, H‐11), 7.87 (2H, d, H‐1, H‐5), 8.81 (2H, d, H‐2, H‐4). ^**13**^ **C NMR**: (175 MHz, DMSO‐*d_6_*): δ=33.2 (C‐16), 114.4 (C‐8), 122.1 (C‐1, C‐5), 123.1 (C‐11), 126.7 (C‐14), 128.2 (C‐12), 133.54 (C‐13), 139.8 (C‐10), 149.3 (C‐15), 150.9 (C‐2, C‐4), 167.3 (C‐7), 192.2 (C‐9). **MS (ESI)**: *m/z* (%): 662.1492 (100) [M^+^−237 (indanone)+2 DMSO], 584.1336 (19) [M^+^−237 (indanone)+DMSO].

### Uranyl(VI) films Deposition and Characterization

To obtain the uranyl films from the vacuum deposition technique, monocrystalline n‐type silicon wafers (c‐Si), quartz, glass, and indium tin oxide (In_2_O_3_ ⋅ (SnO_2_)_x_) coated polyethylene terephthalate (PET‐ITO) were used as substrates. The quartz and glass substrates were washed in an ultrasonic bath with dichloromethane, methanol, and acetone. The n‐type silicon substrate was washed with a “p” solution (15 mL HNO_3_, 10 mL HF and 300 mL H_2_O), to remove surface oxide. Thin films were deposited in a high vacuum deposition system using molybdenum crucibles, a vacuum pressure of 1×10^−6^ torr, and a deposit speed between 0.005 and 0.04 Å/s. The uranyl(VI) complexes were heated to 250 °C to produce their phase change, which was initially carried out in the gaseous state so that they would finally be deposited in the form of thin films upon contact with the substrates, which were at room temperature. The thickness was obtained using a microbalance quartz crystal monitor, connected to a thickness sensor and the thicknesses obtained were 350, 240, and 101 nm for **2 a**, **2 b**, and **2 c** films respectively. These films on an n‐type silicon substrate were structurally and morphologically characterized by FTIR spectroscopy, EDS, SEM, and XRD analysis. Subsequently, non‐contact mode in AFM was used to obtain a topographic characterization, and to get the nanomechanical properties, fast force spectroscopy mapping (also known as *Pin Point* mode) was implemented. Each one of these characterization methods needs a different type of probe. For the topographic characterization, a Point Probe Plus Non‐Contact/tapping mode High resonance frequency Reflex coating (PPP‐NCHR, 330 Hz, 42 N/m,) with an aluminum coating was used while for the nanomechanical characterization, a Special Development AFM tip Radius 30 nm standard Force Modulation probe (SD‐R30‐FM, 75 Hz, 2.8 N/m) with no coating was required. The optical behavior and the bandgap of each film were obtained by UV‐vis spectroscopy and finally, current‐voltage (I–V) measurements were performed on the uranyl(VI) films. The electrical characterization of the films was carried out using the four‐point collinear method. In this type of arrangement, four tips of equal spacing (1.59 mm) and in line are placed on the film. The measurements were carried out in natural lighting conditions and in dark conditions.

## Results and Discussion

### Synthesis and characterization of the complexes of uranyl(VI)

**Figure 2:**
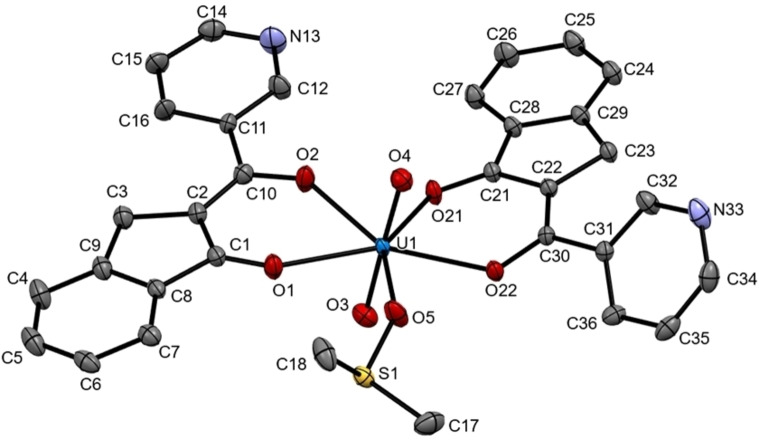
ORTEP plot of complex **2 b** was drawn at the 50 % probability level.

Firstly, the precursors were synthesized according to our reported procedure as shown in Figure 1. The *o*‐phthalaldehyde and the corresponding aryl‐substituted methyl ketone were mixed with an ethanolic solution forming the compounds in good yields. Afterward, the uranyl(VI) complexes **2 a**–**2 c** were synthetized from a 1 : 2 ratio of uranyl(VI) acetate, and the corresponding indanone in methanol. The complexes were pure isolated as red solids in yields ranging from 85 to 92 %. All the new complexes have been characterized by means of conventional spectroscopic techniques. The infrared spectra of all complexes (see SI and Table 1) show an absorption band in 1545–1595 cm^−1^ region that was assigned to *v*(C=O) and *v*(C=C) stretching vibrations with considerable mixing.[^37^, ^38^, ^39^, ^40^, ^41^, ^42^] The presence of this band in the spectra of the complexes, which moves to lower frequencies in comparison with the ligand, supports the consideration that oxygen is coordinated to the uranium atom in complexes **2 a**–**2 c**. For the coordinated U=O, appears one intensive band around 900 cm^−1^. Stretching bands that occur around 730 and 250 cm^−1^ were attributed to *v*(O−U−O).[^37^, ^38^, ^39^, ^40^, ^41^, ^42^] The molecular mass of complexes **2 a**–**2 c** was established by mass spectrometry by the FAB^+^ technique and the spectra showed the [M]^+^ ion molecular corresponding to the complex. The molecular mass of complexes **2 a**–**2 c** were established by mass spectrometry by the ESI technique that the spectra showed the [M+DMSO]^+^ ion corresponding to the complex plus DMSO. It is important to mention that in some spectroscopic techniques, DMSO appears coordinated to the complex, this is because the NMR was first performed in DMSO solution, at that time it was coordinated, increasing the coordination sphere of the complex, subsequently, and other techniques were performed. Besides, the structural arrangement for **2 b** was unequivocally established by single‐crystal X‐ray diffraction analysis. A single crystal was obtained from a DMSO solution (Figure 2). The structure crystallized in a P‐1 triclinic space group, shows a bipyramidal pentagonal coordination sphere with two indanones and one DMSO in equatorial positions and two oxygens in axial positions, a notorious bond distance is also observed between U1‐O3 and U1‐O4 being the shortest bonds that are attached to the uranium atom which would correspond to the oxygens attached to U1 by double bond, the rest of the distances between U1 and oxygen range between 2.315–2.450 Å, which indicates that they are distances greater than those of a common covalent bond, these being coordination bonds with U1. The bond distances between C1‐C2, C2‐C10, C30‐C22, and C22‐C21 are very similar, which indicates homogeneous electronic delocalization between C1‐C2‐C10 and C30‐C22‐C21. The angles between U1 show a distorted pentagonal structure where O1‐U1‐O2 and O22‐U1‐O21 show slightly steeper angles corresponding to the dicarbonyl clamp, and O5‐U1‐O22 shows the largest angle at 75.68(5)°, which corresponds to oxygen from an indanone and oxygen from DMSO. The angles between the axial positions O3‐U1‐O4 present 178.36(6)° a slight distortion.

**Table 1:** Comparison between the IR signals obtained for the uranyl(VI) complexes in KBr pellet and thin film.

Assignment	**2 a** Pellet (cm^−1^)	**2 a** as‐deposited films (cm^−1^)	**2 b** Pellet (cm^−1^)	**2 b** as‐deposited films (cm^−1^)	**2 c** Pellet (cm^−1^)	**2 c** as‐deposited films (cm^−1^)
v (C−H aromatic)	3048	3051	3028	3028	3071	3073
v (C−H)	2905	2901	2899	2901	2897	2896
v (C=O)	1593	1594	1594	1598	1592	1596
v (C=C enol)	1556	1559	1565	1567	1574	1574
v (U=O)	912	921	898	898	902	906

**Figure 3:**
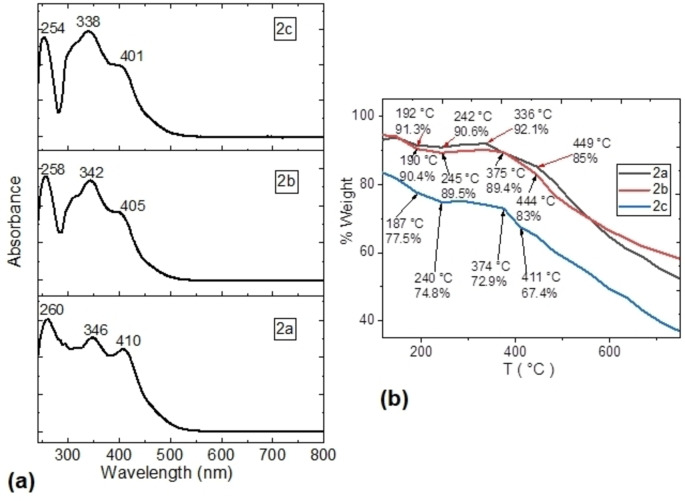
(a) Spectra UV‐vis in methanol and (b) TGA curves of the thermal decomposition for the complexes uranyl(VI).

UV‐vis spectroscopy was performed to determine the optical behavior of uranyl(VI) complexes in solution. Figure 3a shows the UV‐vis absorption spectra of these complexes in MeOH with vibronic structures relatively well resolved, due to the rigid structure of the complexes. The spectrums are shown the wavelengths corresponding to the different vibronic peaks of each optical absorption. In all the cases there is intense absorption in the range of 254 to 410 nm, with maximum absorption of lower energy at 257±3 nm, a second and intense band at 342±4 nm, and finally, a band at higher energy at 405±5 nm. Considering the spectrum of compound **2 c**, the optical absorptions shift to lower energies in the spectrum of compound **2 b**, being the lowest, those that are in the UV‐vis spectrum of compound **2 a**. Apparently, the position of the nitrogen atom in the radical pyridine is a determinant factor in the optical behavior of these complexes. The absorbance of these compounds is an indication that they can be used in optoelectronics. However, it is necessary to deposit them as a thin film to study their behavior in the solid state. To manufacture the thin films, it was necessary to assess the thermal stability of the uranyl complexes using Thermogravimetric Analysis (TGA) under a nitrogen atmosphere. Figure 3b shows the thermal profile obtained from TGA curves for uranyl(VI) complexes, collected at a heating rate of 6 °C⋅min^−1^, spanning a temperature range from 25 to 750 °C. The degradation profile of complexes unfolded in four stages within a temperature range of 118 to 449 °C, with maximum weight loss rates occurring at T_max_ 449, 444, and 411 °C for **2 a**, **2 b**, and **2 c**, respectively. The order of thermal stability was **2 b**>**2 a**>**2 c**. The uranyl(VI) complexes exhibited adequate thermal stability, enabling their deposition as films using the vacuum thermal evaporation technique. It was interesting how complexes **2 a** and **2 b** exhibited similar temperatures and decomposition percentages, as expected given their closely related chemical structures. However, Complex **2 c** showed significant variations, particularly in the percentage of decomposition, which may be attributed to the nitrogen in the 4‐position. Further examination of these compounds in the solid state will determine their potential for use in organic electronics.

### Deposition and characterization of uranyl(VI) films

**Figure 4:**
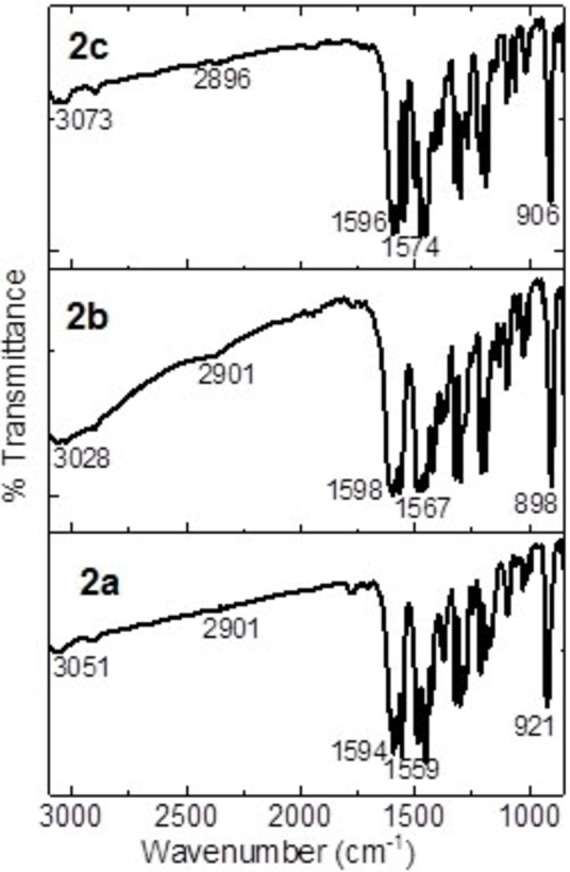
Spectra IR in film, for the complexes uranyl(VI).

The interest in the complexes of uranyl(VI) in solid state is based on the fact that besides being photochemically excitable,^[30]^ they show a rigid structure, with aromatic rings with high π‐conjugation and the possibility to modify the pyridine nitrogen in their structure, which can lead to better mobility of charge carriers. For the manufacture of thin films by the vacuum deposition technique, it is important to consider that, during evaporation, the uranyl compounds may raise temperatures around 250 °C and when deposited on a substrate which is at room temperature, it generates a high thermal gradient. This gradient may cause the chemical decomposition of the uranium compounds, or subsequent physical and mechanical deterioration of thin films. To review the above, IR spectroscopy was carried out, looking for the presence of the main functional groups of the uranyl(VI) complexes in the thin films. In Figure 4 and Table 1, are the correspondent assignments to: (i) the C−H bonds, (ii) the bond C=O, (iii) the assignment for C=C, and (iv) the bond U=O.[^37^, ^38^, ^39^, ^40^, ^41^, ^42^] From the IR spectroscopy results, it is concluded that the components in the thin films did not suffer degradation during the deposit. These results are important because very little has been studied about the fabrication of thin films of uranium complexes by vacuum deposition technique. One of the most important requirements of this technique is the thermal stability of the complexes to be deposited, since on the one hand; uranium complexes must not chemically decompose during their deposition, but on the other hand; and under service conditions, optoelectronic devices get hot, the low thermal stability of these complexes would decrease the functionality and efficiency of the device. The IR spectroscopy results indicate that apparently the three compounds are stable and can be deposited by the high vacuum evaporation technique, however, it is necessary to complete the information using SEM and EDS.

**Figure 5:**
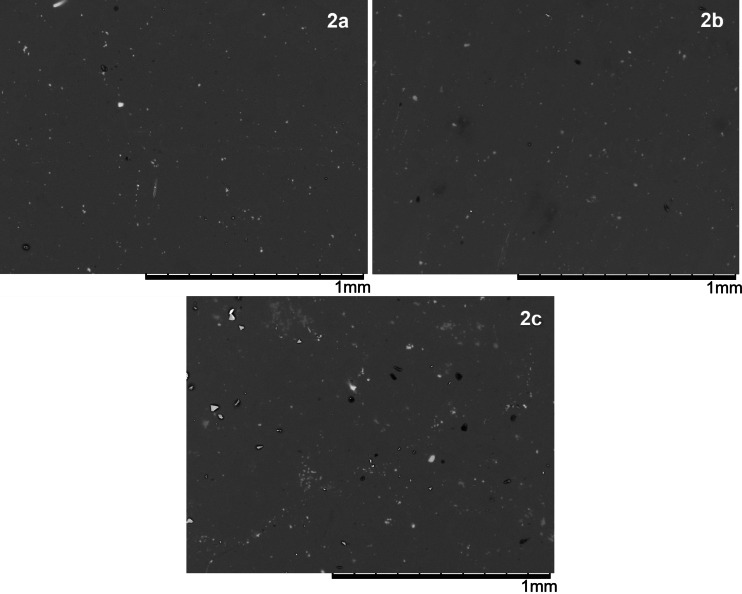
SEM at 100× in film, for the complexes uranyl(VI).

When analyzing the morphology of the films using SEM (see Figure 5), similarities are observed in the three cases. The films are heterogeneous, formed by a base of the uranyl complex, with irregularly shaped particles of different sizes, distributed along their entire surface. It is important to consider; that film **2 c** is the one that contains the largest particles, which are formed during its nucleation and growth process. In the vacuum thermal deposition process of uranyl(VI) complexes, they initially change from a solid state to a gaseous state. Subsequently, the complex comes into contact with the substrate that is at room temperature, generating a new phase change, from a gaseous state to a solid state, which completely covers the substrate in the form of a film. The last material to be deposited nucleates and grows on this uranyl film in preferential directions, which gives rise to particles of different shapes and sizes, observed in the SEM images for the three films.

**Figure 6:**
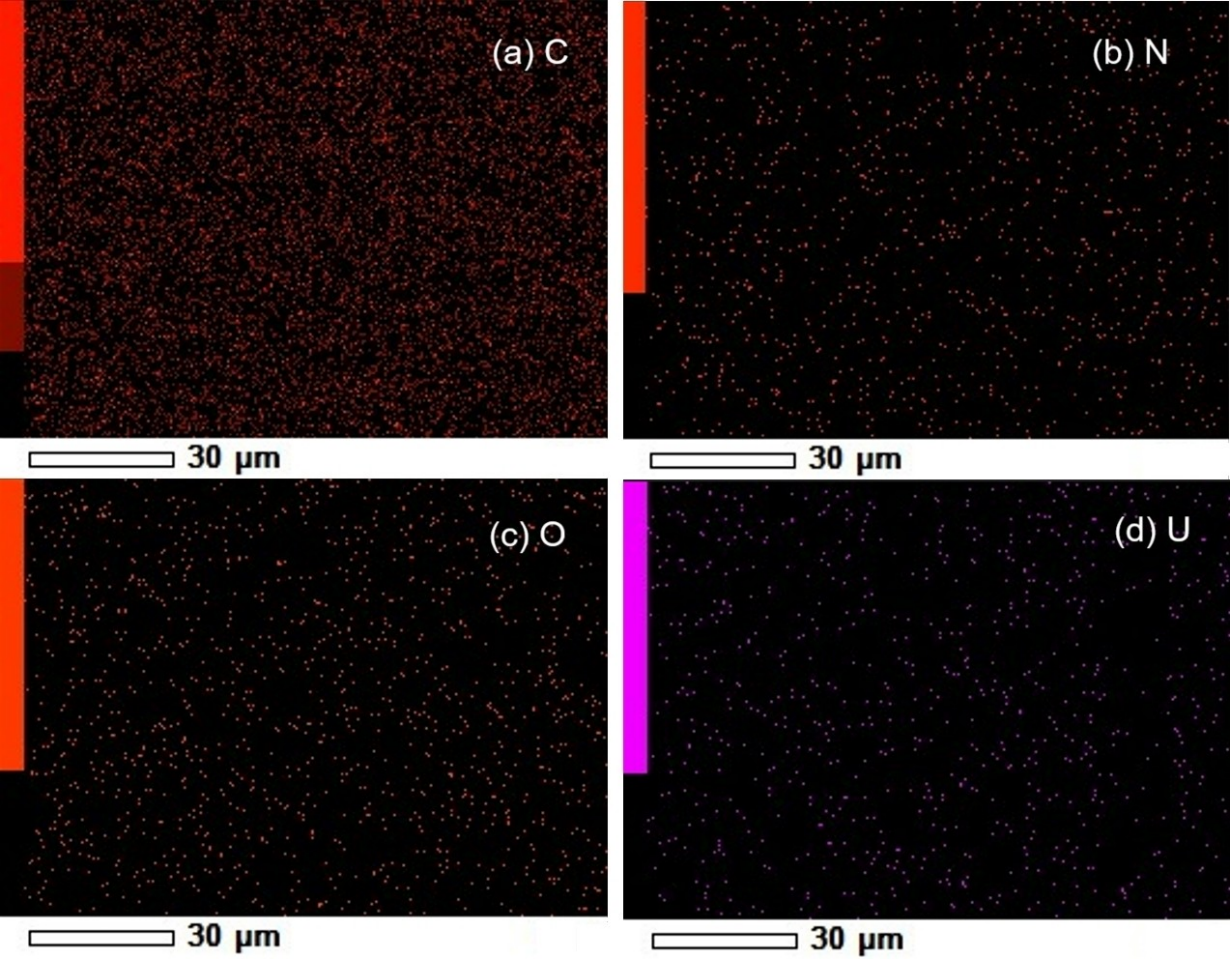
EDS for the uranyl(VI) complex **2 c** in film.

Additionally, the EDS results for complex **2 c** (see Figure 6), indicate that the film is composed of oxygen, nitrogen, carbon, and uranium. Furthermore, it is observed that these elements are distributed heterogeneously within the film. According to Figure 1, carbon is the most abundant element, with uranium being the least abundant, which is consistent with the chemical formula of the complexes uranyl(VI). These results are similar for the films of the other two uranium complexes.

**Figure 7:**
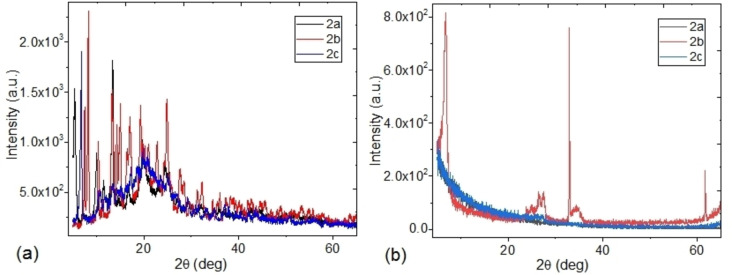
DRX for (a) powders and (b) films for the uranyl(VI) complexes.

As a complement to the morphological analysis, the structural study was carried out on the uranium complexes. Figure 7a shows the diffraction patterns for these powder complexes. The highest crystallinity occurs in complex **2 b**, followed by complex **2 a**, while complex **2 c** presents a mainly amorphous structure. When comparing these results with those obtained in the XRD study of the films, a radical change in the structure of the uranyl(VI) complexes is observed in Figure 7b. The films **2 a** and **2 c** present a diffraction pattern related to an amorphous structure. No sharp Bragg peaks that could be related to a crystalline structure were presented. This indicates that the particles on the surface of the films and the film itself are essentially amorphous. However, the XRD pattern of **2 b** film exhibits diffused diffraction reflections at 2θ=6.7°, in the region of 24–27°, in the region of 32–35° and 2θ=62°. These results are an indication of the crystalline structure of the film however, when comparing these results with its powder diffraction pattern; it is evident that the film structure becomes amorphous, because of the deposit technique. Furthermore, it is necessary to consider that the change in the diffraction patterns for the different complexes is related to the change in the position of the nitrogen in the pyridine.

**Figure 8:**
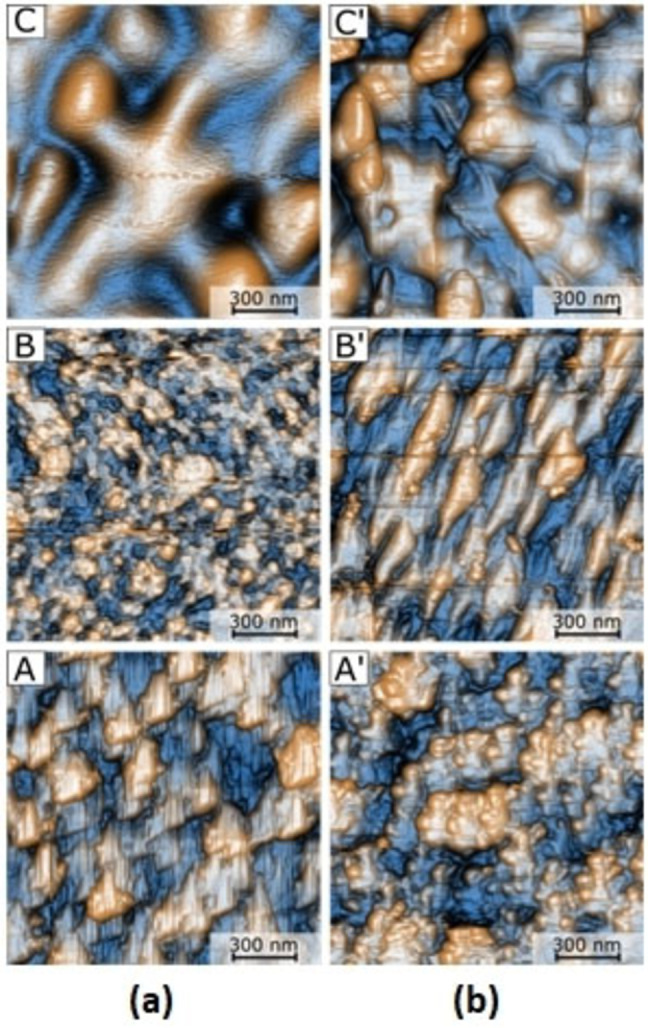
Surface topography of the uranyl complexes over (a) quartz and (b) PET substrates.

During the deposition, the change of surface energy in the substrate can affect the film growth behavior and influence the device performance of semiconductor films.^[43]^ Therefore, knowing the topography is essential for the study of charge transport in these uranyl(VI) films, and their mechanical evaluation will help to establish the type of device that the films can be part of, and their resistance under service conditions. The efficient transport of charges through the OS requires the thin films to be continuous and ordered. The uranyl films deposited by vacuum thermal deposition form an amorphous structure that can give place to low‐charge mobility. Because of the above, it is important to analyze topography, since the increase in the size of the grains and rugosity, along with a good continuity in the films, is a good approach to improve the transport and the mobility of charges. Another important aspect to study is whether uranyl films have an adequate morphology when they are on polymeric substrates such as PET. It is for this reason that the morphology was studied by AFM in thin films deposited on PET, but also on a rigid substrate such as quartz.[^44^, ^45^] Figure 8 shows the image for a 1.5×1.5 μm area from each one of the uranyl (VI) complexes. Each one of these complexes generated a different morphology which also varies from one substrate to another. Complex **2 a** deposited on quartz (Figure 8a) generated a dispersed particle topography with peaks between 25 and 65 nm in height in a sharp triangular geometry of approximately 150 nm in length. This same complex, over a PET substrate (Figure 8b), generated a distribution, height, and particle sizes similar to those generated on quartz substrates, with the difference that they have a rounded contour on their geometry. When complex **2 b** was deposited on quartz, it generated a topography of dispersed particles with a height of 25–55 nm, an approximate size of 117 nm in length (being the sample with smaller particles), and a sharp circular geometry of well‐defined particles. When deposited on PET, this complex generated a visually better‐ordered structure of particles with an elongated triangular geometry, a height of 15–45 nm, and a size of approximately 200 nm in length. Finally, complex **2 c** on quartz presents a wavy structure on the surface. It stands out for not having well‐defined independent particles that seem to be connected. These have a height between 15–30 nm in height and a size of 220 nm in length. Depositing this complex on PET generated a surface dispersion of particles between 20–50 nm in height, 250 nm in length size (this is the sample with the largest particles), and a sharp circular geometry similar to those established by complex **2 b** on quartz. The data obtained by this characterization is resumed in Table 2, on which it is possible to observe the morphological differences that exist between thin films of different uranyl's as well as the morphological difference that occurs when deposits develop over different substrates. These differences can be explained by the position of the nitrogen in the pyridine of the hydroxymethylidenindanone. Apparently, this change in the position of the nitrogen generates a slight shift in the orientation of the complex molecule, which in turn causes nucleation and growth of the film in preferential shapes and directions. Concerning roughness, the film with compound **2 c** on quartz had the lowest average roughness (Ra) this can give rise to better semiconductor behavior of this film. It is important to note that the films deposited on PET present a higher roughness, which is more appreciable in the film of the **2 c** compound, however, as mentioned above, this is the thin film that presents the lowest roughness including on PET. In addition, the roughness obtained for all thin films is low, so it is considered that uranyl complexes can be deposited on PET for optoelectronic applications.

**Table 2:** Morphological properties of the uranyl(VI) thin films complexes.

	Quartz			PET		
Uranyl(VI) complex	**2 a**	**2 b**	**2 c**	**2 a**	**2 b**	**2 c**
Particles size (nm)	150	117	220	135	200	250
Particles height (nm)	25–65	25–55	15–30	25–55	15–45	20–50
Peak‐to‐valley relation (nm)	80	73	40	81	70	60
Average roughness Ra (nm)	14.3	11.1	4.7	14.7	13.5	10.7
Particles geometry	Sharp triangular	Sharp circular	Wavy	Rounded amorphous	Elongated Triangular	Sharp circular

**Figure 9:**
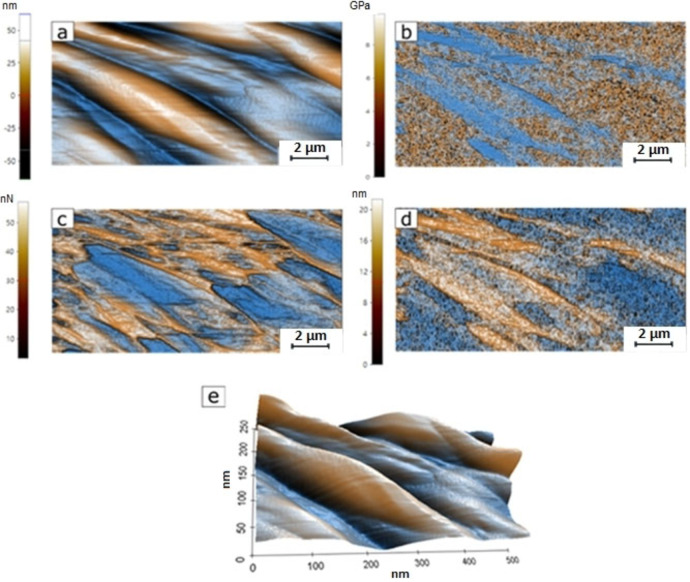
Comparison between mappings of nanomechanical properties obtained by the Pin Point technique in sample C on PET. Images of (a) topography, (b) Young's modulus distribution, (c) adhesion force distribution, (d) deformation distribution, and (e) topography with perspective.

AFM allows the measurement of nanomechanical properties through a specific surface to generate mappings of the sample characterized under each property. Using the AFM *Pin Point* test, it was possible to obtain the mapping of Young's modulus, as well as the adhesion force exerted between the surface of the analyzed complex and the AFM probe in a 500×250 nm section for each one of the samples. The mapping process allows observing how these properties are distributed on the surface of the sample and this also allows a comparison to be made between the distributions of the nanomechanical properties, concerning the surface topography of the sample, as shown in Figure 9 for the complex **2 c** on PET. Figure 9a shows a topographical recreation that is used as a reference to observe the distribution of nanomechanical properties. The first property analyzed was Young's modulus. This module allows us to determine the flexibility of the materials and the higher this is, the more rigid the material will be. That said, Young's modulus has an inverse relationship to the deformation that a material can resist. This is directly demonstrated when comparing Figure 9b and Figure 9d, in which it can be seen how the areas in which the greatest deformations occurred were those with a lower Young's modulus. Something that stands out from these mappings of Young's modulus and deformation is that the values are not constant over the analyzed surface as they follow the pattern previously established by the topography. With these mappings, it is possible to obtain the average Young's modulus of each of the complexes analyzed to determine the flexibility of the OS. In addition to the flexibility, it was possible to obtain the adhesion force that occurred between the surface and the AFM probe whose mapping can be seen in Figure 9c and, just like Young's modulus and deformation did follow the surface pattern that is observed in the topography. This information can be found in Table 3 involving Young's modulus and superficial adhesion force for all the samples. From this characterization, it was possible to determine that the sample with the highest average flexibility was made with complex **2 c** on PET, with an average Young's modulus value of 403 MPa. The least flexible sample was also the one made with complex **2 c** but deposited on quartz with a value of 1084 MPa. The variation in Young's modulus, values presented in Table 3, are an indication of the versatility in the mechanical behavior of these uranyl films. This versatility can be exploited for use in different types of optoelectronic devices, as well as in different operation conditions. Finally, in terms of adhesion force, all samples maintained an average value ranging from 11.6 nN, present in complex **2 c** deposited on quartz, to 18.1 nN found in complex **2 a** deposited also on quartz. Among these samples, those made with complex **2 b** on quartz, and complexes **2 a** and **2 b** on PET, had an equal value of 15 nN, while the one made with complex **2 c** on PET was found at 12.2 nN. This adhesion force can determine the ease with which another material can adhere to the surface of the material and the higher its value, the better adhesive qualities the material will have. According to the data presented in Table 3, the adhesion force for the different films and substrates are in the same order of magnitude and there is no significant variation between the adhesion force between PET and quartz, however, due to what was mentioned above, PET is a suitable substrate for depositing uranyl films.

**Table 3:** Morphological properties of the uranyl(VI) thin films complexes.

	Quartz			PET		
Uranyl (VI) complex	**2 a**	**2 b**	**2 c**	**2 a**	**2 b**	**2 c**
Average Young's modulus (MPa)	651	495	1084	621	612	403
Average Adhesion force (nN)	18.1	15	11.6	15	15	12.2

### Evaluation of optical and electrical properties in uranyl(VI) films

**Figure 10:**
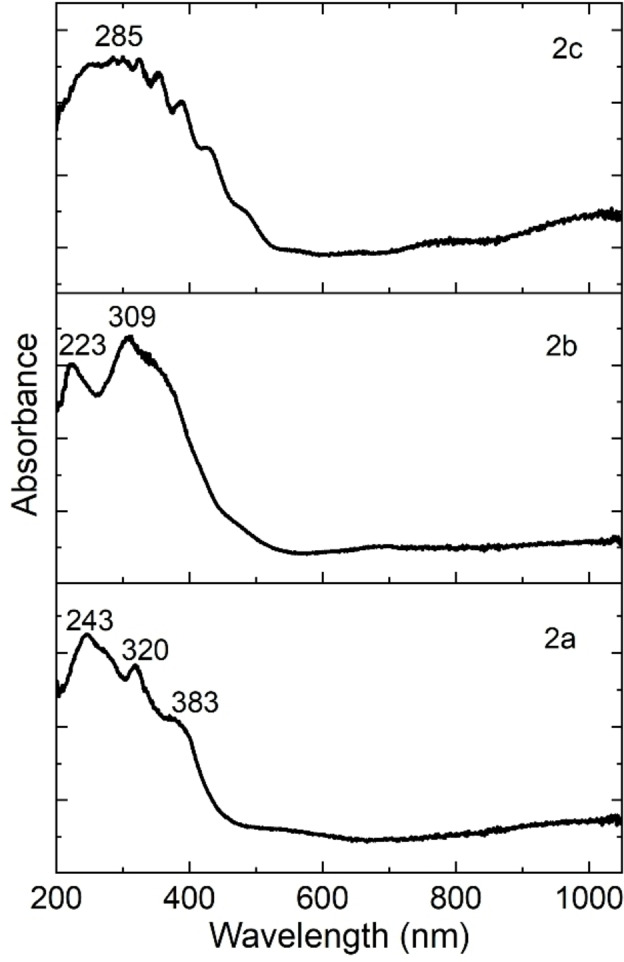
UV‐vis spectrum for uranyl films.

The absorbance in uranyl(VI) films was evaluated by measuring transmittance and reflectance, using an integrating sphere in the UV‐vis spectrophotometer (Figure 10). When comparing the UV‐vis spectra for the uranyl(VI) complexes in solution and in the solid state, marked similarities are observed for semiconductors **2 a** and **2 b**. Although shifted towards the blue of the spectrum, these results are consistent with those obtained for these same complexes in solution. However, the spectrum of film **2 c** shows a broad absorption band in which the characteristic signals that were presented in solution are not distinguished. If it is considered that the manufacture of the three thin films was carried out with (i) the same operating parameters in the evaporation system, (ii) the same quantities of the uranium complex, and (iii) the same type of substrates, then the differences in the UV‐vis spectrum are due to the different structures of the complexes. The position of the nitrogen atom in the radical pyridine is a determinant factor in the optical behavior of these complexes.

**Figure 11:**
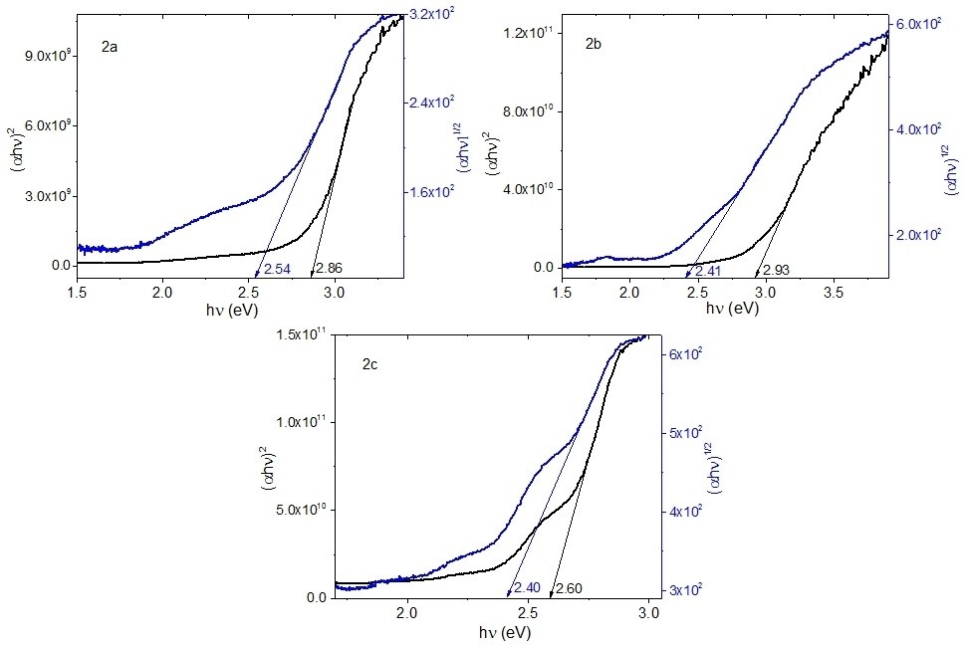
Optical bandgap plots for uranyl films.

The parameter that provides useful information to quantify the properties of the OS and their behavior in thin film devices is the energy difference between the frontier orbitals Highest Occupied Molecular Orbital (HOMO) and Lowest Unoccupied Molecular Orbital (LUMO) or bandgap.^[46]^ Most charge transitions in OS occur between the HOMO and the LUMO, and the bandgap represents the region between them. By using UV‐vis spectroscopy is possible to obtain the value of the optical bandgap (E_opt_) interval in thin films that is attributed to the transition of lower energy that takes place by absorption of a photon. For the calculation of the energy spacing, determined by UV‐vis spectrophotometric measurements, different methods can be applied, among them, it is possible to relate the bandgap of OS films to its absorption coefficient (α) and incident photon energy (*hν*) as:[^47^, ^48^] 
(1)

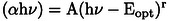




where, E_opt_ is the optical bandgap, and A depends on the type of transition, r is the power that can take 2 and 1/2 for indirect and direct electronic transitions respectively films.[^47^, ^48^] The frequency (ν ) and α are experimentally obtained from $\nu =\frac{c}{\lambda }$ and $\alpha =ln\left(T/d\right)$ respectively. Where c is the speed of light, λ is the wavelength, T is the transmittance and d is the thickness of the films. To calculate α, the thicknesses are 350, 240, and 101 nm for **2 a**, **2 b**, and **2 c** films respectively. It is important to consider that according to Cesaria et al.,^[49]^ the α of the films is an important starting point in the understanding of its optical properties for device design as well as for fundamental studies. Figure 11 shows (α*h*ν)^2^ and (α*h*ν)^1/2^ as a function of the *hν* and the Bardeen E_opt_ value could be calculated by extrapolating a tangent line to the *hυ* axis in (αhν)^2^=0 and (αhν)^1/2^=0 for direct and indirect electronic transitions respectively. Table 4 shows the optical bandgap for both types of transitions, and E_opt_ follows the progression **2 a**>**2 b**>**2 c**, which places compound **2 c** with the smallest HOMO‐LUMO energy gap. The bathochromic shift of the spectra obtained from the thin films of uranium complexes has the reduction of the optical spacing associated. As it was expected, the difference in the structure is an important factor in the bandgap value of uranyl(VI) complexes. Another factor that also, apparently influences the bandgap is the roughness,^[49]^ which, according to the results reported in Table 2, is lower for the film with compound **2 c**. It is also important to consider that according to the graphs in Figure 11, the dominant transitions are of an indirect type, related to amorphous films,[^47^, ^48^] and that although film **2 b** shows crystallinity, it is not enough to modify the electronic transitions. Furthermore, it is important to consider that all the uranyl(VI) films have a bandgap in the visible range which extends from about 2.4 to 2.93 eV, this opens the possibility of using them in light‐emitting diodes, due to its bandgap existing in the visible range.^[50]^ On the other hand; the bandgap obtained for the three uranium compounds is in the same order of magnitude as the bandgap obtained (between 2.76 and 2.90 eV) for similar compounds such as copper(II) complex of 2‐benzylidene‐1‐indanones derivatives.^[36]^ This may be an indication of the greater importance of the chemical structure coordinated to the metal atom, concerning the type of atom.

**Table 4:** Optical bandgap and Urbach energy for uranyl complexes in film.

	**2 a**	**2 b**	**2 c**
E_opt_ direct (eV)	2.54	2.41	2.40
E_opt_ indirect (eV)	2.86	2.93	2.80
E_U_ (eV)	0.58	0.51	0.44

Optical bandgap and Urbach energy (E_U_) have been studied to determine the kind of electronic transitions in OS. The reduction in optical bandgap endorses the growth of disordering in the uranyl(VI) complexes films, this can be established by calculating the tail bandwidth of the localized states (LS) in the E_U_.^[51]^ The E_U_ can be determined according to the equation 2:[^51^, ^52^, ^53^]

(2) $$\alpha =A_{a}exp\left(\frac{hv}{E_{U}}\right)$$

**Figure 12:**
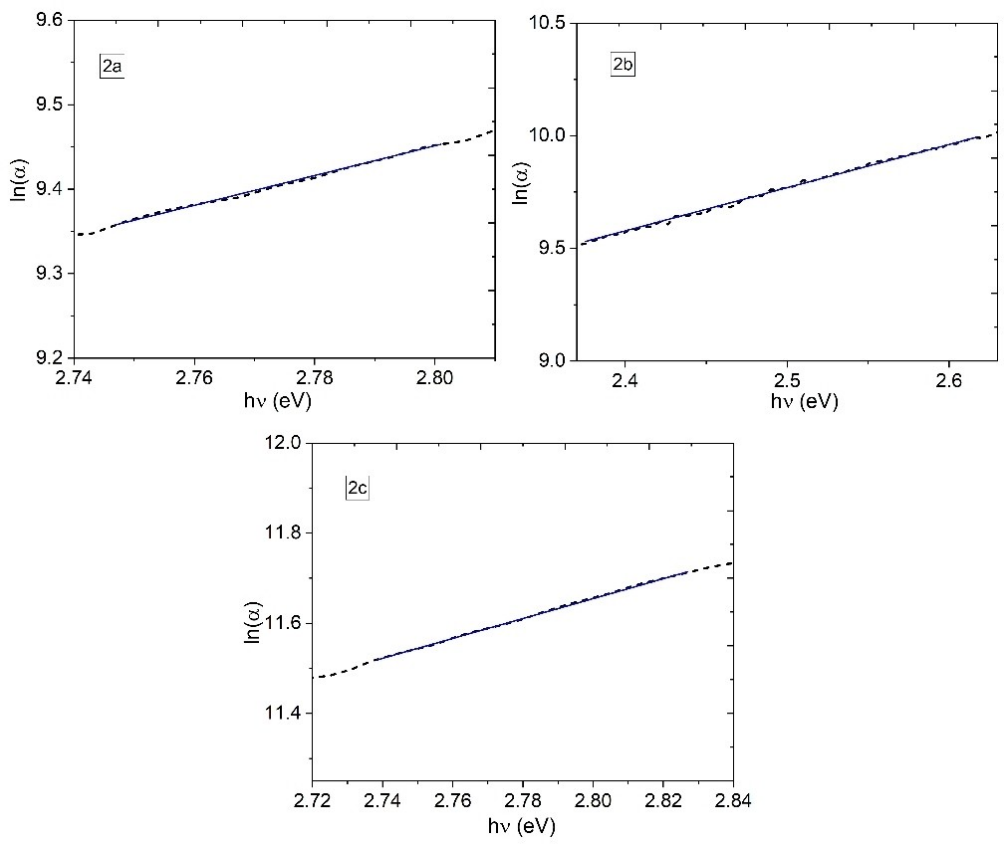
ln(*α*)‐*hν* relation of the uranyl(VI) complexes films.

In addition to the parameters defined above, A_a_ is a constant of the material that constitutes the α at the *E* ^*opt*^. The exponential absorption edge can be interpreted as due to the exponential distribution of LS in the energy bandgap.[^51^, ^53^] Figure 12 displays the relation between ln(*α*) and *hν* for the semiconductor films. The values of the E_U_ were determined from the reciprocal of the slope of the linear portion, below the optical bandgap and have been recorded in Table 4. The value of E_U_=0 is assigned to perfect semiconductors^[54]^ and therefore, the film with the **2 c** complex is the one that apparently has the least number of structural defects. As noticed from Table 4, the E_U_ is bigger in **2 a**, there is an increase in the disordering structure degree and the LS in this film. The width of *E_U_* depends on the position of nitrogen in the pyridine, apparently, the N in 4‐position generates the least number of defects during the deposit of the complex. It is important to mention that the values obtained, reported in Table 4, are lower than for some OS films such as; the PVOH (polyvinyl alcohol) film embedded with Bi‐nanoparticles (0.604 eV)^[51]^ or the hydroxypropyl methyl cellulose film (0.556–0.874 eV)^[55]^ and are similar to those obtained, for example, by Zoromba et al.^[56]^ for o‐phenylene diamine dihydrochloride film (0.387 eV). This is an indication that uranyl(VI) films present E_U_ in the range of those obtained for OS films, and even with the presence of defects that can act as traps that have the potential to transport charge between their HOMO and their LUMO.

**Figure 13:**
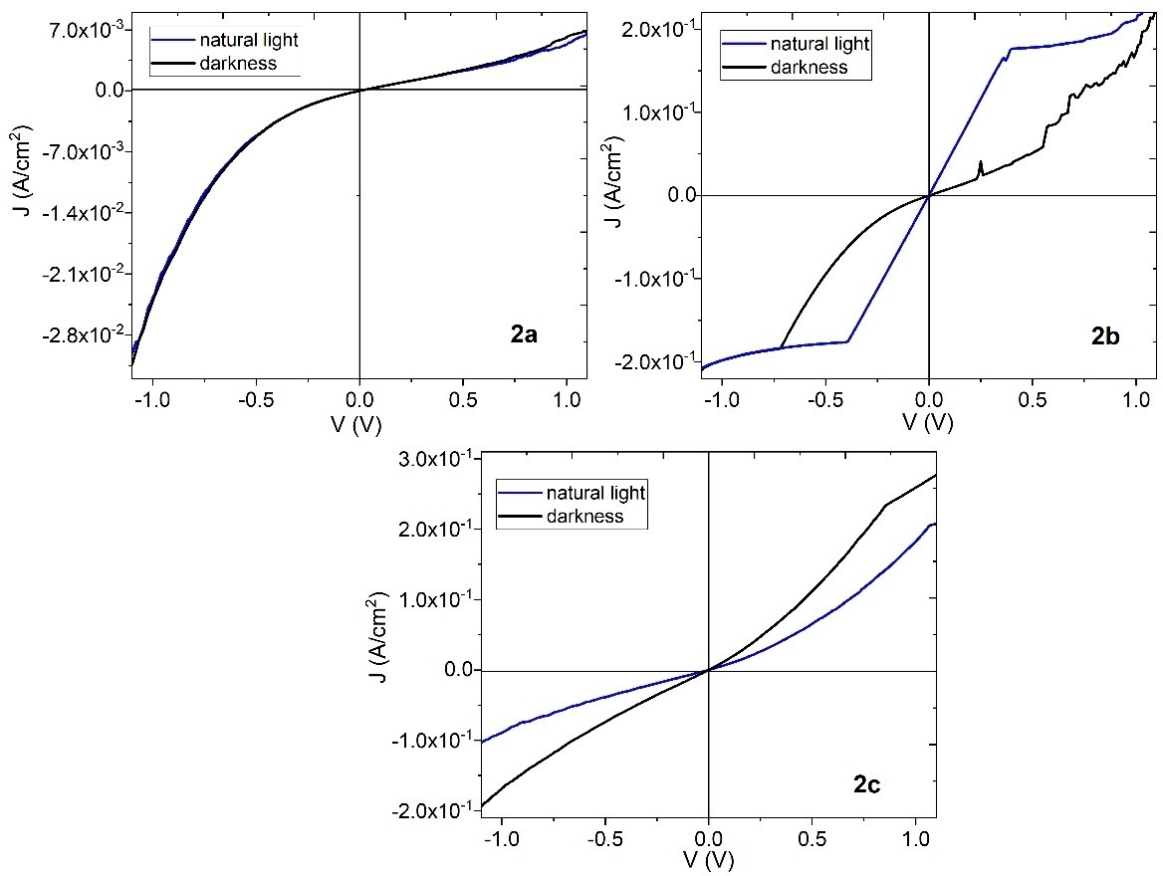
Electrical behavior (J–V), of **2 a, 2 b** and **2 c** uranyl(VI) films.

Finally, the individual knowledge of the charge transport properties of the different uranyl(VI) complexes is very useful if their use is required in optoelectronic applications. For this purpose, considering a cross‐section area of 5.77 mm^2^, the electrical characterization of the uranyl(VI) films deposited on glass was carried out. The electrical evaluation was performed in natural light and dark conditions and, as observed in Figure 13, in negative and positive voltage intervals. The above is to determine if uranium complexes show ambipolarity. However, in the current density‐voltage (J−V) graphs, it is observed that the devices do not present ambipolarity. The film of complex **2 a** is not affected by dark or natural lighting conditions, the dependence between J and V is practically exponential, and the maximum current carried in the order of 10^−3^ A/cm^2^, are similar to those obtained in films of similar compounds such as copper(II) complexes derived from 2‐benzylidene‐1‐indanones.^[41]^ Regarding the films of complexes **2 b** and **2 c**, in Figure 13 a greater transported J is observed, by practically two orders of magnitude, concerning film **2 a**. A greater J is also observed during lighting conditions, which means that **2 b** and **2 c** films have a photoconductive effect, and their conductivity is increased due to the applied light. Additionally, under natural lighting conditions, an ohmic behavior is observed in the curves at voltages less than 0.40 V and 0.85 V for **2 b** and **2 c** respectively; subsequently, a change in the slope of the curves occurs, which is an indication of space‐charge limited current (SCLC) behavior. This SCLC regime is generated because by increasing the voltage, a situation is reached in which the charge carriers do not move fast enough and accumulate in certain regions of the heterogeneous films. Therefore, the electrical behavior of these films is due to the electronic delocalization generated in the chemical structure of the uranyl complexes, the presence of oxygen in the structure of the complexes and its high capacity to attract electrons, and finally, the position of the nitrogen in the pyridine of the hydroxymethylidenindanone.

## Conclusions

A series of uranyl(VI) organic semiconductors derived from 2‐methylidene‐1‐indanones were synthesized and characterized by IR spectroscopy, NMR spectroscopy, mass spectrometry, and X‐ray diffraction. Thanks to their high thermal stability, the semiconductor films of uranyl(VI) complexes were deposited by high vacuum deposition. This technique generated heterogeneous films with an amorphous structure in the case of **2 a** and **2 c** and with a slightly crystalline structure in the case of film **2 b**. The AFM in the uranyl films shown has different notable characteristics that vary depending on the complex used as well as the substrate on which they are deposited. In addition, this morphology proves to be related to the surface nanomechanical properties of the samples. Regarding the optical properties, the bandgap for indirect and direct electronic transitions in the films is in the Interval of 2.40 and 2.93 eV, being the minor for film **2 c**. Finally, the electrical behavior of film **2 a** shows exponential electrical behavior, with maximum current values of the order of 10^−3^ A/cm^2^. On the other hand, from the J–V curves of the **2 b** and **2 c** films, it was possible to verify that the current density is higher when the films are exposed to light, with maximum current values of the order of 10^−1^ A/cm^2^. When the dark condition was used, the current density was lower. Due to the shape of the J–V curves, the **2 b** and **2 c** films have a photoconductive effect, with ohmic behavior at low voltages and with a SCLC regime at high voltages. The position of the nitrogen in the pyridine of the hydroxymethylidenindanone exerts an important influence on the mechanical, optical, and electrical properties of uranyl(VI) complexes films. These complexes can be used in Molecular Electronics applications.

## Supporting Information Summary

The supporting information includes the IR, MS, NMR spectra of ligand **1 b** and all complexes, also the information about ORTEP **2 b** complex; for single crystal structure deposition number of **2 b** CCDC is 2260655 in Cambridge Crystallographic Data Center and Fach information szentrum Karlsruhe.

## Conflict of interests

The authors declare no conflict of interest.

1

## Supporting information

As a service to our authors and readers, this journal provides supporting information supplied by the authors. Such materials are peer reviewed and may be re‐organized for online delivery, but are not copy‐edited or typeset. Technical support issues arising from supporting information (other than missing files) should be addressed to the authors.

Supporting Information

## Data Availability

The data that support the findings of this study are available in the supplementary material of this article.
