# Supplementary material for: Design of Promising Uranyl(VI) Complexes Thin Films with Potential Applications in Molecular Electronics
Source: ChemistryOpen. 2024 Jan 5;13(6):e202300219. doi: 10.1002/open.202300219 (PMC11164027; doi:10.1002/open.202300219)
Supplement: Supplementary file 1 — Supporting Information [file OPEN-13-e202300219-s001.pdf]

ChemistryOpen

Supporting Information

Design of Promising Uranyl(VI) Complexes Thin Films with Potential Applications in Molecular Electronics

César Raúl Monzón González, María Elena Sánchez Vergara,* Milton Carlos Elías-Espinosa, Sergio Arturo Rodríguez-Valencia, Byron José López-Mayorga, José León Castillo-Arroyave, Rubén Alfredo Toscano, Octavio Lozada Flores, and Cecilio Álvarez Toledano*

Ligand 1b:

IR:

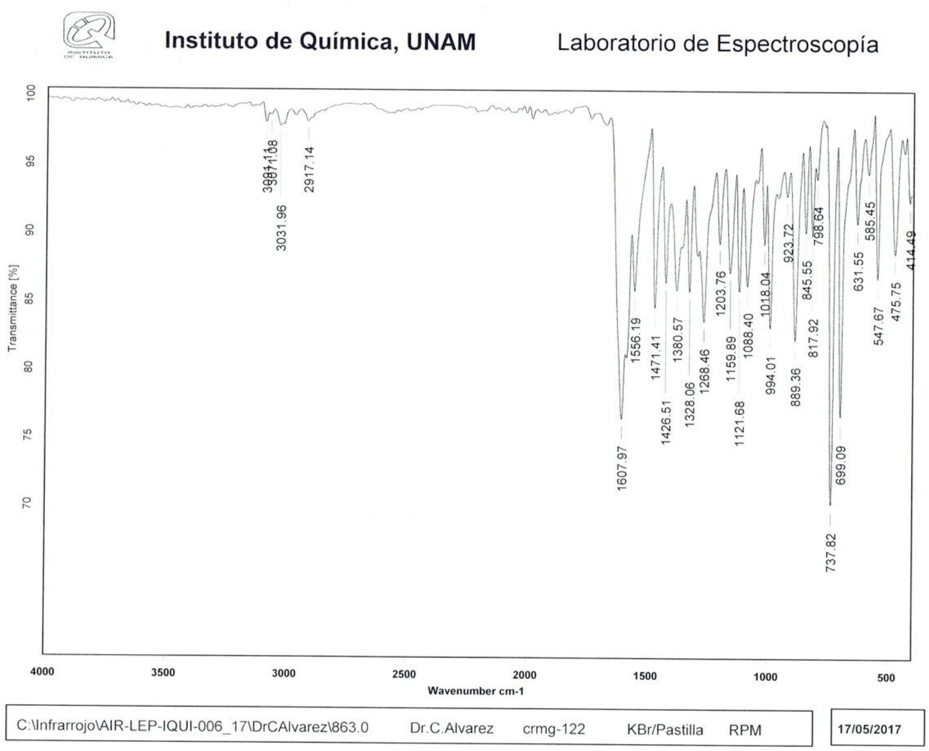

MS (DART$^+$):

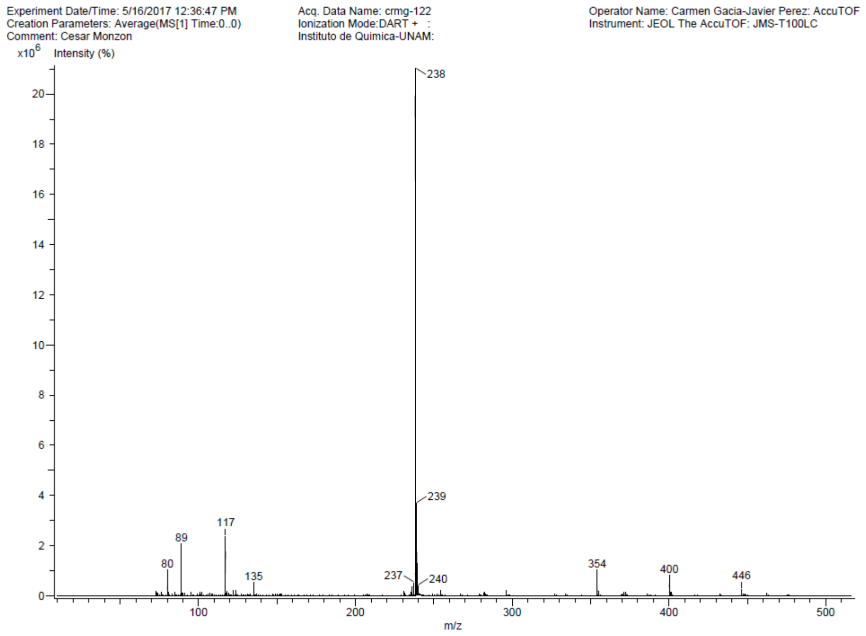

MS (ESI):

Data:crmg-122
Sample Name:Cesar Monzon
Description:
Ionization Mode:ESI+
History:Determine m/z[Peak Detect[Centroid,50,Area];Correct Base[80.0%]];Corre...

Acquired:5/16/2017 12:36:47 PM
Operator:AccuTOF
Mass Calibration data:Cal Peg_600
Created:6/9/2017 10:40:11 AM
Created by:AccuTOF

Charge number:1
Tolerance:20.00(ppm), 5.00 .. 15.00(m...
Unsaturation Number:0.0 .. 14.0 (Fracti...
Element:^{12}C:0 .. 15, ^{1}H:0 .. 13, ^{14}N:0 .. 3, ^{16}O:0 .. 3

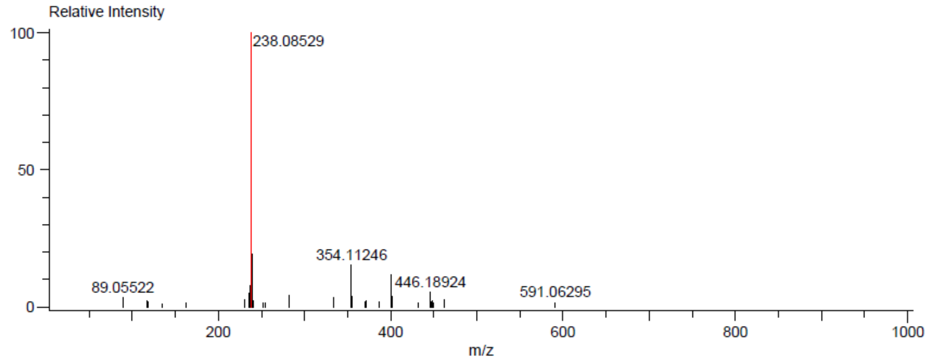

Mass	Intensity	Calc. Mass	Mass Difference (mmu)	Mass Difference (ppm)	Possible Formula
238.08529	1368936.42	238.08680	-1.52	-6.37	$^{12}C_{15}{}^{1}H_{12}{}^{14}N_{1}{}^{16}O_{2}$

^{1}H-NMR in $CDCl_3$ 300 MHz:

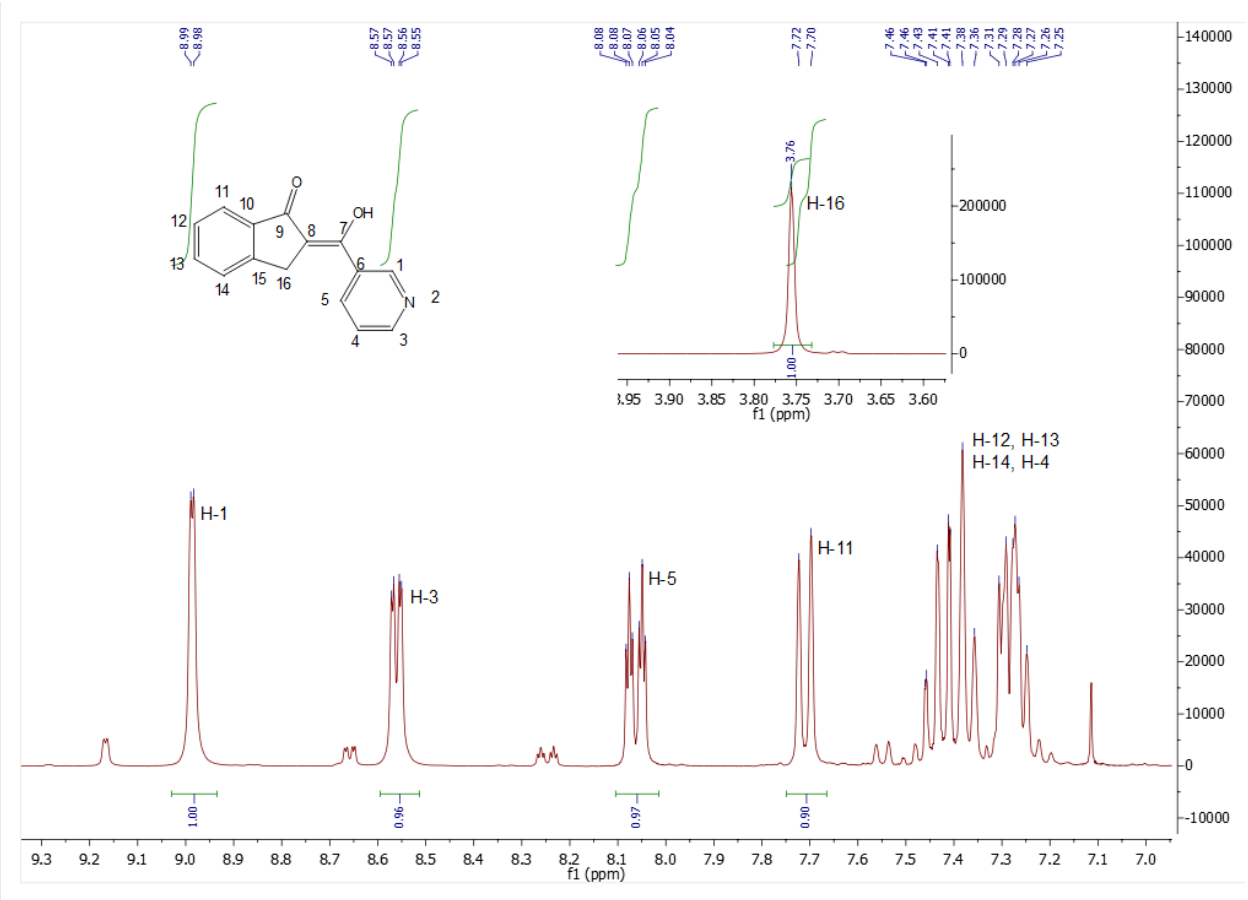

^{13}C-NMR in $CDCl_3$ 300 MHz:

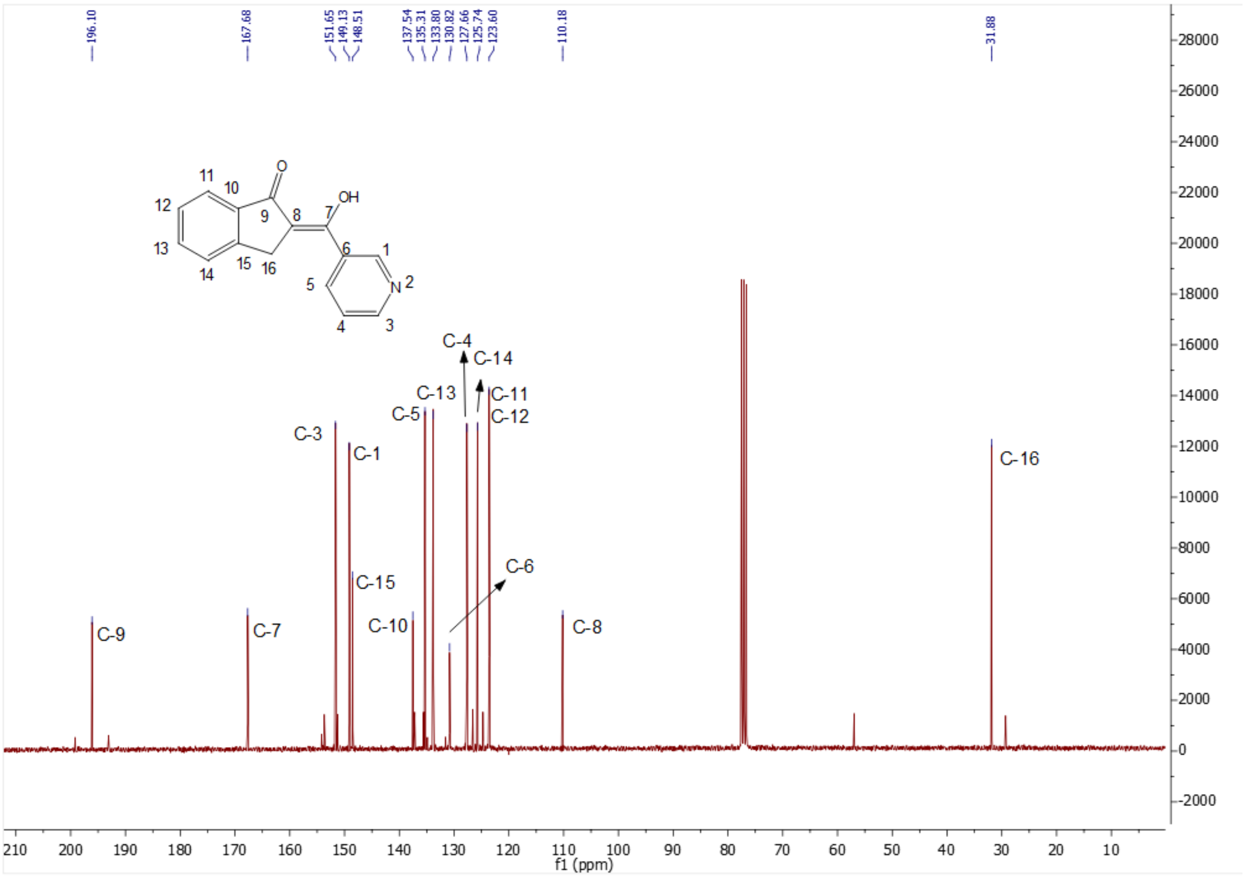

COSY in $CDCl_3$ 300 MHz:

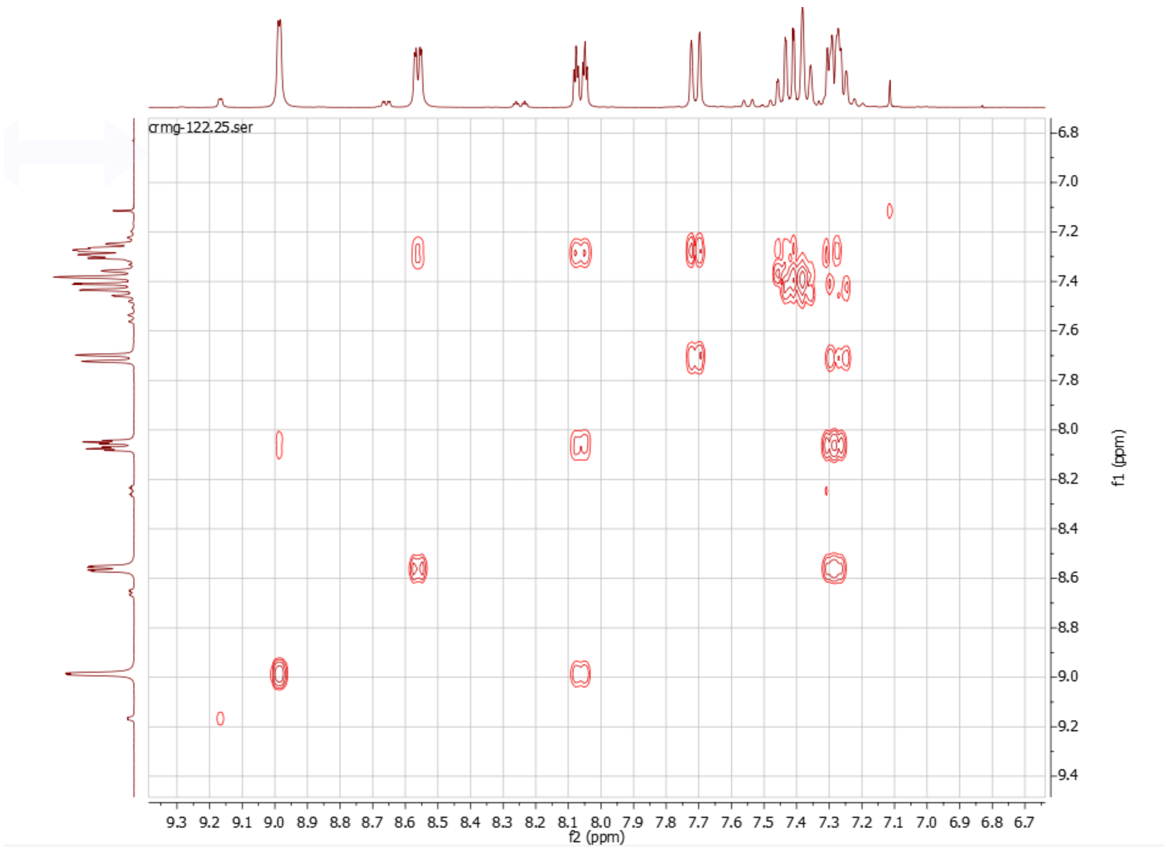

HSQC in $CDCl_3$ 300 MHz:

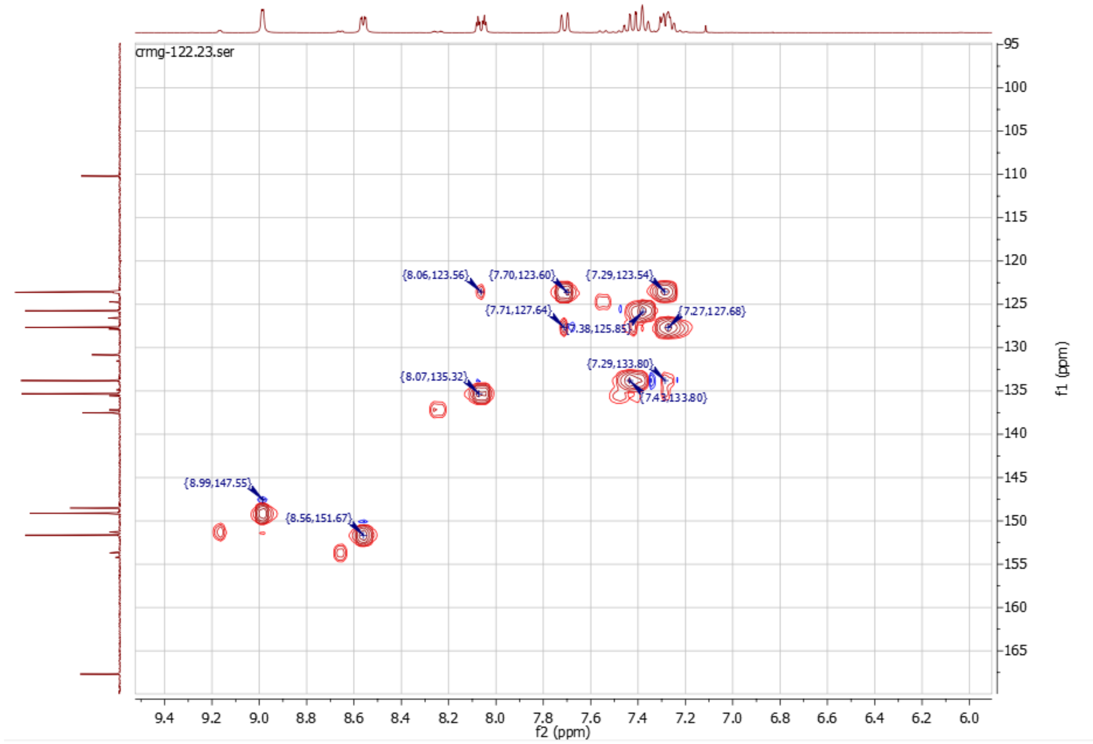

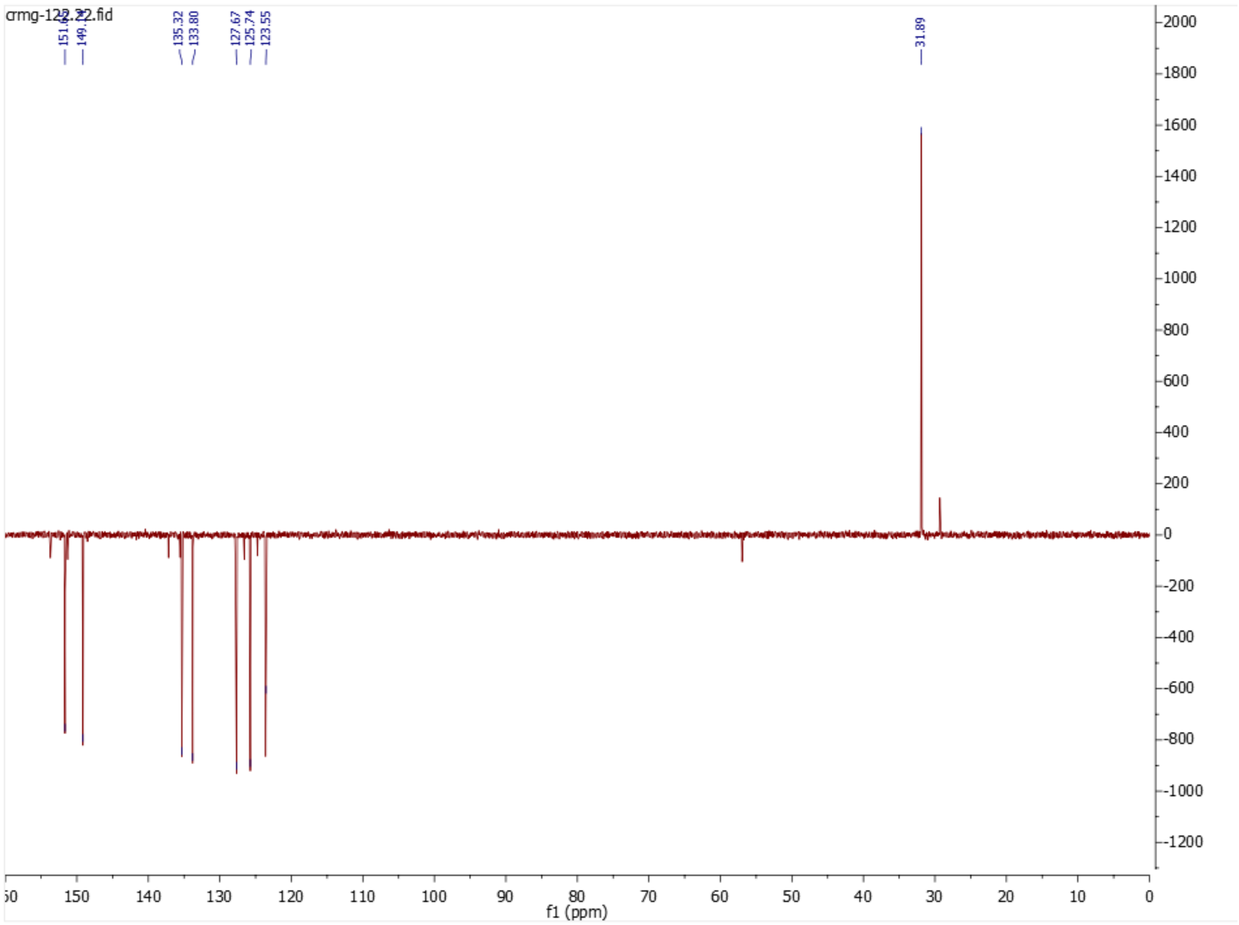

Complex 2a:

IR:

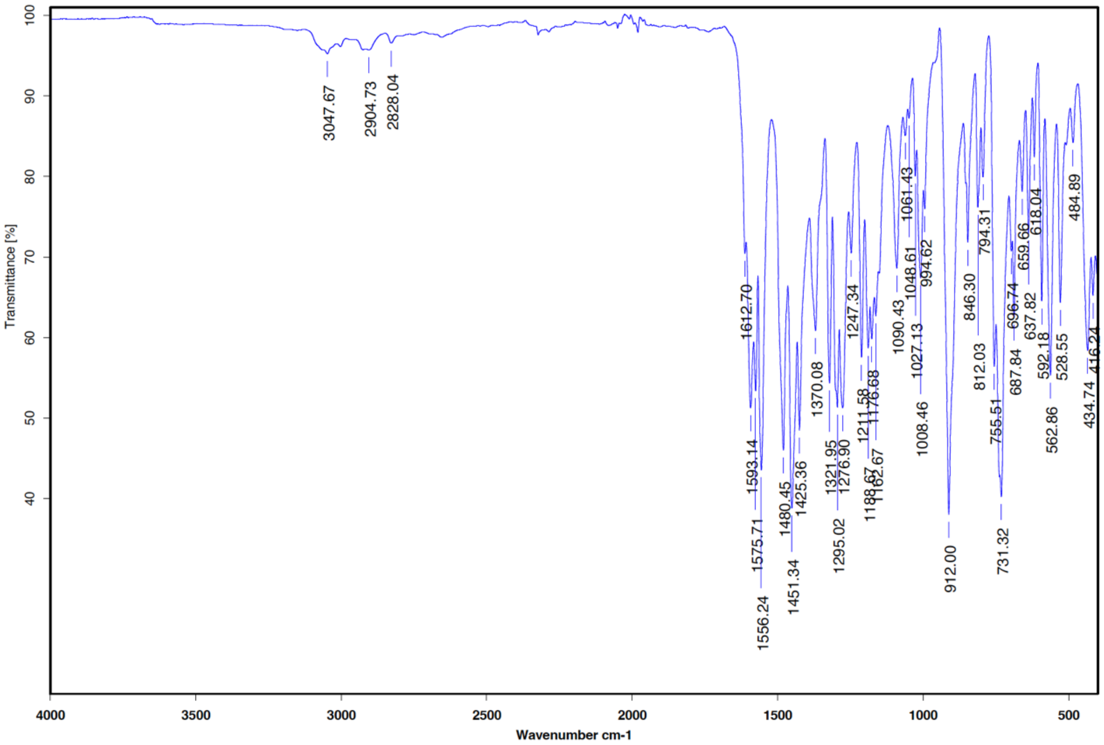

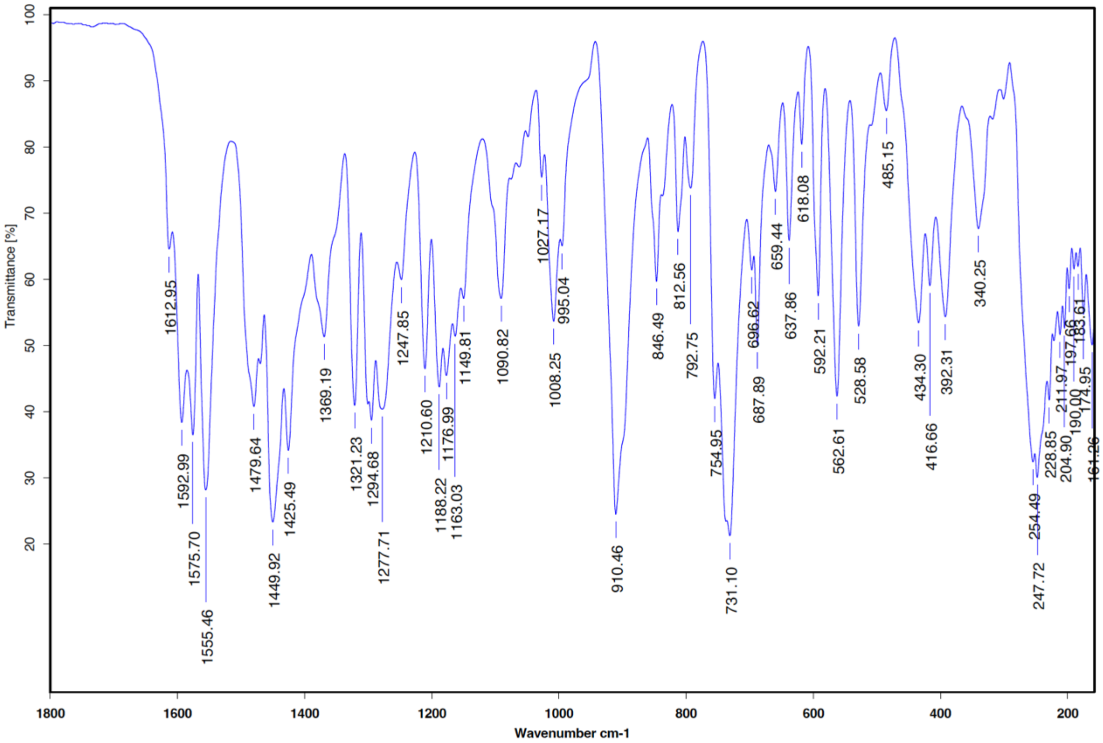

MS (FAB^+):

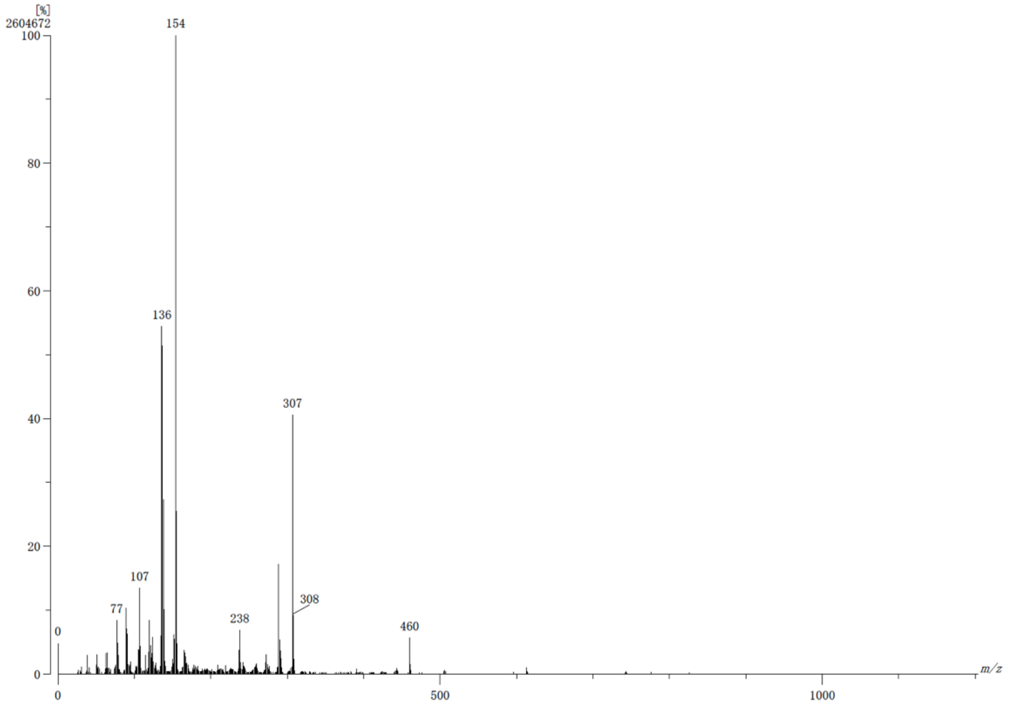

MS (ESI):

User Spectra

Fragmentor Voltage	Collision Energy	Ionization Mode
150	0	ESI

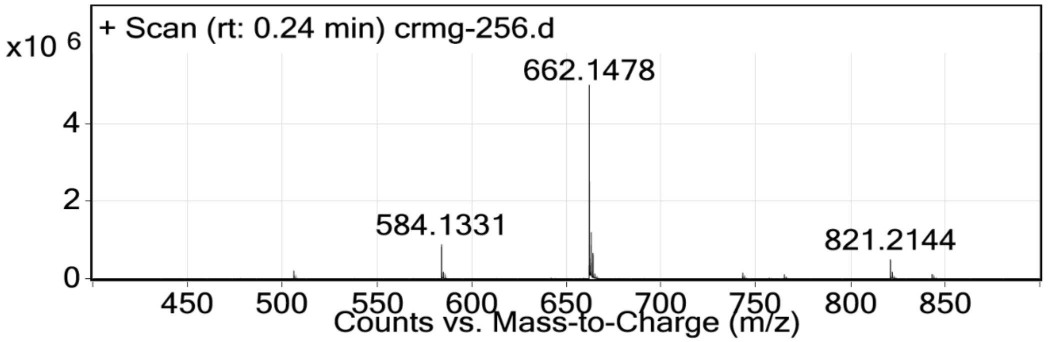

Peak List

m/z	z	Abund
584.1331		890891.47
662.1478	1	5153861.51
662.3487		400741.2
663.1515	1	1233448.2
664.1465	1	667868.88
821.2144		502281.41

^{1}H-NMR in DMSO-d6 700 MHz:

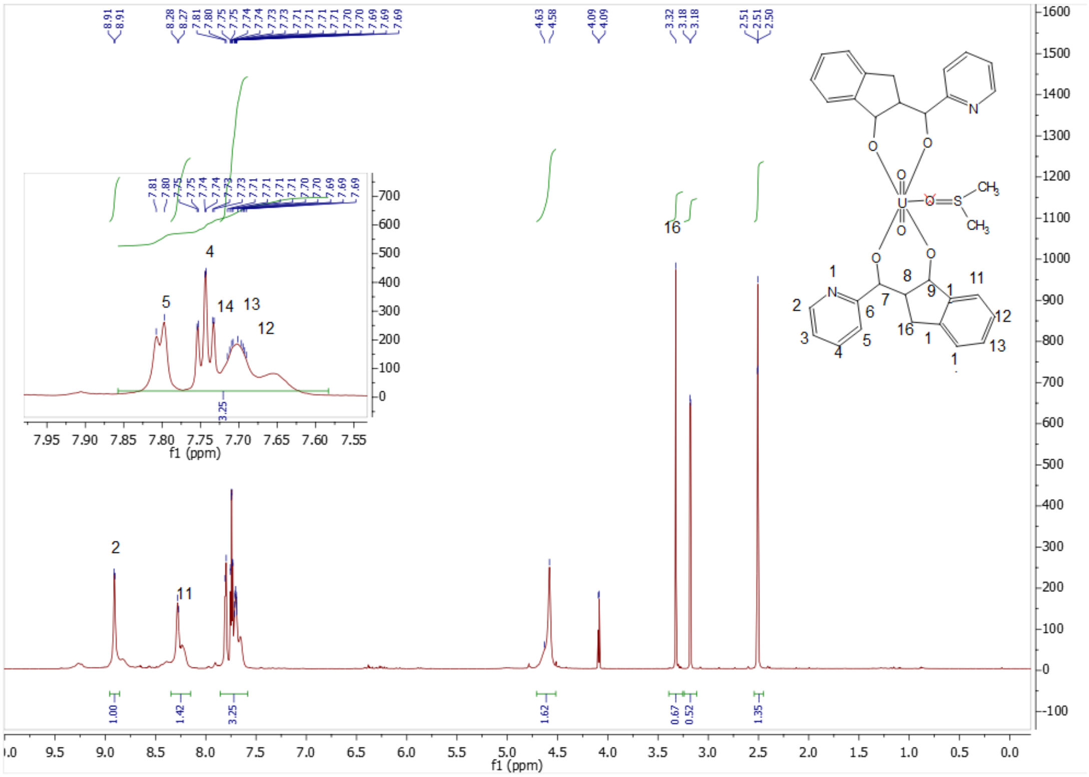

^{13}C-NMR in DMSO-d6 700 MHz

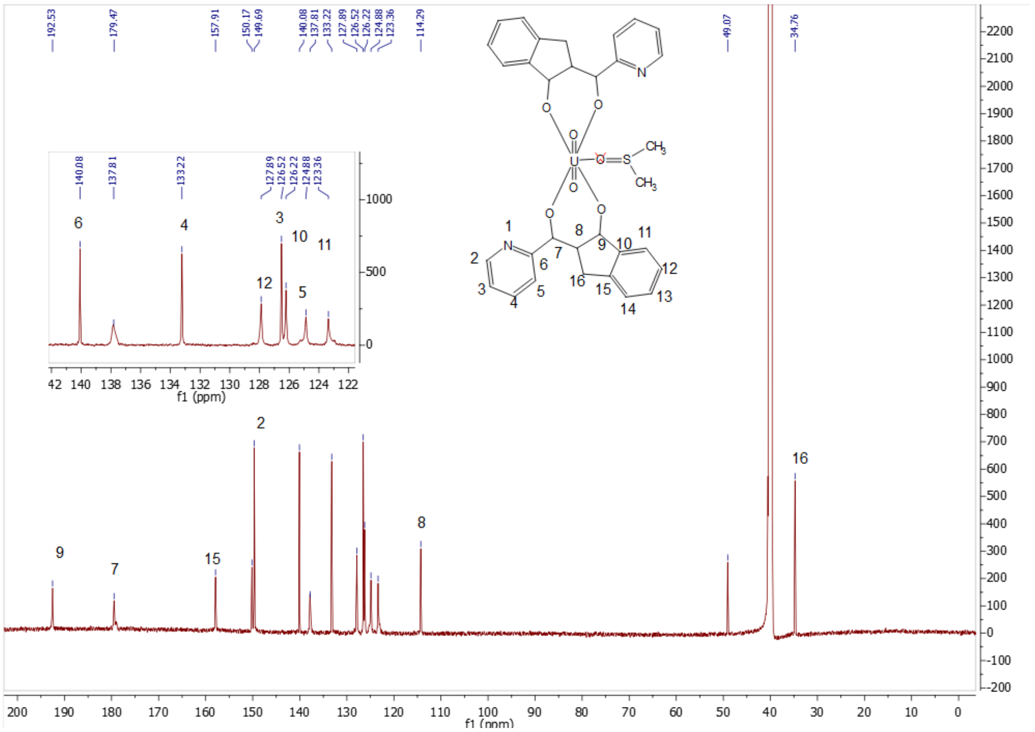

COSY-NMR ^{1}H-^{1}H in DMSO-d6 700 MHz

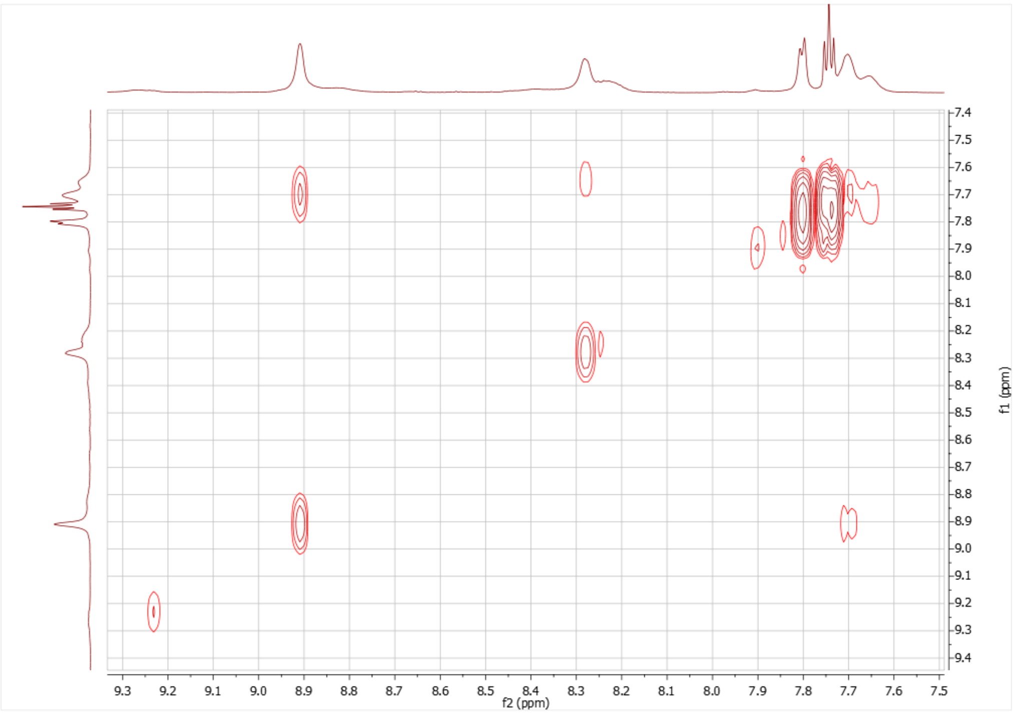

HSQC-NMR in DMSO-d6 700 MHz

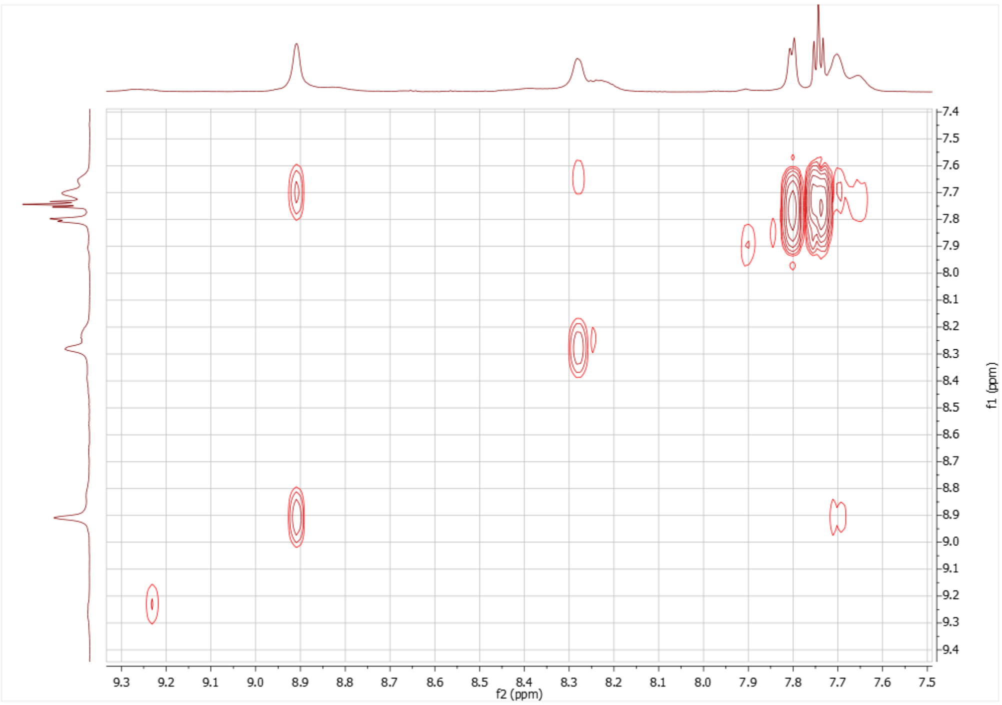

Complex 2b:

IR:

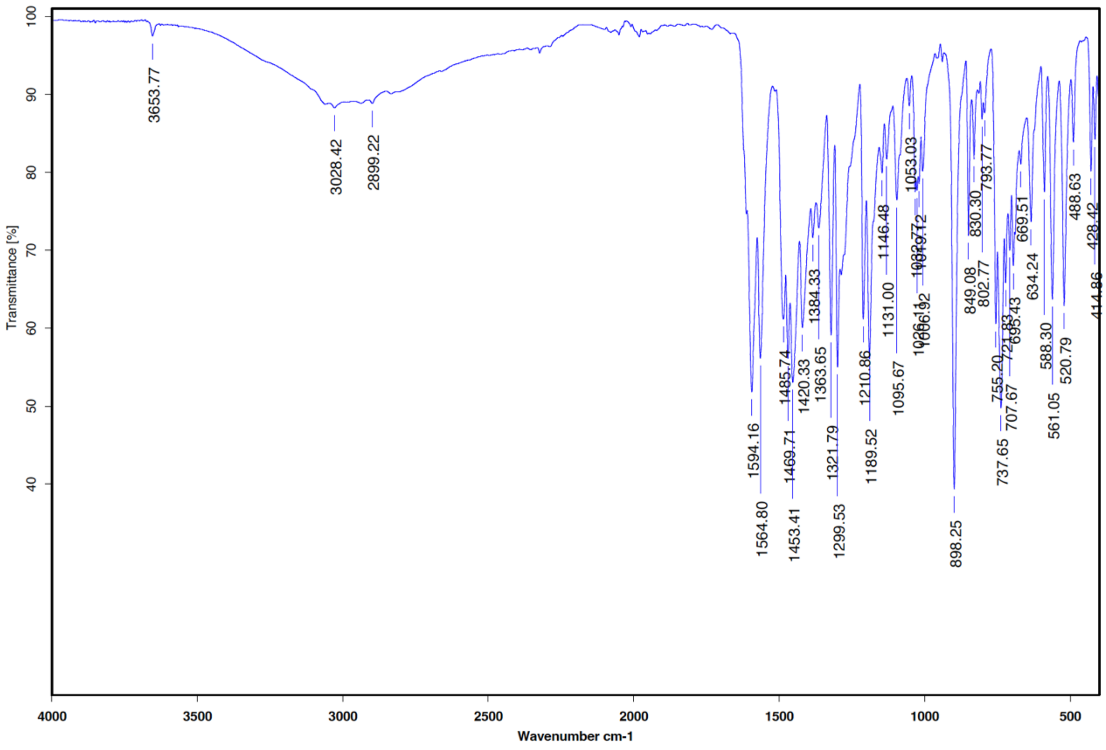
Transmittance [%]
Wavenumber cm-1
3653.77
3028.42
2899.22
1594.16
1564.80
1485.74
1469.71
1453.41
1420.33
1384.33
1363.65
1321.79
1299.53
1210.86
1189.52
1146.48
1131.00
1095.67
1053.03
898.25
849.08
830.30
802.77
793.77
737.65
707.67
669.51
634.24
588.30
561.05
520.79
488.63
428.42
414.86

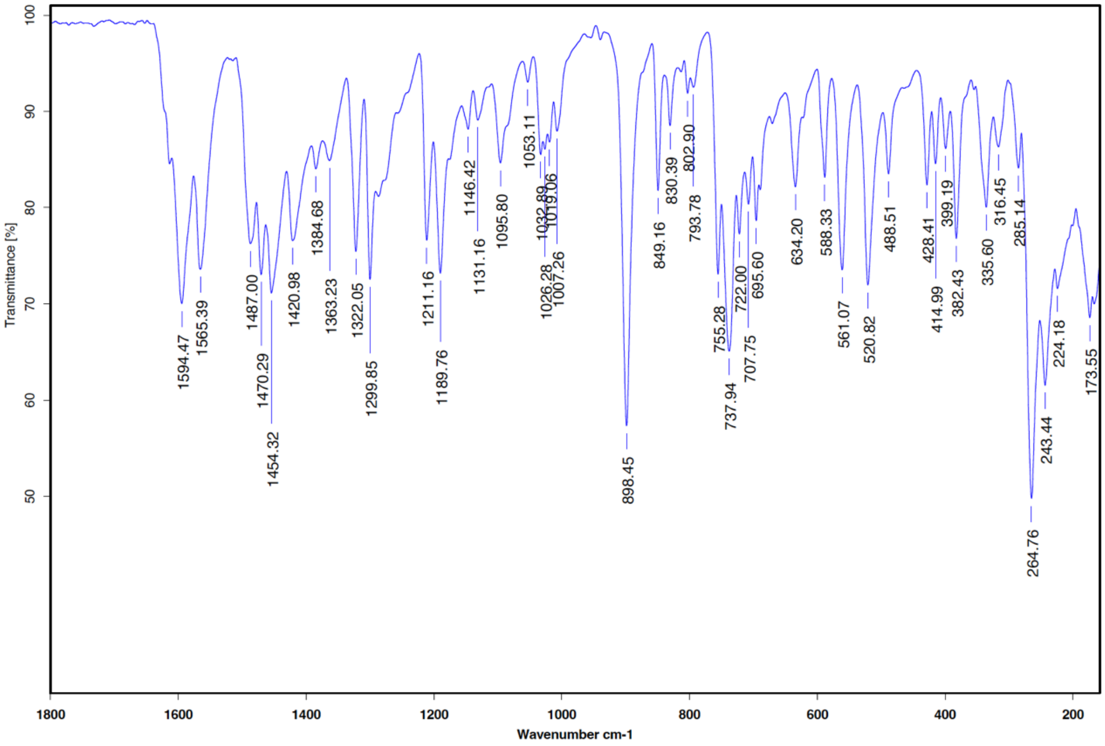
Transmittance [%]
Wavenumber cm-1
1594.47
1565.39
1487.00
1470.29
1454.32
1420.98
1384.68
1363.23
1322.05
1299.85
1211.16
1189.76
1146.42
1131.16
1095.80
1053.11
1032.89
1026.28
1019.06
1007.26
898.45
849.16
830.39
802.90
793.78
755.28
737.94
722.00
707.75
695.60
634.20
588.33
561.07
520.82
488.51
428.41
414.99
399.19
382.43
335.60
316.45
285.14
264.76
243.44
224.18
173.55

MS (FAB$^+$):

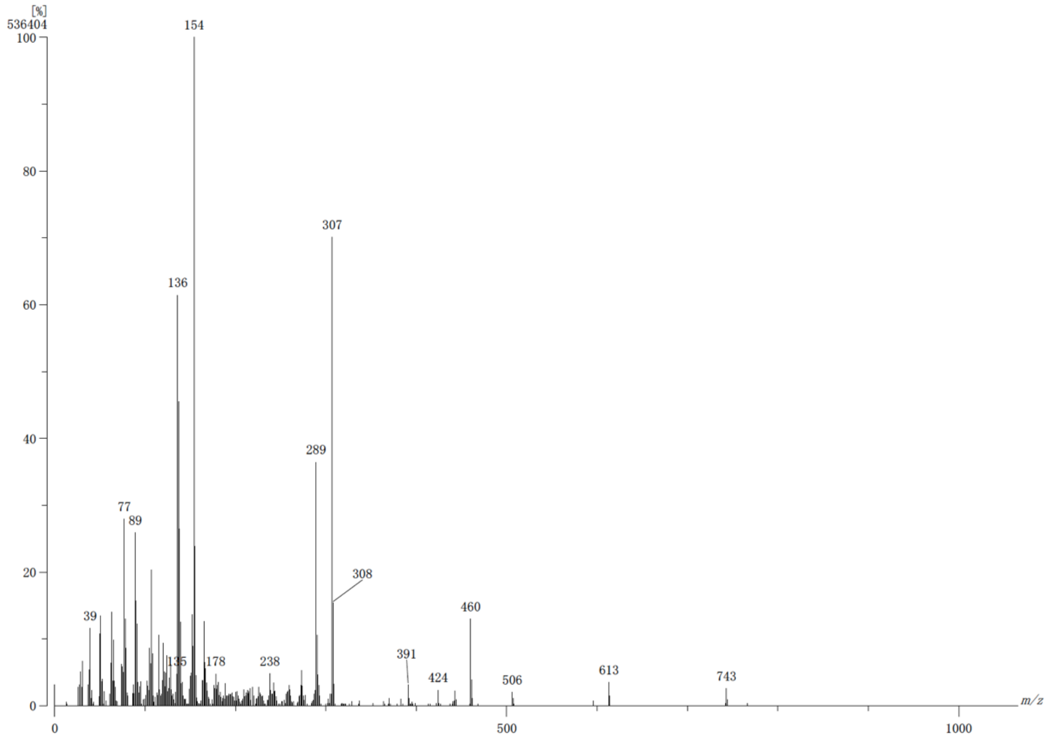

MS (ESI):

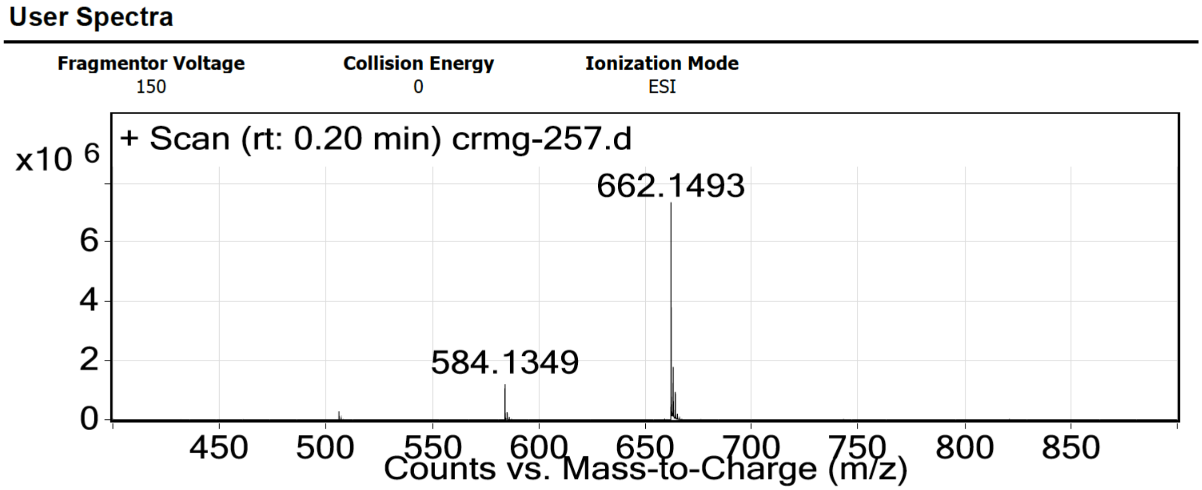

Peak List

m/z	z	Abund
584.1349		1224832.35
662.1493	1	7480261.29
662.3492		596847.05
663.1518	1	1822020.85
664.149	1	940117.73

^{1}H-NMR in DMSO-d6 700 MHz:

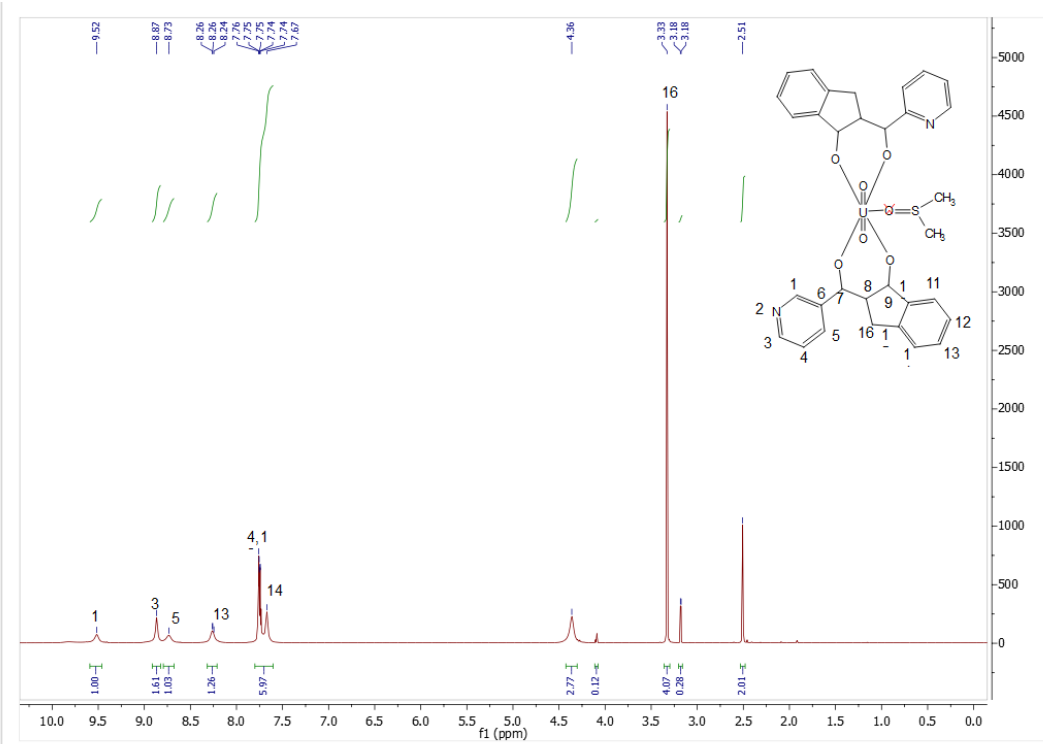

^{13}C-NMR in DMSO-d6 700 MHz:

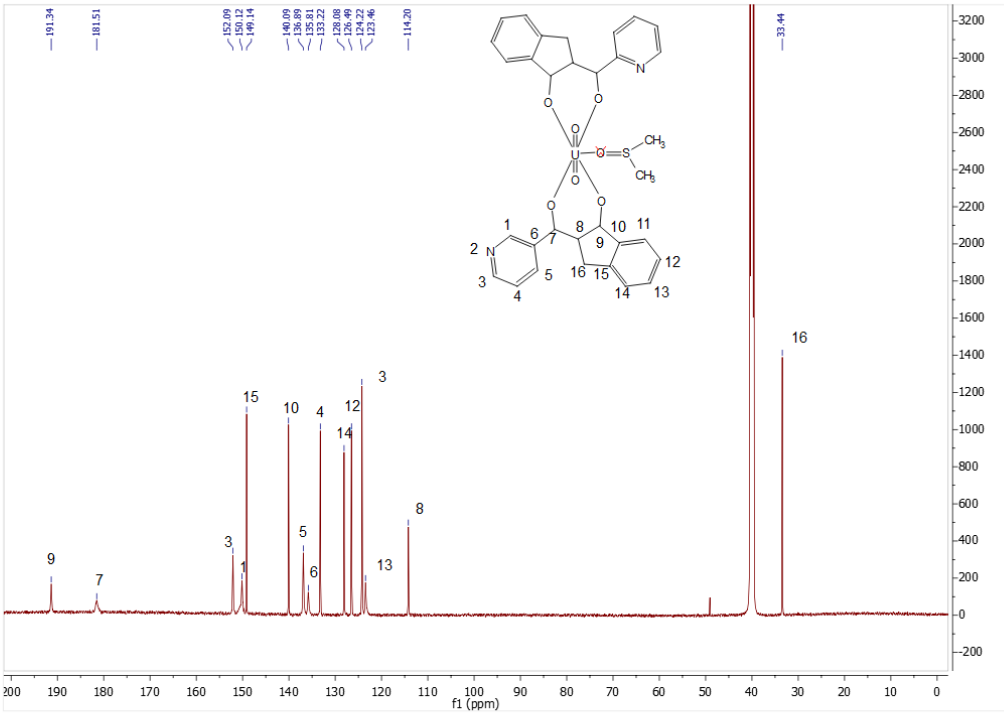

COSY-NMR ^{1}H-^{1}H in DMSO-d6 700 MHz

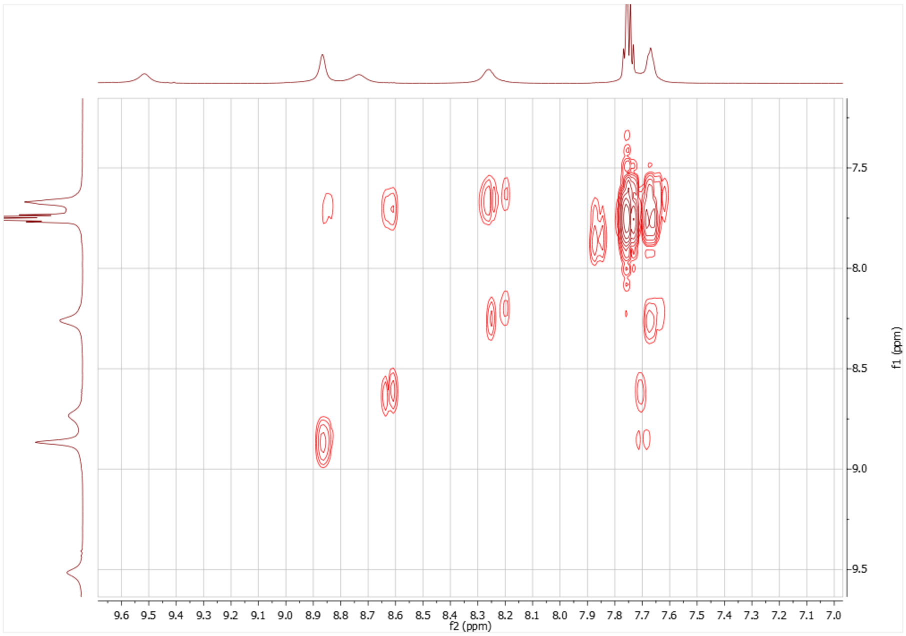

HSQC-NMR in DMSO-d6 700 MHz

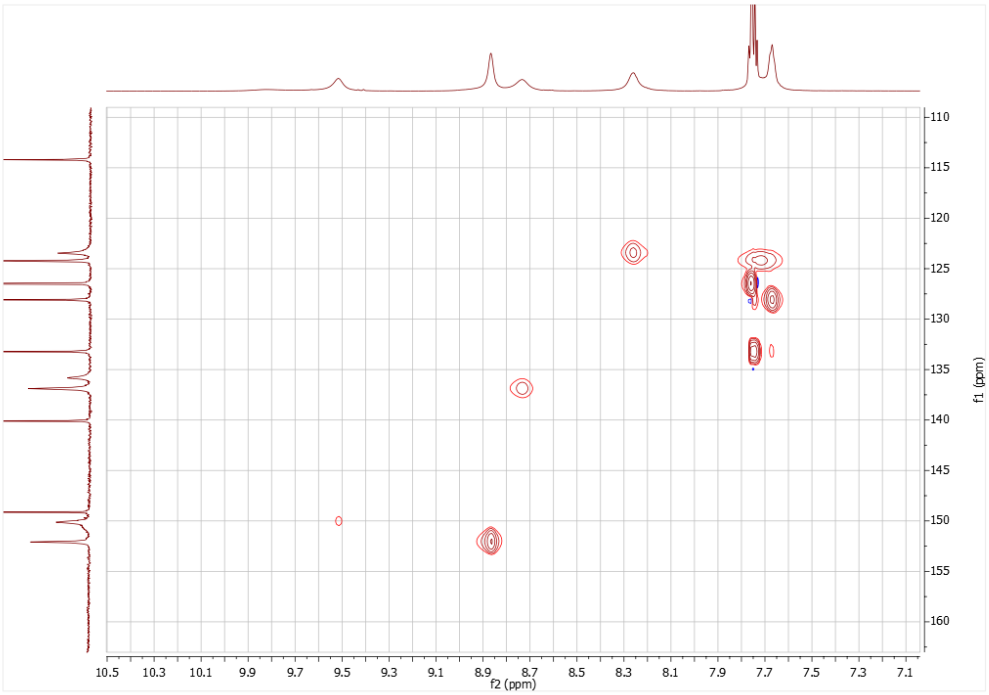

Complex 2c:

IR:

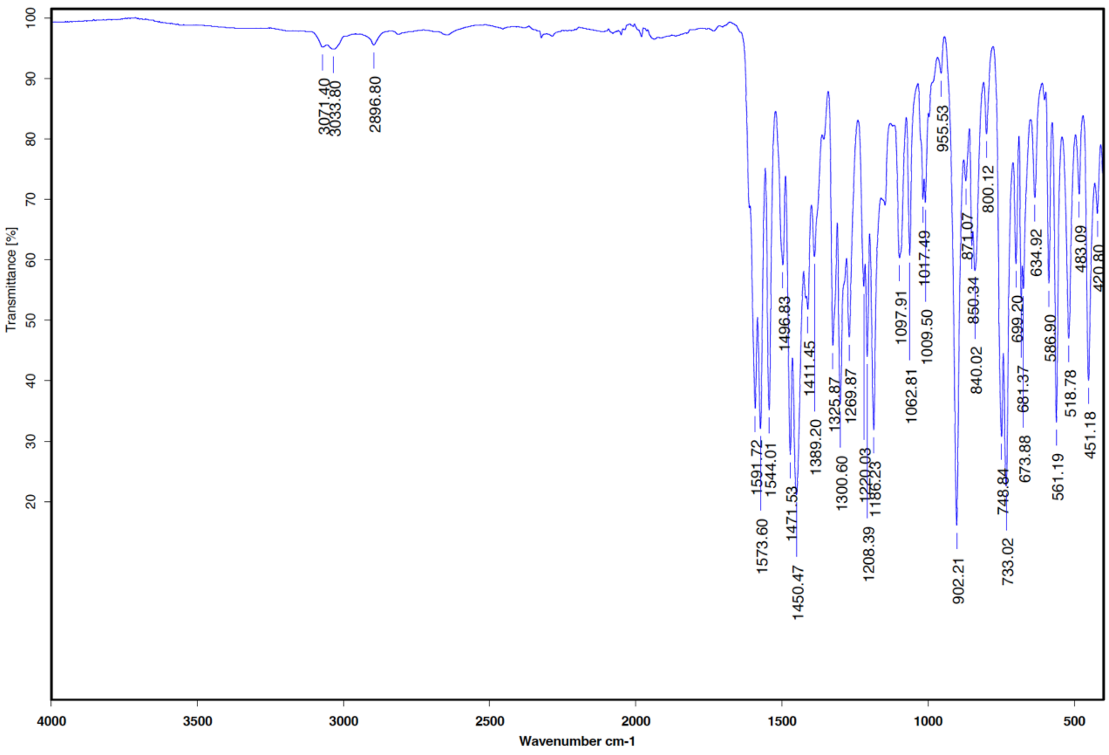
Transmittance [%]
100
90
80
70
60
50
40
30
20
3071.40
3033.80
2896.80
1591.72
1573.60
1544.01
1496.83
1471.53
1450.47
1411.45
1389.20
1325.87
1300.60
1269.87
1220.03
1208.39
1186.23
1097.91
1062.81
1017.49
1009.50
955.53
902.21
871.07
850.34
840.02
800.12
748.84
733.02
699.20
681.37
673.88
634.92
586.90
561.19
518.78
483.09
451.18
420.80
4000
3500
3000
2500
2000
1500
1000
500
Wavenumber cm-1

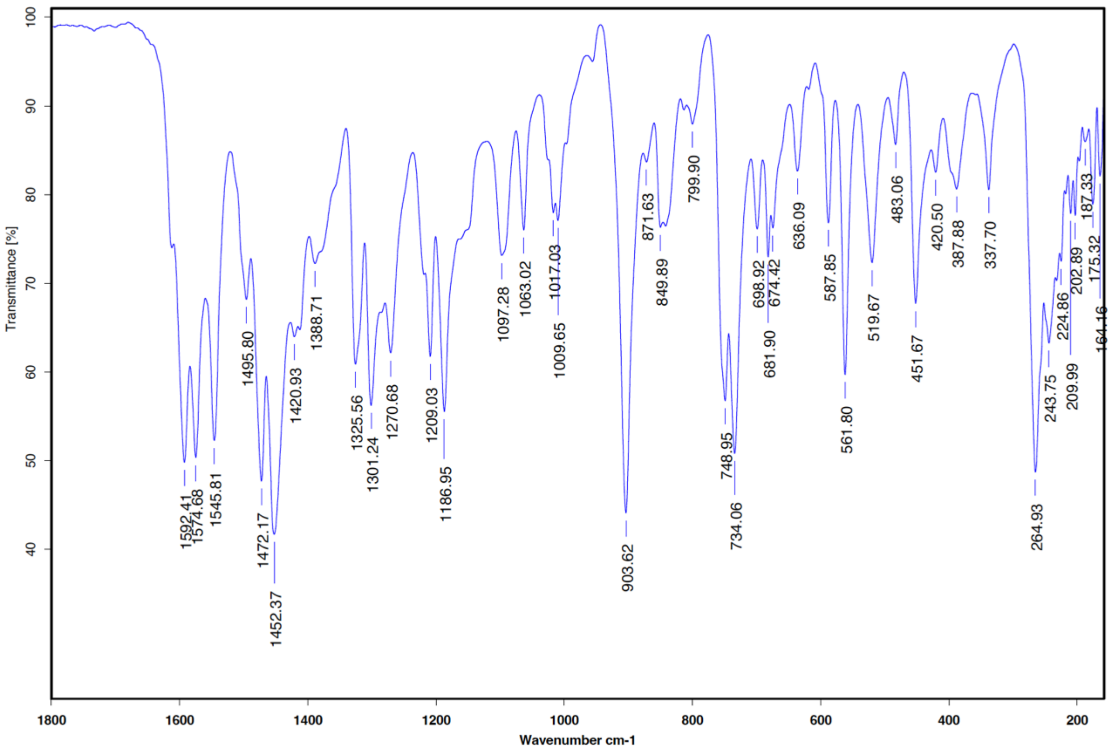
Transmittance [%]
100
90
80
70
60
50
40
1592.41
1574.68
1545.81
1495.80
1472.17
1452.37
1420.93
1388.71
1325.56
1301.24
1270.68
1209.03
1186.95
1097.28
1063.02
1017.03
1009.65
903.62
871.63
849.89
799.90
748.95
734.06
698.92
681.90
674.42
636.09
587.85
561.80
519.67
483.06
451.67
420.50
387.88
337.70
264.93
243.75
224.86
209.99
202.89
187.33
175.32
164.16
1800
1600
1400
1200
1000
800
600
400
200
Wavenumber cm-1

MS (FAB$^+$):

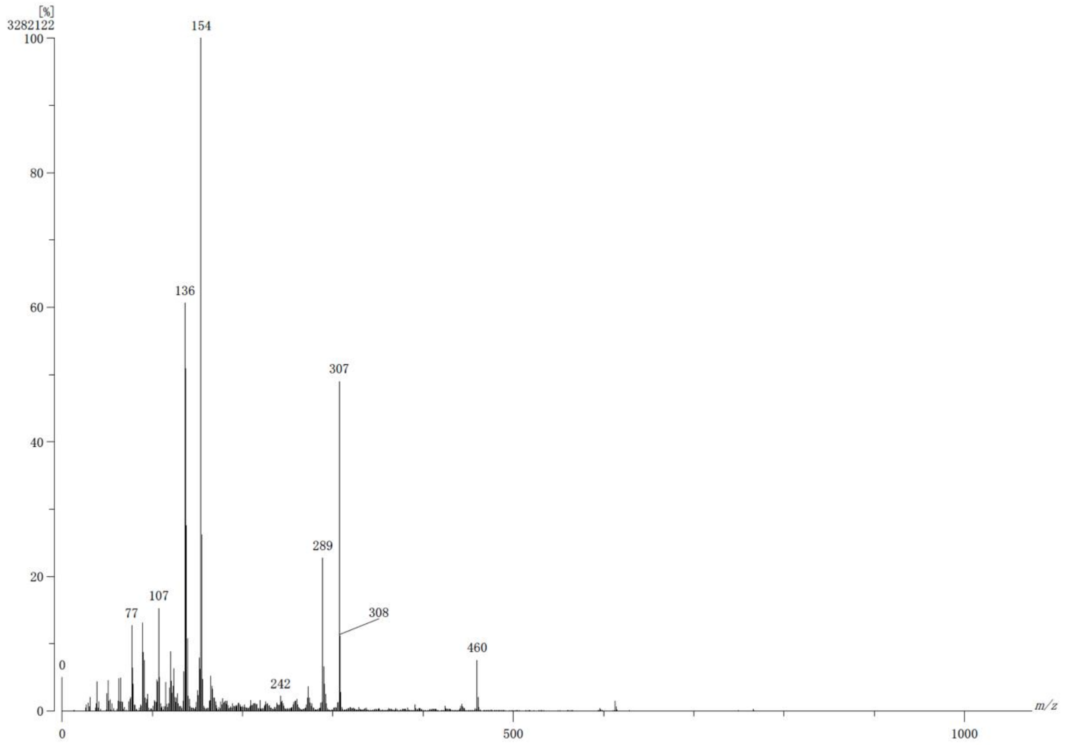

MS (ESI):

User Spectra

Fragmentor Voltage	Collision Energy	Ionization Mode
150	0	ESI

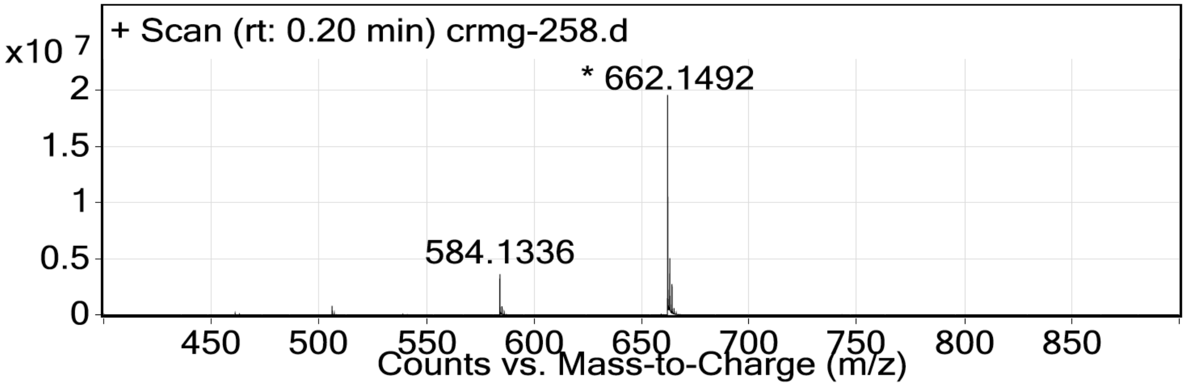

Peak List

m/z	z	Abund
584.1336		3667274.9
662.1492	1	19977948.87
662.3482		1648381.66
663.151	1	5197813.13
664.1471	1	2733768.66

^{1}H-NMR in DMSO-d6 700 MHz:

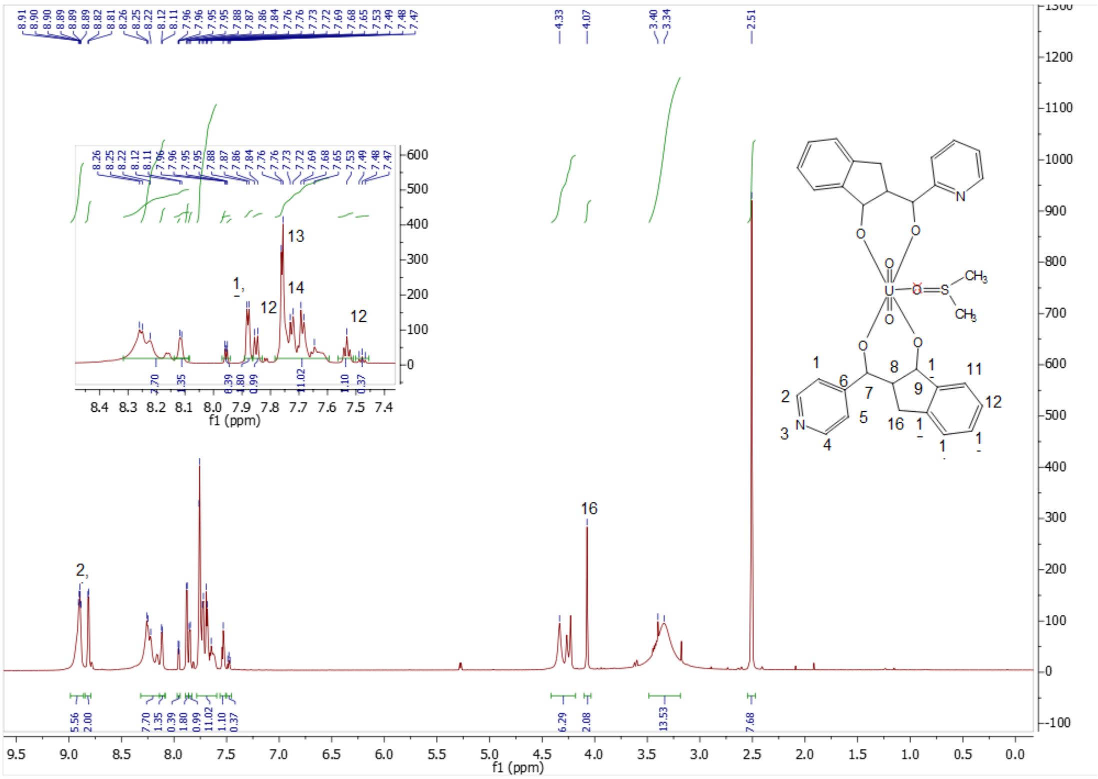

^{13}C-NMR in DMSO-d6 700 MHz:

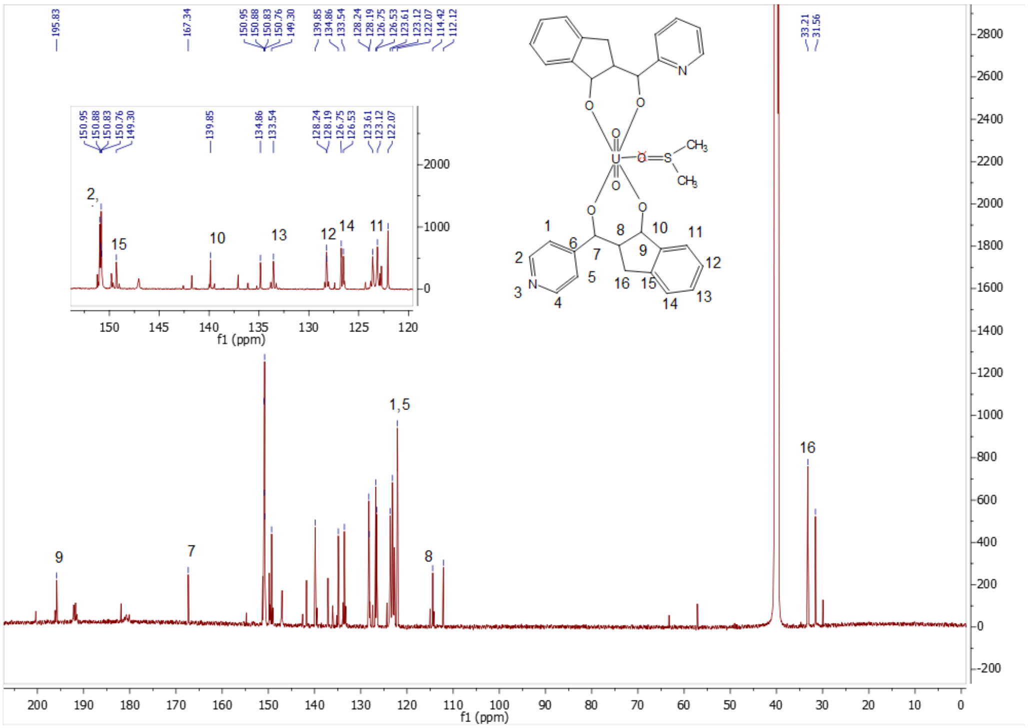

COSY-NMR ^{1}H-^{1}H in DMSO-d6 700 MHz

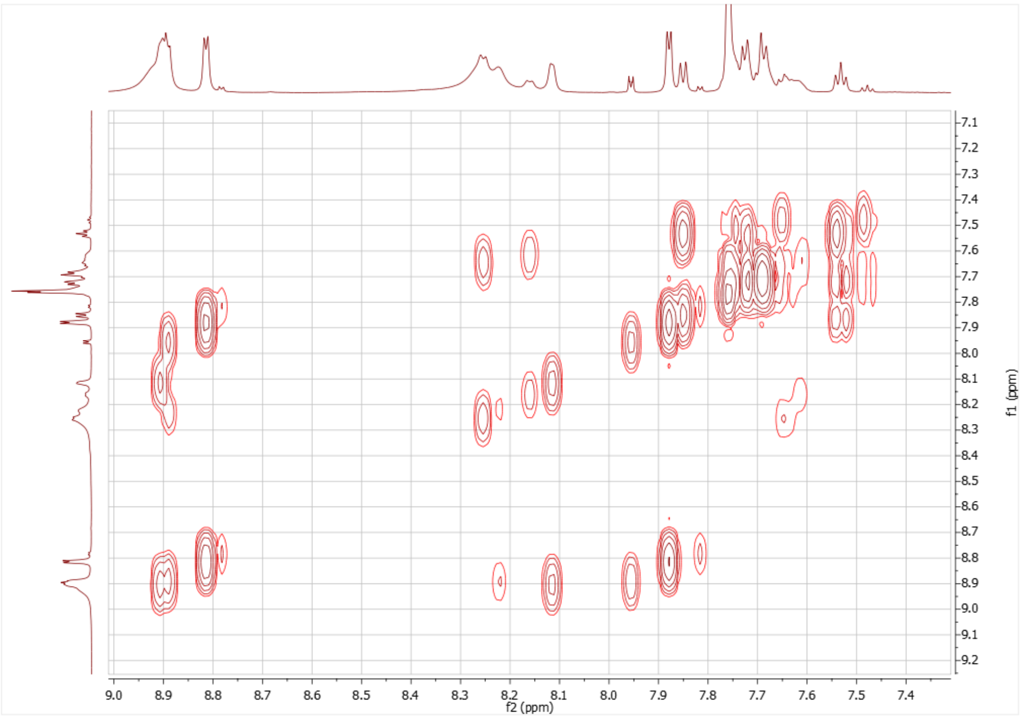

HSQC-NMR in DMSO-d6 700 MHz

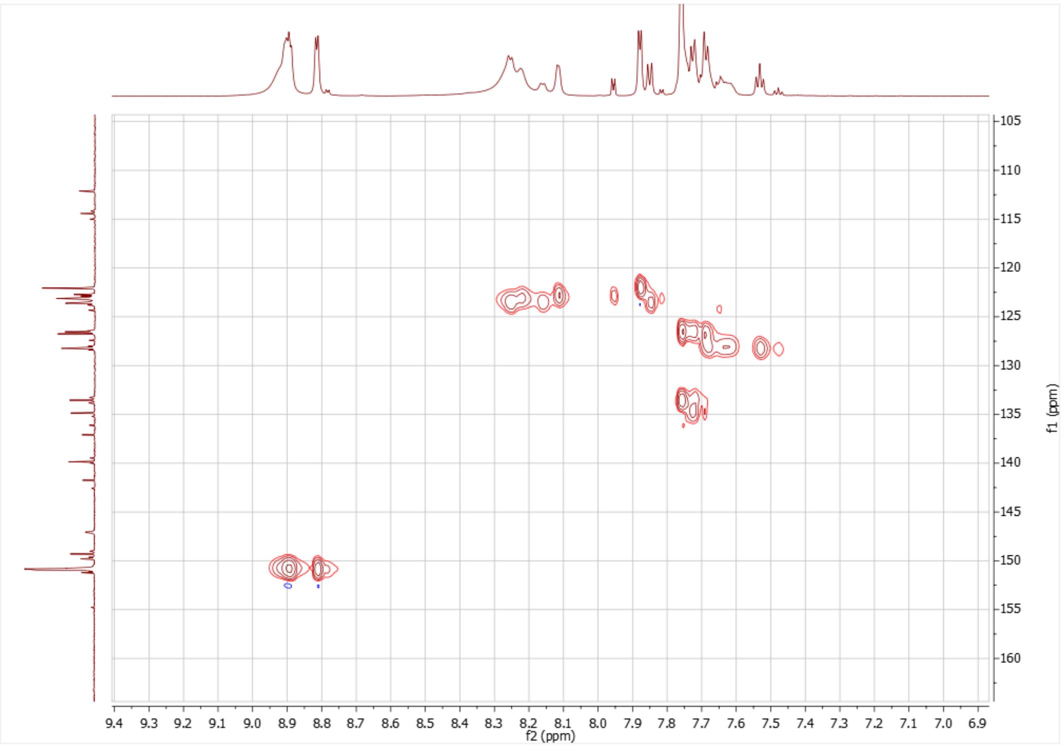

ORTEP of complex 2b:

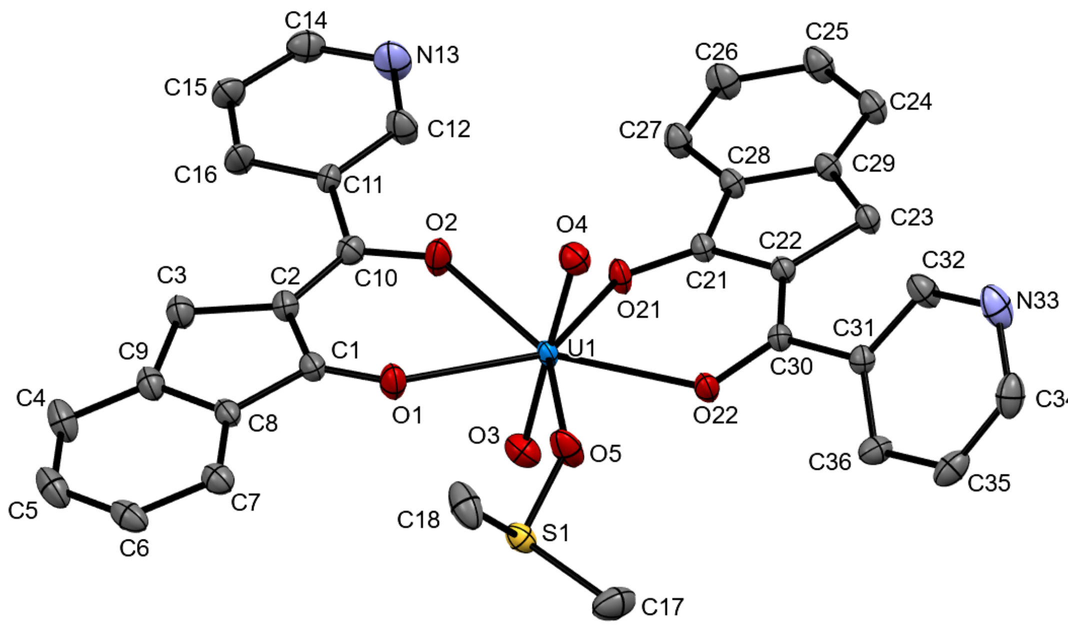

Table. Dates

checkCIF/PLATON report

Structure factors have been supplied for datablock(s) Compound 2b

THIS REPORT IS FOR GUIDANCE ONLY. IF USED AS PART OF A REVIEW PROCEDURE FOR PUBLICATION, IT SHOULD NOT REPLACE THE EXPERTISE OF AN EXPERIENCED CRYSTALLOGRAPHIC REFEREE.

No syntax errors found. CIF dictionary Interpreting this report

Datablock: Compound_2b

Bond precision:	C-C = 0.0023 A	Wavelength=0.71073

Cell:	a=8.9600(4)	b=10.5412(4)	c=16.6705(7)
	alpha=73.863(1)	beta=89.511(1)	gamma=71.335(1)
Temperature:	100 K		

	Calculated	Reported
Volume	1427.33(10)	1427.33(10)
Space group	P -1	P -1
Hall group	-P 1	-P 1

Moiety formula C32 H26 N2 O7 S U C32 H26 N2 O7 S USum formula C32 H26 N2 O7 S U C32 H26 N2 O7 S UMr 820.64 820.64

Dx,g cm-3 1.910 1.909

Z	2	2
Mu (mm-1)	5.813	5.813
F000	792.0	792.0
F000’	773.39	
h,k,lmax	13,15,25	13,15,25
Nref	10394	10383
Tmin,Tmax	0.212,0.302	0.643,0.747
Tmin’	0.135	

Correction method= # Reported T Limits: Tmin=0.643 Tmax=0.747AbsCorr = MULTI-SCAN

Data completeness= 0.999 Theta(max)= 32.575

R(reflections)= 0.0151(10092) wR2(reflections)= 0.0378(10383)

S = 1.173 Npar= 616

The following ALERTS were generated. Each ALERT has the format
test-name_ALERT_alert-type_alert-level.

Click on the hyperlinks for more details of the test.

Alert level C

```
PLAT911_ALERT_3_C Missing FCF Refl Between Thmin & STh/L=    0.600          8 Report
```

Alert level G

```
PLAT002_ALERT_2_G Number of Distance or Angle Restraints on AtSite         60 Note
PLAT003_ALERT_2_G Number of Uiso or Uij Restrained non-H Atoms ...         50 Report
PLAT154_ALERT_1_G The s.u.'s on the Cell Angles are Equal ..(Note)      0.001 Degree
PLAT175_ALERT_4_G The CIF-Embedded .res File Contains SAME Records          3 Report
PLAT177_ALERT_4_G The CIF-Embedded .res File Contains DELU Records          2 Report
PLAT178_ALERT_4_G The CIF-Embedded .res File Contains SIMU Records          2 Report
PLAT187_ALERT_4_G The CIF-Embedded .res File Contains RIGU Records          1 Report
PLAT188_ALERT_3_G A Non-default SIMU Restraint Value has been used     0.0100 Report
PLAT188_ALERT_3_G A Non-default SIMU Restraint Value has been used     0.0100 Report
PLAT189_ALERT_3_G A Non-default SAME Restraint Value for First Par     0.0100 Report
PLAT189_ALERT_3_G A Non-default SAME Restraint Value for SecondPar     0.0100 Report
PLAT189_ALERT_3_G A Non-default SAME Restraint Value for First Par     0.0100 Report
PLAT189_ALERT_3_G A Non-default SAME Restraint Value for SecondPar     0.0100 Report
PLAT189_ALERT_3_G A Non-default SAME Restraint Value for First Par     0.0100 Report
PLAT189_ALERT_3_G A Non-default SAME Restraint Value for SecondPar     0.0100 Report
PLAT192_ALERT_3_G A Non-default DELU Restraint Value for First Par     0.0050 Report
PLAT192_ALERT_3_G A Non-default DELU Restraint Value for First Par     0.0050 Report
PLAT232_ALERT_2_G Hirshfeld Test Diff (M-X)  U1       --O1       .        9.6 s.u.
PLAT232_ALERT_2_G Hirshfeld Test Diff (M-X)  U1       --O2       .        5.7 s.u.
PLAT232_ALERT_2_G Hirshfeld Test Diff (M-X)  U1       --O3       .        8.0 s.u.
PLAT232_ALERT_2_G Hirshfeld Test Diff (M-X)  U1       --O4       .       11.8 s.u.
PLAT232_ALERT_2_G Hirshfeld Test Diff (M-X)  U1       --O5       .        5.7 s.u.
PLAT232_ALERT_2_G Hirshfeld Test Diff (M-X)  U1       --O22      .        5.5 s.u.
PLAT232_ALERT_2_G Hirshfeld Test Diff (M-X)  U1B      --O3       .       36.6 s.u.
PLAT232_ALERT_2_G Hirshfeld Test Diff (M-X)  U1B      --O4       .       38.2 s.u.
PLAT232_ALERT_2_G Hirshfeld Test Diff (M-X)  U1B      --O5       .        9.4 s.u.
PLAT301_ALERT_3_G Main Residue  Disorder ..............(Resd  1  )        58% Note
PLAT410_ALERT_2_G Short Intra H...H Contact  H3B      ..H16      .       2.03 Ang.
                                                      x,y,z  =      1_555 Check
PLAT411_ALERT_2_G Short Inter H...H Contact  H3A      ..H35B     .       2.12 Ang.
                                                 -x,1-y,2-z  =      2_567 Check
PLAT413_ALERT_2_G Short Inter XH3 .. XHn     H17A     ..H24B     .       2.01 Ang.
                                                   x,-1+y,z  =      1_545 Check
PLAT432_ALERT_2_G Short Inter X...Y Contact  C5       ..C24B     .       3.12 Ang.
                                                -1+x,-1+y,z  =      1_445 Check
PLAT432_ALERT_2_G Short Inter X...Y Contact  C18      ..C27B     .       3.19 Ang.
                                                1-x,1-y,1-z  =      2_666 Check
PLAT480_ALERT_4_G Long H...A H-Bond Reported H17A     ..N33      .       2.66 Ang.
PLAT811_ALERT_5_G No ADDSYM Analysis: Too Many Excluded Atoms ....          ! Info
PLAT860_ALERT_3_G Number of Least-Squares Restraints ............        1765 Note
PLAT910_ALERT_3_G Missing # of FCF Reflection(s) Below Theta(Min).          3 Note
PLAT913_ALERT_3_G Missing # of Very Strong Reflections in FCF ....          2 Note
PLAT933_ALERT_2_G Number of HKL-OMIT Records in Embedded .res File          7 Note
PLAT978_ALERT_2_G Number C-C Bonds with Positive Residual Density.         13 Info
```

0 ALERT level A = Most likely a serious problem - resolve or explain

```
 0 ALERT level B = A potentially serious problem, consider carefully
 1 ALERT level C = Check. Ensure it is not caused by an omission or oversight
39 ALERT level G = General information/check it is not something unexpected

 1 ALERT type 1 CIF construction/syntax error, inconsistent or missing data
18 ALERT type 2 Indicator that the structure model may be wrong or deficient
15 ALERT type 3 Indicator that the structure quality may be low
 5 ALERT type 4 Improvement, methodology, query or suggestion
 1 ALERT type 5 Informative message, check
```

It is advisable to attempt to resolve as many as possible of the alerts in all categories. Often the minor alerts point to easily fixed oversights, errors and omissions in your CIF or refinement strategy, so attention to these fine details can be worthwhile. In order to resolve some of the more serious problems it may be necessary to carry out additional measurements or structure refinements. However, the purpose of your study may justify the reported deviations and the more serious of these should normally be commented upon in the discussion or experimental section of a paper or in the "special_details" fields of the CIF. checkCIF was carefully designed to identify outliers and unusual parameters, but every test has its limitations and alerts that are not important in a particular case may appear. Conversely, the absence of alerts does not guarantee there are no aspects of the results needing attention. It is up to the individual to critically assess their own results and, if necessary, seek expert advice.

Publication of your CIF in IUCr journals

A basic structural check has been run on your CIF. These basic checks will be run on all CIFs submitted for publication in IUCr journals (*Acta Crystallographica*, *Journal of Applied Crystallography*, *Journal of Synchrotron Radiation*); however, if you intend to submit to *Acta Crystallographica Section C* or *E* or *IUCrData*, you should make sure that full publication checks are run on the final version of your CIF prior to submission.

Publication of your CIF in other journals

Please refer to the *Notes for Authors* of the relevant journal for any special instructions relating to CIF submission.

PLATON version of 06/07/2023; check.def file version of 30/06/2023

Datablock Compound_2b - ellipsoid plot

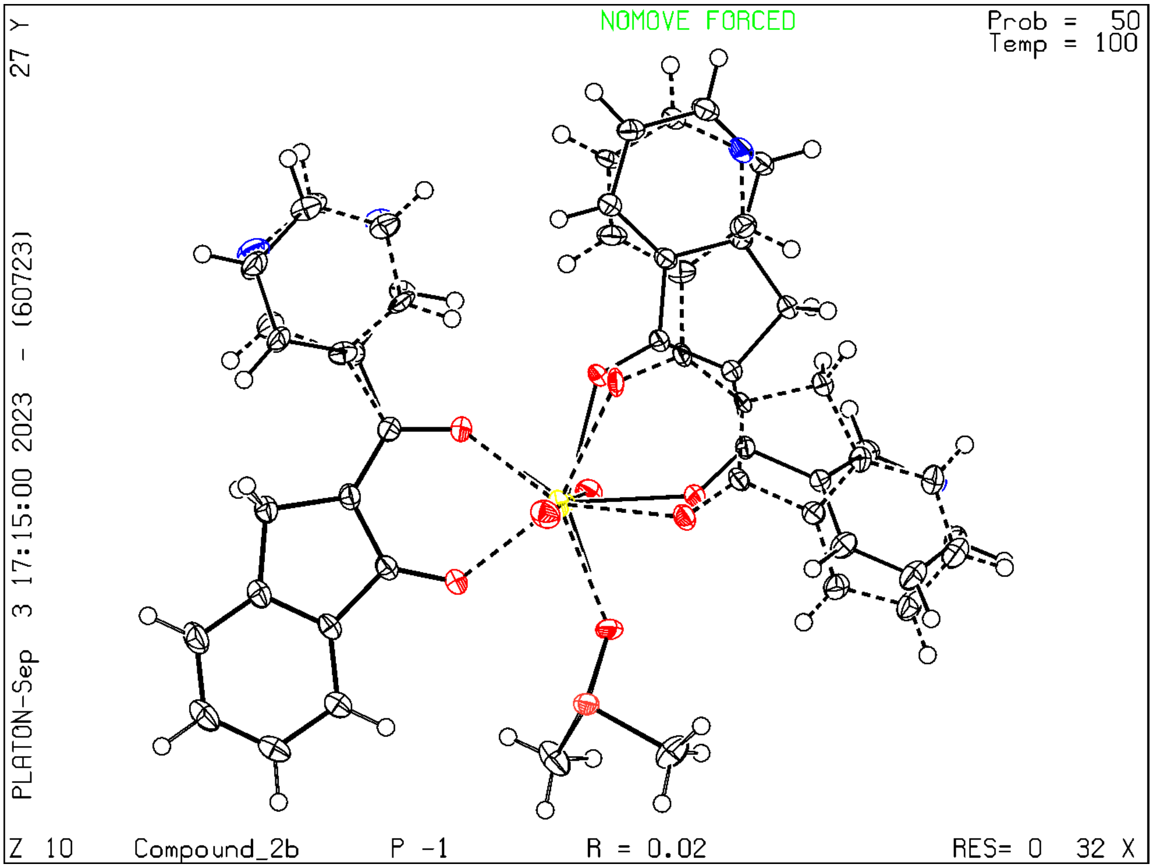

Table. Bond lengths

Number	Object1	Object2	Length
1	O3	U1	1.784(1)
2	U1	O4	1.779(1)
3	U1	O2	2.315(1)
4	U1	O1	2.450(2)
5	U1	O21	2.372(3)
6	U1	O22	2.331(2)
7	U1	O5	2.413(1)
8	C1	O1	1.271(2)
9	O2	C10	1.287(2)
10	C2	C1	1.428(2)
11	C1	C8	1.474(2)
12	C2	C3	1.518(3)

13	C2	C10	1.398(3)
14	C3	H3A	0.99
15	C3	H3B	0.99
16	C3	C9	1.512(3)
17	C4	C5	1.391(3)
18	C4	C9	1.389(3)
19	C5	C6	1.390(3)
20	C6	C7	1.399(3)
21	C7	C8	1.402(2)
22	C8	C9	1.392(2)
23	C10	C11	1.485(4)
24	C11	C16	1.392(5)
25	C12	C11	1.394(7)
26	C12	N13	1.333(7)
27	N13	C14	1.34(1)
28	C14	C15	1.38(1)
29	O21	C21	1.266(3)
30	O22	C30	1.292(3)
31	C21	C22	1.424(3)
32	C22	C30	1.390(2)
33	C21	C28	1.470(3)
34	C28	C27	1.394(3)
35	C27	C26	1.390(3)
36	C26	C25	1.397(3)
37	C25	C24	1.398(3)
38	C24	C29	1.393(4)
39	C29	C23	1.499(4)
40	C23	C22	1.522(3)
41	C30	C31	1.491(2)
42	C31	C32	1.395(2)
43	C32	N33	1.343(3)
44	N33	C34	1.341(3)
45	C34	C35	1.380(3)
46	C35	C36	1.387(3)
47	C36	C31	1.392(2)

Table. Angles

Number	Atom1	Atom2	Atom3	Angle
1	U1	O3	U1B	3.90(9)
2	U1	O4	U1B	3.53(8)
3	C1	O1	U1	130.1(1)
4	C1	O1	U1B	128.3(1)
5	U1	O1	U1B	2.15(7)

6	C10	O2	U1	142.7(1)
7	C10	O2	U1B	139.9(1)
8	U1	O2	U1B	2.91(7)
9	O1	C1	C2	129.3(1)
10	O1	C1	C8	122.7(1)
11	C2	C1	C8	108.0(1)
12	C1	C2	C3	109.0(1)
13	C1	C2	C10	121.6(1)
14	C3	C2	C10	129.4(1)
15	C2	C3	H3A	111
16	C2	C3	H3B	111
17	C2	C3	C9	103.6(1)
18	H3A	C3	H3B	109
19	H3A	C3	C9	111
20	H3B	C3	C9	111
21	H4	C4	C5	120.7
22	H4	C4	C9	120.7
23	C5	C4	C9	118.6(2)
24	C4	C5	H5	119.4
25	C4	C5	C6	121.3(2)
26	H5	C5	C6	119.4
27	C5	C6	H6	119.8
28	C5	C6	C7	120.4(2)
29	H6	C6	C7	119.8
30	C6	C7	H7	120.9
31	C6	C7	C8	118.2(2)
32	H7	C7	C8	120.9
33	C1	C8	C7	129.6(1)
34	C1	C8	C9	109.5(1)
35	C7	C8	C9	120.8(1)
36	C3	C9	C4	129.5(2)
37	C3	C9	C8	109.8(1)
38	C4	C9	C8	120.7(2)
39	O2	C10	C2	120.6(1)
40	O2	C10	C11	113.6(2)
41	O2	C10	C11B	121(1)
42	C2	C10	C11	125.8(2)
43	C2	C10	C11B	118(1)
44	C11	C10	C11B	8(1)
45	C10	C11	C12	118.9(3)
46	C10	C11	C16	123.8(3)
47	C10	C11	C12B	132.2(8)
48	C10	C11	C16B	114(2)
49	C12	C11	C16	117.3(3)
50	C12	C11	C12B	108.8(8)

51	C12	C11	C16B	6(2)
52	C16	C11	C12B	9.4(8)
53	C16	C11	C16B	122(2)
54	C12B	C11	C16B	114(2)
55	C11	C12	H12	117.5
56	C11	C12	N13	125.1(4)
57	C11	C12	C11B	7(1)
58	C11	C12	C15B	127(2)
59	C11	C12	H16B	98.2
60	H12	C12	N13	117.5
61	H12	C12	C11B	124
62	H12	C12	C15B	115
63	H12	C12	H16B	19.5
64	N13	C12	C11B	118(1)
65	N13	C12	C15B	8(2)
66	N13	C12	H16B	136.7
67	C11B	C12	C15B	120(2)
68	C11B	C12	H16B	105
69	C15B	C12	H16B	134
70	C12	H12	C16B	13
71	C12	N13	C14	116.4(5)
72	C12	N13	C14B	120(3)
73	C12	N13	H15B	105.2
74	C12	N13	C16B	4(1)
75	C14	N13	C14B	4(3)
76	C14	N13	H15B	133.2
77	C14	N13	C16B	113(1)
78	C14B	N13	H15B	130
79	C14B	N13	C16B	117(3)
80	H15B	N13	C16B	107
81	N13	C14	H14	118.2
82	N13	C14	C15	123.5(7)
83	N13	C14	N13B	131(1)
84	N13	C14	H14B	103.5
85	N13	C14	C15B	8(1)
86	H14	C14	C15	118.3
87	H14	C14	N13B	110
88	H14	C14	H14B	15.3
89	H14	C14	C15B	122
90	C15	C14	N13B	9(1)
91	C15	C14	H14B	132.8
92	C15	C14	C15B	119(1)
93	N13B	C14	H14B	124
94	N13B	C14	C15B	128(2)
95	H14B	C14	C15B	108

96	C14	H14	C14B	7
97	C14	C15	H15	120.3
98	C14	C15	C16	119.4(5)
99	C14	C15	C12B	121(1)
100	C14	C15	C14B	1(2)
101	H15	C15	C16	120.3
102	H15	C15	C12B	118
103	H15	C15	C14B	121
104	C16	C15	C12B	7(1)
105	C16	C15	C14B	118(2)
106	C12B	C15	C14B	120(2)
107	C15	H15	N13B	16
108	C11	C16	C15	118.3(3)
109	C11	C16	H16	120.9
110	C11	C16	C11B	2(2)
111	C11	C16	H12B	144.1
112	C11	C16	N13B	115.8(9)
113	C15	C16	H16	120.9
114	C15	C16	C11B	120(2)
115	C15	C16	H12B	94.7
116	C15	C16	N13B	4.5(9)
117	H16	C16	C11B	119
118	H16	C16	H12B	29.71
119	H16	C16	N13B	123.2
120	C11B	C16	H12B	143
121	C11B	C16	N13B	118(2)
122	H12B	C16	N13B	96
123	C16	H16	C12B	16
124	O3	U1	O4	178.36(6)
125	O3	U1	O1	85.63(5)
126	O3	U1	O2	94.17(5)
127	O3	U1	O21	89.62(8)
128	O3	U1	O22	92.60(6)
129	O3	U1	O5	94.12(5)
130	O3	U1	O21B	79.6(2)
131	O3	U1	O22B	89.9(4)
132	O4	U1	O1	94.81(5)
133	O4	U1	O2	87.46(5)
134	O4	U1	O21	91.03(7)
135	O4	U1	O22	86.17(6)
136	O4	U1	O5	84.52(5)
137	O4	U1	O21B	99.1(2)
138	O4	U1	O22B	90.5(4)
139	O1	U1	O2	69.65(5)
140	O1	U1	O21	140.71(7)

141	O1	U1	O22	147.41(5)
142	O1	U1	O5	72.01(5)
143	O1	U1	O21B	136.8(2)
144	O1	U1	O22B	148.9(4)
145	O2	U1	O21	71.85(7)
146	O2	U1	O22	142.82(5)
147	O2	U1	O5	139.93(5)
148	O2	U1	O21B	151.1(2)
149	O2	U1	O22B	80.1(4)
150	O21	U1	O22	71.68(7)
151	O21	U1	O5	147.28(7)
152	O21	U1	O21B	79.9(2)
153	O21	U1	O22B	8.3(4)
154	O22	U1	O5	75.68(5)
155	O22	U1	O21B	15.4(2)
156	O22	U1	O22B	63.4(4)
157	O5	U1	O21B	68.9(2)
158	O5	U1	O22B	139.0(4)
159	O21B	U1	O22B	71.8(5)
160	U1	O21	C21	130.4(2)
161	U1	O21	U1B	3.13(7)
162	U1	O21	C30B	115.3(4)
163	C21	O21	U1B	133.5(2)
164	C21	O21	C30B	15.8(4)
165	U1B	O21	C30B	118.4(4)
166	U1	O22	C30	132.4(1)
167	U1	O22	U1B	1.70(6)
168	U1	O22	O21B	78.7(8)
169	U1	O22	C21B	160.2(6)
170	C30	O22	U1B	133.6(1)
171	C30	O22	O21B	144.2(8)
172	C30	O22	C21B	27.8(6)
173	U1B	O22	O21B	77.1(8)
174	U1B	O22	C21B	161.3(6)
175	O21B	O22	C21B	119(1)
176	O21	C21	C22	128.5(2)
177	O21	C21	C28	123.3(2)
178	O21	C21	O22B	14.5(9)
179	O21	C21	C31B	137.3(7)
180	C22	C21	C28	108.1(2)
181	C22	C21	O22B	114.9(9)
182	C22	C21	C31B	94.1(7)
183	C28	C21	O22B	136.5(9)
184	C28	C21	C31B	14.3(7)
185	O22B	C21	C31B	151(1)

186	C21	C22	C23	109.3(1)
187	C21	C22	C30	121.5(2)
188	C21	C22	C22B	130.2(9)
189	C21	C22	C30B	5.2(7)
190	C23	C22	C30	128.7(2)
191	C23	C22	C22B	119.4(9)
192	C23	C22	C30B	113.9(7)
193	C30	C22	C22B	9.6(9)
194	C30	C22	C30B	116.5(7)
195	C22B	C22	C30B	125(1)
196	C22	C23	H23A	111.2
197	C22	C23	H23B	111.2
198	C22	C23	C29	103.0(2)
199	C22	C23	C23B	67.1(4)
200	C22	C23	H23D	94.3
201	C22	C23	C32B	109(1)
202	C22	C23	H32B	140
203	H23A	C23	H23B	109.1
204	H23A	C23	C29	111.2
205	H23A	C23	C23B	76.9
206	H23A	C23	H23D	38.11
207	H23A	C23	C32B	108
208	H23A	C23	H32B	86.4
209	H23B	C23	C29	111.2
210	H23B	C23	C23B	70.9
211	H23B	C23	H23D	85.3
212	H23B	C23	C32B	108
213	H23B	C23	H32B	95
214	C29	C23	C23B	169.5(4)
215	C29	C23	H23D	149.2
216	C29	C23	C32B	6(1)
217	C29	C23	H32B	37.6
218	C23B	C23	H23D	39.6
219	C23B	C23	C32B	175(1)
220	C23B	C23	H32B	152.7
221	H23D	C23	C32B	146
222	H23D	C23	H32B	118.4
223	C32B	C23	H32B	32
224	H24	C24	C25	121
225	H24	C24	C29	121
226	H24	C24	C32B	122
227	H24	C24	C34B	137.5
228	C25	C24	C29	118.0(2)
229	C25	C24	C32B	117(1)
230	C25	C24	C34B	16.8(4)

231	C29	C24	C32B	1(1)
232	C29	C24	C34B	101.5(4)
233	C32B	C24	C34B	101(1)
234	C24	H24	N33B	14.8
235	C24	C25	H25	119.3
236	C24	C25	C26	121.4(2)
237	C24	C25	N33B	4.7(8)
238	C24	C25	C34B	120(1)
239	C24	C25	H34B	169.7
240	H25	C25	C26	119.3
241	H25	C25	N33B	124.1
242	H25	C25	C34B	120
243	H25	C25	H34B	52.7
244	C26	C25	N33B	116.7(8)
245	C26	C25	C34B	9(1)
246	C26	C25	H34B	67
247	N33B	C25	C34B	115(1)
248	N33B	C25	H34B	172.7
249	C34B	C25	H34B	67
250	C25	H25	C34B	22.5
251	C25	C26	H26	119.6
252	C25	C26	C27	120.8(2)
253	C25	C26	C34B	6.6(9)
254	C25	C26	C35B	139(1)
255	C25	C26	H35B	165.6
256	H26	C26	C27	119.6
257	H26	C26	C34B	120
258	H26	C26	C35B	99
259	H26	C26	H35B	49.99
260	C27	C26	C34B	119.9(9)
261	C27	C26	C35B	24(1)
262	C27	C26	H35B	70.6
263	C34B	C26	C35B	136(1)
264	C34B	C26	H35B	161.1
265	C35B	C26	H35B	49
266	C26	H26	C35B	32
267	C26	C27	H27	121.2
268	C26	C27	C28	117.6(2)
269	C26	C27	C31B	116.7(5)
270	C26	C27	C35B	19.5(8)
271	C26	C27	C36B	151(1)
272	C26	C27	H36B	158.1
273	H27	C27	C28	121.2
274	H27	C27	C31B	122.1
275	H27	C27	C35B	104.7

276	H27	C27	C36B	82
277	H27	C27	H36B	41.47
278	C28	C27	C31B	2.5(5)
279	C28	C27	C35B	132.8(8)
280	C28	C27	C36B	43(1)
281	C28	C27	H36B	81.3
282	C31B	C27	C35B	131(1)
283	C31B	C27	C36B	43(1)
284	C31B	C27	H36B	81.7
285	C35B	C27	C36B	150(1)
286	C35B	C27	H36B	138.6
287	C36B	C27	H36B	41
288	C27	H27	C36B	33.4
289	C21	C28	C27	129.1(2)
290	C21	C28	C29	108.8(2)
291	C21	C28	C30B	14.4(4)
292	C21	C28	C32B	117(1)
293	C21	C28	C36B	108.9(7)
294	C27	C28	C29	122.0(2)
295	C27	C28	C30B	143.5(4)
296	C27	C28	C32B	114(1)
297	C27	C28	C36B	22.1(6)
298	C29	C28	C30B	94.5(4)
299	C29	C28	C32B	8(1)
300	C29	C28	C36B	140.7(7)
301	C30B	C28	C32B	102(1)
302	C30B	C28	C36B	123.1(8)
303	C32B	C28	C36B	133(1)
304	C23	C29	C24	129.2(3)
305	C23	C29	C28	110.7(2)
306	C23	C29	C31B	96.0(7)
307	C23	C29	H32B	43.4
308	C23	C29	N33B	144.4(6)
309	C24	C29	C28	120.1(3)
310	C24	C29	C31B	134.6(7)
311	C24	C29	H32B	86.2
312	C24	C29	N33B	15.3(5)
313	C28	C29	C31B	14.9(7)
314	C28	C29	H32B	153.3
315	C28	C29	N33B	104.8(5)
316	C31B	C29	H32B	139.2
317	C31B	C29	N33B	119.3(9)
318	H32B	C29	N33B	101.5
319	O22	C30	C22	123.1(2)
320	O22	C30	C31	114.7(2)

321	O22	C30	C21B	35.8(8)
322	O22	C30	C22B	129.3(7)
323	C22	C30	C31	122.2(2)
324	C22	C30	C21B	145.4(8)
325	C22	C30	C22B	7.0(7)
326	C31	C30	C21B	85.3(8)
327	C31	C30	C22B	115.8(7)
328	C21B	C30	C22B	148(1)
329	C30	C31	C32	121.9(2)
330	C30	C31	C36	120.3(2)
331	C30	C31	C23B	69.5(3)
332	C30	C31	C28B	93.6(8)
333	C30	C31	C29B	129.8(7)
334	C32	C31	C36	117.6(2)
335	C32	C31	C23B	63.4(3)
336	C32	C31	C28B	142.7(8)
337	C32	C31	C29B	34.0(7)
338	C36	C31	C23B	149.8(3)
339	C36	C31	C28B	27.4(7)
340	C36	C31	C29B	102.4(7)
341	C23B	C31	C28B	150.3(8)
342	C23B	C31	C29B	60.4(8)
343	C28B	C31	C29B	129(1)
344	C31	C32	H32	117.9
345	C31	C32	N33	124.2(2)
346	C31	C32	C23B	68.1(3)
347	C31	C32	C24B	98.7(5)
348	C31	C32	C29B	33.0(7)
349	H32	C32	N33	117.9
350	H32	C32	C23B	61.2
351	H32	C32	C24B	137.1
352	H32	C32	C29B	125.3
353	N33	C32	C23B	145.5(4)
354	N33	C32	C24B	33.2(5)
355	N33	C32	C29B	107.3(7)
356	C23B	C32	C24B	121.1(6)
357	C23B	C32	C29B	64.2(7)
358	C24B	C32	C29B	75.0(8)
359	C32	N33	C34	116.4(2)
360	C32	N33	C24B	73.9(9)
361	C32	N33	H24B	100
362	C32	N33	C25B	110.8(6)
363	C34	N33	C24B	60.5(9)
364	C34	N33	H24B	100.3
365	C34	N33	C25B	6.0(6)

366	C24B	N33	H24B	67.1
367	C24B	N33	C25B	55(1)
368	H24B	N33	C25B	99.6
369	N33	C34	H34	118.1
370	N33	C34	C35	123.9(2)
371	N33	C34	C24B	34.8(6)
372	N33	C34	H25B	132.1
373	N33	C34	C26B	132.9(5)
374	H34	C34	C35	118
375	H34	C34	C24B	133
376	H34	C34	H25B	15.17
377	H34	C34	C26B	107
378	C35	C34	C24B	100.4(6)
379	C35	C34	H25B	103.7
380	C35	C34	C26B	17.2(4)
381	C24B	C34	H25B	138.9
382	C24B	C34	C26B	115.8(7)
383	H25B	C34	C26B	94.4
384	C34	H34	C25B	22
385	C34	C35	H35	120.6
386	C34	C35	C36	118.7(2)
387	C34	C35	C25B	10.6(9)
388	C34	C35	C26B	115(1)
389	C34	C35	C27B	138.2(4)
390	H35	C35	C36	120.6
391	H35	C35	C25B	110.8
392	H35	C35	C26B	45
393	H35	C35	C27B	96.3
394	C36	C35	C25B	128.3(9)
395	C36	C35	C26B	107(1)
396	C36	C35	C27B	30.9(4)
397	C25B	C35	C26B	112(2)
398	C25B	C35	C27B	149(1)
399	C26B	C35	C27B	77(1)
400	C35	H35	C26B	35
401	C31	C36	C35	118.9(2)
402	C31	C36	H36	120.5
403	C31	C36	C27B	139.2(8)
404	C31	C36	C28B	23.3(6)
405	C31	C36	C29B	27.2(3)
406	C35	C36	H36	120.5
407	C35	C36	C27B	76.8(8)
408	C35	C36	C28B	141.1(7)
409	C35	C36	C29B	94.6(3)
410	H36	C36	C27B	58.7

411	H36	C36	C28B	97.9
412	H36	C36	C29B	142.9
413	C27B	C36	C28B	125(1)
414	C27B	C36	C29B	151.0(8)
415	C28B	C36	C29B	50.2(7)
416	C36	H36	C27B	48.7
417	C36	H36	C28B	37.6
418	C27B	H36	C28B	75.3
419	O5	S1	C17	104.44(9)
420	O5	S1	C18	104.43(9)
421	C17	S1	C18	97.4(1)
422	U1	O5	S1	133.37(8)
423	U1	O5	U1B	3.23(6)
424	S1	O5	U1B	130.4(1)
425	S1	C17	H17A	109.5
426	S1	C17	H17B	109.5
427	S1	C17	H17C	109.5
428	H17A	C17	H17B	109.5
429	H17A	C17	H17C	109.5
430	H17B	C17	H17C	109.5
431	S1	C18	H18A	109.5
432	S1	C18	H18B	109.5
433	S1	C18	H18C	109.5
434	H18A	C18	H18B	109.5
435	H18A	C18	H18C	109.5
436	H18B	C18	H18C	109.4
437	C10	C11B	C12	105(2)
438	C10	C11B	C16	133(2)
439	C10	C11B	C12B	142(2)
440	C10	C11B	C16B	100(2)
441	C12	C11B	C16	122(2)
442	C12	C11B	C12B	112(2)
443	C12	C11B	C16B	6(1)
444	C16	C11B	C12B	11(1)
445	C16	C11B	C16B	127(3)
446	C12B	C11B	C16B	118(3)
447	C11	C12B	C15	123(2)
448	C11	C12B	H16	97
449	C11	C12B	C11B	3(1)
450	C11	C12B	H12B	121
451	C11	C12B	N13B	121(2)
452	C15	C12B	H16	136
453	C15	C12B	C11B	125(2)
454	C15	C12B	H12B	116
455	C15	C12B	N13B	6(1)

456	H16	C12B	C11B	94
457	H16	C12B	H12B	26.4
458	H16	C12B	N13B	140
459	C11B	C12B	H12B	118
460	C11B	C12B	N13B	124(2)
461	H12B	C12B	N13B	118
462	C16	H12B	C12B	19
463	C14	N13B	H15	142
464	C14	N13B	C16	113(2)
465	C14	N13B	C12B	116(2)
466	C14	N13B	C14B	1(3)
467	H15	N13B	C16	102
468	H15	N13B	C12B	100
469	H15	N13B	C14B	142
470	C16	N13B	C12B	5(1)
471	C16	N13B	C14B	113(3)
472	C12B	N13B	C14B	116(3)
473	N13	C14B	H14	129
474	N13	C14B	C15	120(4)
475	N13	C14B	N13B	128(5)
476	N13	C14B	H14B	114
477	N13	C14B	C15B	8(1)
478	H14	C14B	C15	110
479	H14	C14B	N13B	103
480	H14	C14B	H14B	16
481	H14	C14B	C15B	133
482	C15	C14B	N13B	9(1)
483	C15	C14B	H14B	126
484	C15	C14B	C15B	116(4)
485	N13B	C14B	H14B	118
486	N13B	C14B	C15B	124(5)
487	H14B	C14B	C15B	118
488	C14	H14B	C14B	6
489	C12	C15B	C14	117(3)
490	C12	C15B	C14B	121(4)
491	C12	C15B	H15B	118
492	C12	C15B	C16B	5(1)
493	C14	C15B	C14B	4(2)
494	C14	C15B	H15B	125
495	C14	C15B	C16B	115(3)
496	C14B	C15B	H15B	121
497	C14B	C15B	C16B	119(4)
498	H15B	C15B	C16B	120
499	N13	H15B	C15B	13
500	C11	C16B	H12	134

501	C11	C16B	N13	123(3)
502	C11	C16B	C11B	7(1)
503	C11	C16B	C15B	125(3)
504	C11	C16B	H16B	114
505	H12	C16B	N13	102
506	H12	C16B	C11B	141
507	H12	C16B	C15B	101
508	H12	C16B	H16B	20.2
509	N13	C16B	C11B	116(3)
510	N13	C16B	C15B	7(1)
511	N13	C16B	H16B	122
512	C11B	C16B	C15B	118(3)
513	C11B	C16B	H16B	121
514	C15B	C16B	H16B	121
515	C12	H16B	C16B	12
516	O3	U1B	O4	171.4(2)
517	O3	U1B	O1	90.7(1)
518	O3	U1B	O2	99.1(1)
519	O3	U1B	O21	89.8(1)
520	O3	U1B	O22	90.4(1)
521	O3	U1B	O5	95.1(1)
522	O3	U1B	O21B	78.1(2)
523	O3	U1B	O22B	89.6(4)
524	O4	U1B	O1	96.2(1)
525	O4	U1B	O2	87.9(1)
526	O4	U1B	O21	87.6(1)
527	O4	U1B	O22	81.0(1)
528	O4	U1B	O5	82.0(1)
529	O4	U1B	O21B	93.3(2)
530	O4	U1B	O22B	86.6(4)
531	O1	U1B	O2	72.93(9)
532	O1	U1B	O21	144.8(1)
533	O1	U1B	O22	146.3(1)
534	O1	U1B	O5	73.35(9)
535	O1	U1B	O21B	137.3(2)
536	O1	U1B	O22B	152.9(4)
537	O2	U1B	O21	72.3(1)
538	O2	U1B	O22	139.8(1)
539	O2	U1B	O5	143.4(1)
540	O2	U1B	O21B	149.2(2)
541	O2	U1B	O22B	80.3(4)
542	O21	U1B	O22	68.8(1)
543	O21	U1B	O5	141.6(1)
544	O21	U1B	O21B	77.0(2)
545	O21	U1B	O22B	8.1(4)

546	O22	U1B	O5	73.02(9)
547	O22	U1B	O21B	14.6(2)
548	O22	U1B	O22B	60.8(4)
549	O5	U1B	O21B	66.9(2)
550	O5	U1B	O22B	133.6(4)
551	O21B	U1B	O22B	69.1(4)
552	U1	O21B	O22	86.0(8)
553	U1	O21B	U1B	2.40(7)
554	U1	O21B	C21B	121.0(7)
555	O22	O21B	U1B	88.2(8)
556	O22	O21B	C21B	35.5(7)
557	U1B	O21B	C21B	123.2(7)
558	U1	O22B	C21	147(1)
559	U1	O22B	U1B	2.98(7)
560	U1	O22B	C30B	130(1)
561	C21	O22B	U1B	150(1)
562	C21	O22B	C30B	21.0(6)
563	U1B	O22B	C30B	133(1)
564	O22	C21B	C30	116(1)
565	O22	C21B	O21B	25.3(5)
566	O22	C21B	C22B	112.3(9)
567	O22	C21B	C28B	135(1)
568	C30	C21B	O21B	139(1)
569	C30	C21B	C22B	17.8(6)
570	C30	C21B	C28B	96(1)
571	O21B	C21B	C22B	130.0(9)
572	O21B	C21B	C28B	122.2(9)
573	C22B	C21B	C28B	107.8(8)
574	C22	C22B	C30	163(2)
575	C22	C22B	C21B	156(1)
576	C22	C22B	C23B	95(1)
577	C22	C22B	C30B	34.2(8)
578	C30	C22B	C21B	14.7(5)
579	C30	C22B	C23B	99.8(9)
580	C30	C22B	C30B	131(1)
581	C21B	C22B	C23B	109.0(8)
582	C21B	C22B	C30B	121.6(9)
583	C23B	C22B	C30B	129.2(9)
584	C23	C23B	C31	150.0(6)
585	C23	C23B	C32	158.5(7)
586	C23	C23B	C22B	76.3(6)
587	C23	C23B	H23C	78.1
588	C23	C23B	H23D	60.6
589	C23	C23B	C29B	169.9(8)
590	C31	C23B	C32	48.5(3)

591	C31	C23B	C22B	74.7(5)
592	C31	C23B	H23C	119.9
593	C31	C23B	H23D	124.6
594	C31	C23B	C29B	28.5(4)
595	C32	C23B	C22B	115.4(7)
596	C32	C23B	H23C	80.7
597	C32	C23B	H23D	124.5
598	C32	C23B	C29B	30.5(4)
599	C22B	C23B	H23C	111.1
600	C22B	C23B	H23D	111.1
601	C22B	C23B	C29B	103.1(8)
602	H23C	C23B	H23D	109.1
603	H23C	C23B	C29B	111.2
604	H23D	C23B	C29B	111.1
605	C23	H23D	C23B	79.8
606	C32	C24B	N33	72.9(8)
607	C32	C24B	C34	129.3(9)
608	C32	C24B	H24B	98.5
609	C32	C24B	C25B	131(1)
610	C32	C24B	C29B	35.9(5)
611	N33	C24B	C34	85(1)
612	N33	C24B	H24B	64.6
613	N33	C24B	C25B	98(1)
614	N33	C24B	C29B	108(1)
615	C34	C24B	H24B	112
616	C34	C24B	C25B	13.5(7)
617	C34	C24B	C29B	126(1)
618	H24B	C24B	C25B	121
619	H24B	C24B	C29B	121
620	C25B	C24B	C29B	118(1)
621	N33	H24B	C24B	48.3
622	N33	C25B	H34	91
623	N33	C25B	C35	119(1)
624	N33	C25B	C24B	27.0(6)
625	N33	C25B	H25B	105
626	N33	C25B	C26B	130(1)
627	H34	C25B	C35	148
628	H34	C25B	C24B	107
629	H34	C25B	H25B	14.9
630	H34	C25B	C26B	129
631	C35	C25B	C24B	104(1)
632	C35	C25B	H25B	135
633	C35	C25B	C26B	21.6(6)
634	C24B	C25B	H25B	119
635	C24B	C25B	C26B	123(1)

636	H25B	C25B	C26B	119
637	C34	H25B	C25B	22
638	C34	C26B	C35	48(1)
639	C34	C26B	H35	111
640	C34	C26B	C25B	8.3(7)
641	C34	C26B	H26B	124
642	C34	C26B	C27B	115.6(9)
643	C35	C26B	H35	100
644	C35	C26B	C25B	47(1)
645	C35	C26B	H26B	148
646	C35	C26B	C27B	81(1)
647	H35	C26B	C25B	103
648	H35	C26B	H26B	50.5
649	H35	C26B	C27B	115
650	C25B	C26B	H26B	120
651	C25B	C26B	C27B	120(1)
652	H26B	C26B	C27B	120
653	C35	C27B	C36	72.3(8)
654	C35	C27B	H36	126
655	C35	C27B	C26B	22.5(5)
656	C35	C27B	H27B	141.2
657	C35	C27B	C28B	96.4(7)
658	C36	C27B	H36	72.6
659	C36	C27B	C26B	95(1)
660	C36	C27B	H27B	139
661	C36	C27B	C28B	29.3(6)
662	H36	C27B	C26B	140
663	H36	C27B	H27B	67.5
664	H36	C27B	C28B	68.6
665	C26B	C27B	H27B	121
666	C26B	C27B	C28B	117.1(9)
667	H27B	C27B	C28B	121.4
668	C31	C28B	C36	129(1)
669	C31	C28B	H36	168
670	C31	C28B	C21B	84.9(9)
671	C31	C28B	C27B	145(1)
672	C31	C28B	C29B	27.5(6)
673	C36	C28B	H36	44.4
674	C36	C28B	C21B	144(1)
675	C36	C28B	C27B	26.2(5)
676	C36	C28B	C29B	102.6(9)
677	H36	C28B	C21B	100.1
678	H36	C28B	C27B	36.1
679	H36	C28B	C29B	142.4
680	C21B	C28B	C27B	127.9(8)

681	C21B	C28B	C29B	109.2(8)
682	C27B	C28B	C29B	122.9(9)
683	C31	C29B	C32	113(1)
684	C31	C29B	C36	50.4(5)
685	C31	C29B	C23B	91.1(9)
686	C31	C29B	C24B	138(1)
687	C31	C29B	C28B	23.5(5)
688	C32	C29B	C36	128.2(9)
689	C32	C29B	C23B	85.4(8)
690	C32	C29B	C24B	69.1(8)
691	C32	C29B	C28B	126(1)
692	C36	C29B	C23B	134.4(7)
693	C36	C29B	C24B	93.7(7)
694	C36	C29B	C28B	27.3(4)
695	C23B	C29B	C24B	130.1(9)
696	C23B	C29B	C28B	110.2(8)
697	C24B	C29B	C28B	119.5(9)
698	O21	C30B	C22	149(1)
699	O21	C30B	C28	93.3(6)
700	O21	C30B	O22B	9.3(7)
701	O21	C30B	C22B	128.8(9)
702	O21	C30B	C31B	102.9(9)
703	C22	C30B	C28	117.7(9)
704	C22	C30B	O22B	141(1)
705	C22	C30B	C22B	20.7(5)
706	C22	C30B	C31B	108(1)
707	C28	C30B	O22B	102(1)
708	C28	C30B	C22B	137.9(9)
709	C28	C30B	C31B	10.0(6)
710	O22B	C30B	C22B	120(1)
711	O22B	C30B	C31B	111(1)
712	C22B	C30B	C31B	128(1)
713	C21	C31B	C27	114(1)
714	C21	C31B	C29	137(1)
715	C21	C31B	C30B	18.7(5)
716	C21	C31B	C32B	144(2)
717	C21	C31B	C36B	100(1)
718	C27	C31B	C29	108(1)
719	C27	C31B	C30B	133(1)
720	C27	C31B	C32B	101(1)
721	C27	C31B	C36B	16.2(5)
722	C29	C31B	C30B	118(1)
723	C29	C31B	C32B	7(1)
724	C29	C31B	C36B	123(1)
725	C30B	C31B	C32B	126(2)

726	C30B	C31B	C36B	118(1)
727	C32B	C31B	C36B	116(2)
728	C23	C32B	C24	134(2)
729	C23	C32B	C28	97(2)
730	C23	C32B	C31B	83(2)
731	C23	C32B	H32B	35
732	C23	C32B	N33B	152(2)
733	C24	C32B	C28	129(2)
734	C24	C32B	C31B	143(3)
735	C24	C32B	H32B	99
736	C24	C32B	N33B	18.0(8)
737	C28	C32B	C31B	14.3(7)
738	C28	C32B	H32B	132
739	C28	C32B	N33B	111(2)
740	C31B	C32B	H32B	118
741	C31B	C32B	N33B	125(2)
742	H32B	C32B	N33B	117
743	C23	H32B	C29	99
744	C23	H32B	C32B	113
745	C29	H32B	C32B	14
746	H24	N33B	C25	131
747	H24	N33B	C29	90.9
748	H24	N33B	C32B	89
749	H24	N33B	C34B	154
750	C25	N33B	C29	138(1)
751	C25	N33B	C32B	140(2)
752	C25	N33B	C34B	23.6(7)
753	C29	N33B	C32B	2(1)
754	C29	N33B	C34B	115(1)
755	C32B	N33B	C34B	117(2)
756	C24	C34B	C25	43(1)
757	C24	C34B	H25	80.4
758	C24	C34B	C26	138(1)
759	C24	C34B	N33B	2.1(6)
760	C24	C34B	H34B	120
761	C24	C34B	C35B	121.2(9)
762	C25	C34B	H25	37.8
763	C25	C34B	C26	164(2)
764	C25	C34B	N33B	41(1)
765	C25	C34B	H34B	78
766	C25	C34B	C35B	163(2)
767	H25	C34B	C26	140
768	H25	C34B	N33B	78.4
769	H25	C34B	H34B	40
770	H25	C34B	C35B	158

771	C26	C34B	N33B	139(1)
772	C26	C34B	H34B	101
773	C26	C34B	C35B	19.8(7)
774	N33B	C34B	H34B	118
775	N33B	C34B	C35B	123(1)
776	H34B	C34B	C35B	118
777	C25	H34B	C34B	35.1
778	C26	C35B	H26	48.9
779	C26	C35B	C27	136(2)
780	C26	C35B	C34B	24.5(8)
781	C26	C35B	H35B	99
782	C26	C35B	C36B	139(1)
783	H26	C35B	C27	157
784	H26	C35B	C34B	71.3
785	H26	C35B	H35B	49.9
786	H26	C35B	C36B	168
787	C27	C35B	C34B	120(1)
788	C27	C35B	H35B	118
789	C27	C35B	C36B	12.1(6)
790	C34B	C35B	H35B	121
791	C34B	C35B	C36B	119(1)
792	H35B	C35B	C36B	121
793	C26	H35B	C35B	32.1
794	C27	C36B	H27	65
795	C27	C36B	C28	115(2)
796	C27	C36B	C31B	121(2)
797	C27	C36B	C35B	17.5(9)
798	C27	C36B	H36B	116
799	H27	C36B	C28	159
800	H27	C36B	C31B	162
801	H27	C36B	C35B	70.5
802	H27	C36B	H36B	51.6
803	C28	C36B	C31B	6.9(6)
804	C28	C36B	C35B	116(1)
805	C28	C36B	H36B	124
806	C31B	C36B	C35B	121(1)
807	C31B	C36B	H36B	120
808	C35B	C36B	H36B	120
809	C27	H36B	C36B	23.3

Table. Torsions

Number	Atom1	Atom2	Atom3	Atom4	Torsion
1	U1B	O3	U1	O4	133(3)

2	U1B	O3	U1	O1	28(1)
3	U1B	O3	U1	O2	-41(1)
4	U1B	O3	U1	O21	-113(1)
5	U1B	O3	U1	O22	175(1)
6	U1B	O3	U1	O5	99(1)
7	U1B	O3	U1	O21B	167(1)
8	U1B	O3	U1	O22B	-121(1)
9	U1	O3	U1B	O4	-7.6(5)
10	U1	O3	U1B	O1	-151(1)
11	U1	O3	U1B	O2	136(1)
12	U1	O3	U1B	O21	64(1)
13	U1	O3	U1B	O22	-5(1)
14	U1	O3	U1B	O5	-78(1)
15	U1	O3	U1B	O21B	-12(1)
16	U1	O3	U1B	O22B	56(1)
17	U1B	O4	U1	O3	-133(3)
18	U1B	O4	U1	O1	-27(1)
19	U1B	O4	U1	O2	42(1)
20	U1B	O4	U1	O21	114(1)
21	U1B	O4	U1	O22	-174(1)
22	U1B	O4	U1	O5	-98(1)
23	U1B	O4	U1	O21B	-166(1)
24	U1B	O4	U1	O22B	122(1)
25	U1	O4	U1B	O3	8.4(5)
26	U1	O4	U1B	O1	151(1)
27	U1	O4	U1B	O2	-136(1)
28	U1	O4	U1B	O21	-64(1)
29	U1	O4	U1B	O22	5(1)
30	U1	O4	U1B	O5	79(1)
31	U1	O4	U1B	O21B	13(1)
32	U1	O4	U1B	O22B	-56(1)
33	U1	O1	C1	C2	-20.2(2)
34	U1	O1	C1	C8	160.8(1)
35	U1B	O1	C1	C2	-21.8(3)
36	U1B	O1	C1	C8	159.2(1)
37	C1	O1	U1	O3	-72.4(1)
38	C1	O1	U1	O4	109.2(1)
39	C1	O1	U1	O2	23.7(1)
40	C1	O1	U1	O21	11.6(2)
41	C1	O1	U1	O22	-160.4(1)
42	C1	O1	U1	O5	-168.2(1)
43	C1	O1	U1	O21B	-142.1(3)

44	C1	O1	U1	O22B	10.1(8)
45	U1B	O1	U1	O3	-38(2)
46	U1B	O1	U1	O4	144(2)
47	U1B	O1	U1	O2	58(2)
48	U1B	O1	U1	O21	46(2)
49	U1B	O1	U1	O22	-126(2)
50	U1B	O1	U1	O5	-134(2)
51	U1B	O1	U1	O21B	-108(2)
52	U1B	O1	U1	O22B	45(2)
53	C1	O1	U1B	O3	-73.3(2)
54	C1	O1	U1B	O4	111.9(2)
55	C1	O1	U1B	O2	26.1(2)
56	C1	O1	U1B	O21	17.4(3)
57	C1	O1	U1B	O22	-165.1(2)
58	C1	O1	U1B	O5	-168.3(1)
59	C1	O1	U1B	O21B	-146.3(4)
60	C1	O1	U1B	O22B	17.2(9)
61	U1	O1	U1B	O3	140(2)
62	U1	O1	U1B	O4	-35(2)
63	U1	O1	U1B	O2	-120(2)
64	U1	O1	U1B	O21	-129(2)
65	U1	O1	U1B	O22	48(2)
66	U1	O1	U1B	O5	45(2)
67	U1	O1	U1B	O21B	67(2)
68	U1	O1	U1B	O22B	-129(2)
69	U1	O2	C10	C2	17.5(3)
70	U1	O2	C10	C11	-164.0(2)
71	U1	O2	C10	C11B	-163(1)
72	U1B	O2	C10	C2	18.7(3)
73	U1B	O2	C10	C11	-162.8(2)
74	U1B	O2	C10	C11B	-162(1)
75	C10	O2	U1	O3	58.3(2)
76	C10	O2	U1	O4	-121.5(2)
77	C10	O2	U1	O1	-25.5(2)
78	C10	O2	U1	O21	146.5(2)
79	C10	O2	U1	O22	158.2(2)
80	C10	O2	U1	O5	-43.1(2)
81	C10	O2	U1	O21B	134.2(5)
82	C10	O2	U1	O22B	147.5(4)

83	U1B	O2	U1	O3	43(1)
84	U1B	O2	U1	O4	-137(1)
85	U1B	O2	U1	O1	-41(1)
86	U1B	O2	U1	O21	131(1)
87	U1B	O2	U1	O22	142(1)
88	U1B	O2	U1	O5	-59(1)
89	U1B	O2	U1	O21B	118(1)
90	U1B	O2	U1	O22B	132(1)
91	C10	O2	U1B	O3	60.3(2)
92	C10	O2	U1B	O4	-124.8(2)
93	C10	O2	U1B	O1	-27.7(2)
94	C10	O2	U1B	O21	147.1(2)
95	C10	O2	U1B	O22	161.9(2)
96	C10	O2	U1B	O5	-51.3(3)
97	C10	O2	U1B	O21B	142.2(5)
98	C10	O2	U1B	O22B	148.2(4)
99	U1	O2	U1B	O3	-134(1)
100	U1	O2	U1B	O4	40(1)
101	U1	O2	U1B	O1	138(1)
102	U1	O2	U1B	O21	-48(1)
103	U1	O2	U1B	O22	-33(1)
104	U1	O2	U1B	O5	114(1)
105	U1	O2	U1B	O21B	-53(1)
106	U1	O2	U1B	O22B	-47(1)
107	O1	C1	C2	C3	-179.1(2)
108	O1	C1	C2	C10	-0.7(3)
109	C8	C1	C2	C3	0.1(2)
110	C8	C1	C2	C10	178.4(1)
111	O1	C1	C8	C7	-1.6(3)
112	O1	C1	C8	C9	-179.2(1)
113	C2	C1	C8	C7	179.2(2)
114	C2	C1	C8	C9	1.6(2)
115	C1	C2	C3	H3A	-120.8
116	C1	C2	C3	H3B	117.7
117	C1	C2	C3	C9	-1.5(2)
118	C10	C2	C3	H3A	61
119	C10	C2	C3	H3B	-60.4
120	C10	C2	C3	C9	-179.7(2)
121	C1	C2	C10	O2	4.6(2)
122	C1	C2	C10	C11	-173.7(2)

123	C1	C2	C10	C11B	-175(1)
124	C3	C2	C10	O2	-177.4(2)
125	C3	C2	C10	C11	4.3(3)
126	C3	C2	C10	C11B	3(1)
127	C2	C3	C9	C4	-176.8(2)
128	C2	C3	C9	C8	2.5(2)
129	H3A	C3	C9	C4	-57.5
130	H3A	C3	C9	C8	121.8
131	H3B	C3	C9	C4	63.9
132	H3B	C3	C9	C8	-116.8
133	H4	C4	C5	H5	1
134	H4	C4	C5	C6	-179
135	C9	C4	C5	H5	-179
136	C9	C4	C5	C6	1.0(3)
137	H4	C4	C9	C3	-0.8
138	H4	C4	C9	C8	179.9
139	C5	C4	C9	C3	179.2(2)
140	C5	C4	C9	C8	-0.1(3)
141	C4	C5	C6	H6	179.2
142	C4	C5	C6	C7	-0.8(3)
143	H5	C5	C6	H6	-0.8
144	H5	C5	C6	C7	179.2
145	C5	C6	C7	H7	179.7
146	C5	C6	C7	C8	-0.3(3)
147	H6	C6	C7	H7	-0.4
148	H6	C6	C7	C8	179.7
149	C6	C7	C8	C1	-176.2(2)
150	C6	C7	C8	C9	1.3(2)
151	H7	C7	C8	C1	3.8
152	H7	C7	C8	C9	-178.7
153	C1	C8	C9	C3	-2.6(2)
154	C1	C8	C9	C4	176.8(2)
155	C7	C8	C9	C3	179.5(1)
156	C7	C8	C9	C4	-1.1(2)
157	O2	C10	C11	C12	-0.2(4)
158	O2	C10	C11	C16	179.3(3)
159	O2	C10	C11	C12B	-175(1)
160	O2	C10	C11	C16B	3(2)
161	C2	C10	C11	C12	178.2(3)
162	C2	C10	C11	C16	-2.3(4)
163	C2	C10	C11	C12B	3(1)
164	C2	C10	C11	C16B	-178(2)

165	C11B	C10	C11	C12	-175(9)
166	C11B	C10	C11	C16	5(8)
167	C11B	C10	C11	C12B	10(9)
168	C11B	C10	C11	C16B	-171(9)
169	O2	C10	C11B	C12	-1(2)
170	O2	C10	C11B	C16	-179(2)
171	O2	C10	C11B	C12B	-171(3)
172	O2	C10	C11B	C16B	1(3)
173	C2	C10	C11B	C12	177.8(9)
174	C2	C10	C11B	C16	-0(4)
175	C2	C10	C11B	C12B	8(4)
176	C2	C10	C11B	C16B	-179(2)
177	C11	C10	C11B	C12	4(7)
178	C11	C10	C11B	C16	-174(11)
179	C11	C10	C11B	C12B	-166(12)
180	C11	C10	C11B	C16B	7(7)
181	C10	C11	C12	H12	-0.6
182	C10	C11	C12	N13	179.3(4)
183	C10	C11	C12	C11B	174(10)
184	C10	C11	C12	C15B	169(2)
185	C10	C11	C12	H16B	2.2
186	C16	C11	C12	H12	179.9
187	C16	C11	C12	N13	-0.1(7)
188	C16	C11	C12	C11B	-5(10)
189	C16	C11	C12	C15B	-10(2)
190	C16	C11	C12	H16B	-177.3
191	C12B	C11	C12	H12	175.5
192	C12B	C11	C12	N13	-5(1)
193	C12B	C11	C12	C11B	-10(10)
194	C12B	C11	C12	C15B	-15(2)
195	C12B	C11	C12	H16B	178.3
196	C16B	C11	C12	H12	-34
197	C16B	C11	C12	N13	146(16)
198	C16B	C11	C12	C11B	141(18)
199	C16B	C11	C12	C15B	136(16)
200	C16B	C11	C12	H16B	-31
201	C10	C11	C16	C15	-177.0(3)
202	C10	C11	C16	H16	3
203	C10	C11	C16	C11B	-23(42)
204	C10	C11	C16	H12B	28.8
205	C10	C11	C16	N13B	179(1)
206	C12	C11	C16	C15	2.5(5)
207	C12	C11	C16	H16	-177.5
208	C12	C11	C16	C11B	156(42)

209	C12	C11	C16	H12B	-151.7
210	C12	C11	C16	N13B	-2(1)
211	C12B	C11	C16	C15	29(5)
212	C12B	C11	C16	H16	-151
213	C12B	C11	C16	C11B	-177(42)
214	C12B	C11	C16	H12B	-125
215	C12B	C11	C16	N13B	25(5)
216	C16B	C11	C16	C15	-1(2)
217	C16B	C11	C16	H16	179
218	C16B	C11	C16	C11B	152(42)
219	C16B	C11	C16	H12B	-156
220	C16B	C11	C16	N13B	-6(2)
221	C10	C11	C12B	C15	-170(1)
222	C10	C11	C12B	H16	-8
223	C10	C11	C12B	C11B	-28(23)
224	C10	C11	C12B	H12B	5
225	C10	C11	C12B	N13B	-177(2)
226	C12	C11	C12B	C15	15(2)
227	C12	C11	C12B	H16	177
228	C12	C11	C12B	C11B	157(23)
229	C12	C11	C12B	H12B	-171
230	C12	C11	C12B	N13B	7(2)
231	C16	C11	C12B	C15	-141(6)
232	C16	C11	C12B	H16	21
233	C16	C11	C12B	C11B	1(23)
234	C16	C11	C12B	H12B	34
235	C16	C11	C12B	N13B	-148(6)
236	C16B	C11	C12B	C15	12(3)
237	C16B	C11	C12B	H16	173
238	C16B	C11	C12B	C11B	154(23)
239	C16B	C11	C12B	H12B	-174
240	C16B	C11	C12B	N13B	4(3)
241	C10	C11	C16B	H12	-11
242	C10	C11	C16B	N13	-177(2)
243	C10	C11	C16B	C11B	170(11)
244	C10	C11	C16B	C15B	175(3)
245	C10	C11	C16B	H16B	-5
246	C12	C11	C16B	H12	137
247	C12	C11	C16B	N13	-29(13)
248	C12	C11	C16B	C11B	-42(20)
249	C12	C11	C16B	C15B	-37(14)
250	C12	C11	C16B	H16B	143
251	C16	C11	C16B	H12	173
252	C16	C11	C16B	N13	7(4)
253	C16	C11	C16B	C11B	-7(10)

254	C16	C11	C16B	C15B	-1(4)
255	C16	C11	C16B	H16B	179
256	C12B	C11	C16B	H12	168
257	C12B	C11	C16B	N13	2(3)
258	C12B	C11	C16B	C11B	-12(10)
259	C12B	C11	C16B	C15B	-6(4)
260	C12B	C11	C16B	H16B	174
261	C11	C12	H12	C16B	19
262	N13	C12	H12	C16B	-161
263	C11B	C12	H12	C16B	18
264	C15B	C12	H12	C16B	-152
265	H16B	C12	H12	C16B	11
266	C11	C12	N13	C14	-2.1(8)
267	C11	C12	N13	C14B	-1(3)
268	C11	C12	N13	H15B	-160
269	C11	C12	N13	C16B	-41(18)
270	H12	C12	N13	C14	177.9
271	H12	C12	N13	C14B	178
272	H12	C12	N13	H15B	20
273	H12	C12	N13	C16B	139
274	C11B	C12	N13	C14	-1(1)
275	C11B	C12	N13	C14B	-1(3)
276	C11B	C12	N13	H15B	-159
277	C11B	C12	N13	C16B	-40(18)
278	C15B	C12	N13	C14	101(11)
279	C15B	C12	N13	C14B	102(11)
280	C15B	C12	N13	H15B	-57
281	C15B	C12	N13	C16B	63(21)
282	H16B	C12	N13	C14	173.8
283	H16B	C12	N13	C14B	174
284	H16B	C12	N13	H15B	15.9
285	H16B	C12	N13	C16B	135
286	C11	C12	C11B	C10	-5(8)
287	C11	C12	C11B	C16	173(12)
288	C11	C12	C11B	C12B	168(12)
289	C11	C12	C11B	C16B	-34(16)
290	H12	C12	C11B	C10	1
291	H12	C12	C11B	C16	179
292	H12	C12	C11B	C12B	174
293	H12	C12	C11B	C16B	-29
294	N13	C12	C11B	C10	179.9(9)
295	N13	C12	C11B	C16	-2(3)
296	N13	C12	C11B	C12B	-7(3)
297	N13	C12	C11B	C16B	151(14)
298	C15B	C12	C11B	C10	170(2)

299	C15B	C12	C11B	C16	-11(4)
300	C15B	C12	C11B	C12B	-16(3)
301	C15B	C12	C11B	C16B	141(14)
302	H16B	C12	C11B	C10	3
303	H16B	C12	C11B	C16	-178
304	H16B	C12	C11B	C12B	177
305	H16B	C12	C11B	C16B	-26
306	C11	C12	C15B	C14	16(4)
307	C11	C12	C15B	C14B	18(5)
308	C11	C12	C15B	H15B	-168
309	C11	C12	C15B	C16B	-46(16)
310	H12	C12	C15B	C14	-174
311	H12	C12	C15B	C14B	-172
312	H12	C12	C15B	H15B	2
313	H12	C12	C15B	C16B	124
314	N13	C12	C15B	C14	-67(10)
315	N13	C12	C15B	C14B	-64(10)
316	N13	C12	C15B	H15B	110
317	N13	C12	C15B	C16B	-128(19)
318	C11B	C12	C15B	C14	15(4)
319	C11B	C12	C15B	C14B	18(5)
320	C11B	C12	C15B	H15B	-168
321	C11B	C12	C15B	C16B	-46(16)
322	H16B	C12	C15B	C14	178
323	H16B	C12	C15B	C14B	-180
324	H16B	C12	C15B	H15B	-6
325	H16B	C12	C15B	C16B	116
326	C11	C12	H16B	C16B	19
327	H12	C12	H16B	C16B	-168
328	N13	C12	H16B	C16B	-157
329	C11B	C12	H16B	C16B	18
330	C15B	C12	H16B	C16B	-146
331	C12	H12	C16B	C11	-153
332	C12	H12	C16B	N13	15
333	C12	H12	C16B	C11B	-153
334	C12	H12	C16B	C15B	22
335	C12	H12	C16B	H16B	-169
336	C12	N13	C14	H14	-178
337	C12	N13	C14	C15	2(1)
338	C12	N13	C14	N13B	9(2)
339	C12	N13	C14	H14B	177.6
340	C12	N13	C14	C15B	-58(10)
341	C14B	N13	C14	H14	9
342	C14B	N13	C14	C15	-171(38)
343	C14B	N13	C14	N13B	-164(38)

344	C14B	N13	C14	H14B	5
345	C14B	N13	C14	C15B	129(39)
346	H15B	N13	C14	H14	-28
347	H15B	N13	C14	C15	152.1
348	H15B	N13	C14	N13B	159
349	H15B	N13	C14	H14B	-32
350	H15B	N13	C14	C15B	92
351	C16B	N13	C14	H14	-175
352	C16B	N13	C14	C15	5(2)
353	C16B	N13	C14	N13B	11(2)
354	C16B	N13	C14	H14B	-180
355	C16B	N13	C14	C15B	-56(10)
356	C12	N13	C14B	H14	-177
357	C12	N13	C14B	C15	1(6)
358	C12	N13	C14B	N13B	6(7)
359	C12	N13	C14B	H14B	178
360	C12	N13	C14B	C15B	-60(10)
361	C14	N13	C14B	H14	-169
362	C14	N13	C14B	C15	8(34)
363	C14	N13	C14B	N13B	14(33)
364	C14	N13	C14B	H14B	-174
365	C14	N13	C14B	C15B	-52(40)
366	H15B	N13	C14B	H14	-24
367	H15B	N13	C14B	C15	153
368	H15B	N13	C14B	N13B	159
369	H15B	N13	C14B	H14B	-30
370	H15B	N13	C14B	C15B	93
371	C16B	N13	C14B	H14	-174
372	C16B	N13	C14B	C15	3(6)
373	C16B	N13	C14B	N13B	9(7)
374	C16B	N13	C14B	H14B	-179
375	C16B	N13	C14B	C15B	-57(10)
376	C12	N13	H15B	C15B	43
377	C14	N13	H15B	C15B	-109
378	C14B	N13	H15B	C15B	-112
379	C16B	N13	H15B	C15B	39
380	C12	N13	C16B	C11	134(20)
381	C12	N13	C16B	H12	-36
382	C12	N13	C16B	C11B	136(20)
383	C12	N13	C16B	C15B	-115(22)
384	C12	N13	C16B	H16B	-38
385	C14	N13	C16B	C11	-9(3)
386	C14	N13	C16B	H12	-178
387	C14	N13	C16B	C11B	-7(3)
388	C14	N13	C16B	C15B	103(11)

389	C14	N13	C16B	H16B	180
390	C14B	N13	C16B	C11	-8(4)
391	C14B	N13	C16B	H12	-178
392	C14B	N13	C16B	C11B	-7(4)
393	C14B	N13	C16B	C15B	103(12)
394	C14B	N13	C16B	H16B	-180
395	H15B	N13	C16B	C11	-164
396	H15B	N13	C16B	H12	26
397	H15B	N13	C16B	C11B	-163
398	H15B	N13	C16B	C15B	-53
399	H15B	N13	C16B	H16B	24
400	N13	C14	H14	C14B	-7
401	C15	C14	H14	C14B	173
402	N13B	C14	H14	C14B	168
403	H14B	C14	H14	C14B	9
404	C15B	C14	H14	C14B	-15
405	N13	C14	C15	H15	-179.7
406	N13	C14	C15	C16	0(1)
407	N13	C14	C15	C12B	8(2)
408	N13	C14	C15	C14B	30(117)
409	H14	C14	C15	H15	0
410	H14	C14	C15	C16	-179.7
411	H14	C14	C15	C12B	-172
412	H14	C14	C15	C14B	-150
413	N13B	C14	C15	H15	32
414	N13B	C14	C15	C16	-148(7)
415	N13B	C14	C15	C12B	-140(8)
416	N13B	C14	C15	C14B	-118(117)
417	H14B	C14	C15	H15	6
418	H14B	C14	C15	C16	-173.9
419	H14B	C14	C15	C12B	-166
420	H14B	C14	C15	C14B	-144
421	C15B	C14	C15	H15	-172
422	C15B	C14	C15	C16	8(2)
423	C15B	C14	C15	C12B	16(2)
424	C15B	C14	C15	C14B	38(117)
425	N13	C14	N13B	H15	-167
426	N13	C14	N13B	C16	-10(3)
427	N13	C14	N13B	C12B	-5(3)
428	N13	C14	N13B	C14B	68(129)
429	H14	C14	N13B	H15	19
430	H14	C14	N13B	C16	176
431	H14	C14	N13B	C12B	-179
432	H14	C14	N13B	C14B	-105

433	C15	C14	N13B	H15	-131
434	C15	C14	N13B	C16	26(6)
435	C15	C14	N13B	C12B	30(6)
436	C15	C14	N13B	C14B	104(129)
437	H14B	C14	N13B	H15	26
438	H14B	C14	N13B	C16	-177
439	H14B	C14	N13B	C12B	-173
440	H14B	C14	N13B	C14B	-99
441	C15B	C14	N13B	H15	-158
442	C15B	C14	N13B	C16	-1(3)
443	C15B	C14	N13B	C12B	4(3)
444	C15B	C14	N13B	C14B	78(129)
445	N13	C14	H14B	C14B	-4
446	H14	C14	H14B	C14B	-169
447	C15	C14	H14B	C14B	171
448	N13B	C14	H14B	C14B	166
449	C15B	C14	H14B	C14B	-11
450	N13	C14	C15B	C12	109(11)
451	N13	C14	C15B	C14B	-45(36)
452	N13	C14	C15B	H15B	-67
453	N13	C14	C15B	C16B	114(11)
454	H14	C14	C15B	C12	173
455	H14	C14	C15B	C14B	19
456	H14	C14	C15B	H15B	-2
457	H14	C14	C15B	C16B	178
458	C15	C14	C15B	C12	-15(3)
459	C15	C14	C15B	C14B	-169(35)
460	C15	C14	C15B	H15B	169
461	C15	C14	C15B	C16B	-10(3)
462	N13B	C14	C15B	C12	-10(4)
463	N13B	C14	C15B	C14B	-165(35)
464	N13B	C14	C15B	H15B	174
465	N13B	C14	C15B	C16B	-5(4)
466	H14B	C14	C15B	C12	167
467	H14B	C14	C15B	C14B	12
468	H14B	C14	C15B	H15B	-9
469	H14B	C14	C15B	C16B	172
470	C14	H14	C14B	N13	171
471	C14	H14	C14B	C15	-6
472	C14	H14	C14B	N13B	-11
473	C14	H14	C14B	H14B	-171
474	C14	H14	C14B	C15B	161
475	C14	C15	H15	N13B	-24
476	C16	C15	H15	N13B	156
477	C12B	C15	H15	N13B	149

478	C14B	C15	H15	N13B	-23
479	C14	C15	C16	C11	-2.6(6)
480	C14	C15	C16	H16	177.4
481	C14	C15	C16	C11B	-4(2)
482	C14	C15	C16	H12B	162.6
483	C14	C15	C16	N13B	55(11)
484	H15	C15	C16	C11	177.5
485	H15	C15	C16	H16	-2.5
486	H15	C15	C16	C11B	176
487	H15	C15	C16	H12B	-17.4
488	H15	C15	C16	N13B	-125
489	C12B	C15	C16	C11	-112(9)
490	C12B	C15	C16	H16	68
491	C12B	C15	C16	C11B	-113(9)
492	C12B	C15	C16	H12B	53
493	C12B	C15	C16	N13B	-55(15)
494	C14B	C15	C16	C11	-3(3)
495	C14B	C15	C16	H16	177
496	C14B	C15	C16	C11B	-4(3)
497	C14B	C15	C16	H12B	162
498	C14B	C15	C16	N13B	54(12)
499	C14	C15	C12B	C11	-16(2)
500	C14	C15	C12B	H16	-170
501	C14	C15	C12B	C11B	-19(3)
502	C14	C15	C12B	H12B	169
503	C14	C15	C12B	N13B	60(10)
504	H15	C15	C12B	C11	171
505	H15	C15	C12B	H16	18
506	H15	C15	C12B	C11B	169
507	H15	C15	C12B	H12B	-4
508	H15	C15	C12B	N13B	-112
509	C16	C15	C12B	C11	58(8)
510	C16	C15	C12B	H16	-96
511	C16	C15	C12B	C11B	56(8)
512	C16	C15	C12B	H12B	-117
513	C16	C15	C12B	N13B	134(13)
514	C14B	C15	C12B	C11	-17(3)
515	C14B	C15	C12B	H16	-171
516	C14B	C15	C12B	C11B	-19(4)
517	C14B	C15	C12B	H12B	168
518	C14B	C15	C12B	N13B	59(10)
519	C14	C15	C14B	N13	-149(121)
520	C14	C15	C14B	H14	29
521	C14	C15	C14B	N13B	61(116)

522	C14	C15	C14B	H14B	34
523	C14	C15	C14B	C15B	-141(120)
524	H15	C15	C14B	N13	-179
525	H15	C15	C14B	H14	-1
526	H15	C15	C14B	N13B	31
527	H15	C15	C14B	H14B	4
528	H15	C15	C14B	C15B	-171
529	C16	C15	C14B	N13	2(6)
530	C16	C15	C14B	H14	179
531	C16	C15	C14B	N13B	-148(7)
532	C16	C15	C14B	H14B	-175
533	C16	C15	C14B	C15B	10(5)
534	C12B	C15	C14B	N13	9(6)
535	C12B	C15	C14B	H14	-173
536	C12B	C15	C14B	N13B	-141(8)
537	C12B	C15	C14B	H14B	-168
538	C12B	C15	C14B	C15B	18(5)
539	C15	H15	N13B	C14	141
540	C15	H15	N13B	C16	-18
541	C15	H15	N13B	C12B	-23
542	C15	H15	N13B	C14B	142
543	C11	C16	H16	C12B	155
544	C15	C16	H16	C12B	-25
545	C11B	C16	H16	C12B	156
546	H12B	C16	H16	C12B	6
547	N13B	C16	H16	C12B	-20
548	C11	C16	C11B	C10	155(45)
549	C11	C16	C11B	C12	-22(40)
550	C11	C16	C11B	C12B	3(44)
551	C11	C16	C11B	C16B	-26(40)
552	C15	C16	C11B	C10	-178(2)
553	C15	C16	C11B	C12	4(3)
554	C15	C16	C11B	C12B	29(5)
555	C15	C16	C11B	C16B	1(4)
556	H16	C16	C11B	C10	1
557	H16	C16	C11B	C12	-177
558	H16	C16	C11B	C12B	-152
559	H16	C16	C11B	C16B	180
560	H12B	C16	C11B	C10	25
561	H12B	C16	C11B	C12	-153
562	H12B	C16	C11B	C12B	-127
563	H12B	C16	C11B	C16B	-156
564	N13B	C16	C11B	C10	178(2)
565	N13B	C16	C11B	C12	-0(3)

566	N13B	C16	C11B	C12B	25(5)
567	N13B	C16	C11B	C16B	-3(4)
568	C11	C16	H12B	C12B	137
569	C15	C16	H12B	C12B	-20
570	H16	C16	H12B	C12B	-174
571	C11B	C16	H12B	C12B	140
572	N13B	C16	H12B	C12B	-16
573	C11	C16	N13B	C14	6(2)
574	C11	C16	N13B	H15	172
575	C11	C16	N13B	C12B	-119(10)
576	C11	C16	N13B	C14B	5(3)
577	C15	C16	N13B	C14	-118(12)
578	C15	C16	N13B	H15	47
579	C15	C16	N13B	C12B	117(16)
580	C15	C16	N13B	C14B	-119(13)
581	H16	C16	N13B	C14	-178
582	H16	C16	N13B	H15	-13
583	H16	C16	N13B	C12B	57
584	H16	C16	N13B	C14B	-179
585	C11B	C16	N13B	C14	5(3)
586	C11B	C16	N13B	H15	171
587	C11B	C16	N13B	C12B	-120(10)
588	C11B	C16	N13B	C14B	4(4)
589	H12B	C16	N13B	C14	169
590	H12B	C16	N13B	H15	-25
591	H12B	C16	N13B	C12B	44
592	H12B	C16	N13B	C14B	168
593	C16	H16	C12B	C11	-18
594	C16	H16	C12B	C15	140
595	C16	H16	C12B	C11B	-17
596	C16	H16	C12B	H12B	-173
597	C16	H16	C12B	N13B	147
598	O3	U1	O21	C21	-119.8(2)
599	O3	U1	O21	U1B	54(1)
600	O3	U1	O21	C30B	-114.1(5)
601	O4	U1	O21	C21	58.7(2)
602	O4	U1	O21	U1B	-128(1)
603	O4	U1	O21	C30B	64.4(5)
604	O1	U1	O21	C21	157.6(2)
605	O1	U1	O21	U1B	-29(1)
606	O1	U1	O21	C30B	163.3(5)
607	O2	U1	O21	C21	145.7(2)
608	O2	U1	O21	U1B	-41(1)

609	O2	U1	O21	C30B	151.4(5)
610	O22	U1	O21	C21	-26.9(2)
611	O22	U1	O21	U1B	147(1)
612	O22	U1	O21	C30B	-21.2(5)
613	O5	U1	O21	C21	-22.7(3)
614	O5	U1	O21	U1B	151(1)
615	O5	U1	O21	C30B	-17.1(5)
616	O21B	U1	O21	C21	-40.3(3)
617	O21B	U1	O21	U1B	133(1)
618	O21B	U1	O21	C30B	-34.7(5)
619	O22B	U1	O21	C21	-28(3)
620	O22B	U1	O21	U1B	146(3)
621	O22B	U1	O21	C30B	-22(3)
622	O3	U1	O22	C30	127.9(2)
623	O3	U1	O22	U1B	-8(2)
624	O3	U1	O22	O21B	-31.8(8)
625	O3	U1	O22	C21B	124(2)
626	O4	U1	O22	C30	-53.2(2)
627	O4	U1	O22	U1B	171(2)
628	O4	U1	O22	O21B	147.2(8)
629	O4	U1	O22	C21B	-57(2)
					-
630	O1	U1	O22	C30	146.2(1)
631	O1	U1	O22	U1B	78(2)
632	O1	U1	O22	O21B	54.2(8)
633	O1	U1	O22	C21B	-150(2)
634	O2	U1	O22	C30	27.5(2)
635	O2	U1	O22	U1B	-108(2)
					-
636	O2	U1	O22	O21B	132.1(8)
637	O2	U1	O22	C21B	24(2)
638	O21	U1	O22	C30	39.1(2)
639	O21	U1	O22	U1B	-96(2)
					-
640	O21	U1	O22	O21B	120.5(8)
641	O21	U1	O22	C21B	36(2)
					-
642	O5	U1	O22	C30	138.5(2)
643	O5	U1	O22	U1B	86(2)
644	O5	U1	O22	O21B	61.8(8)
645	O5	U1	O22	C21B	-142(2)
646	O21B	U1	O22	C30	159.6(9)
647	O21B	U1	O22	U1B	24(2)
648	O21B	U1	O22	C21B	156(2)
649	O22B	U1	O22	C30	39.3(5)

650	O22B	U1	O22	U1B	-96(2)
651	O22B	U1	O22	O21B	-120(1)
652	O22B	U1	O22	C21B	36(2)
653	O3	U1	O5	S1	-34.0(1)
654	O3	U1	O5	U1B	-57(1)
655	O4	U1	O5	S1	146.9(1)
656	O4	U1	O5	U1B	124(1)
657	O1	U1	O5	S1	50.1(1)
658	O1	U1	O5	U1B	28(1)
659	O2	U1	O5	S1	67.5(1)
660	O2	U1	O5	U1B	45(1)
661	O21	U1	O5	S1	-129.7(1)
662	O21	U1	O5	U1B	-152(1)
663	O22	U1	O5	S1	-125.6(1)
664	O22	U1	O5	U1B	-148(1)
665	O21B	U1	O5	S1	-111.1(3)
666	O21B	U1	O5	U1B	-134(1)
667	O22B	U1	O5	S1	-128.6(6)
668	O22B	U1	O5	U1B	-151(1)
669	O3	U1	O21B	O22	147.7(8)
670	O3	U1	O21B	U1B	-15(2)
671	O3	U1	O21B	C21B	141.6(8)
672	O4	U1	O21B	O22	-33.2(8)
673	O4	U1	O21B	U1B	164(2)
674	O4	U1	O21B	C21B	-39.4(8)
675	O1	U1	O21B	O22	-140.3(7)
676	O1	U1	O21B	U1B	57(2)
677	O1	U1	O21B	C21B	-146.5(6)
678	O2	U1	O21B	O22	68(1)
679	O2	U1	O21B	U1B	-95(2)
680	O2	U1	O21B	C21B	62(1)
681	O21	U1	O21B	O22	56.2(8)
682	O21	U1	O21B	U1B	-107(2)
683	O21	U1	O21B	C21B	50.1(7)
684	O22	U1	O21B	U1B	-163(2)
685	O22	U1	O21B	C21B	-6.1(5)
686	O5	U1	O21B	O22	-113.7(8)
687	O5	U1	O21B	U1B	83(2)

688	O5	U1	O21B	C21B	-119.8(8)
689	O22B	U1	O21B	O22	54.3(9)
690	O22B	U1	O21B	U1B	-108(2)
691	O22B	U1	O21B	C21B	48.2(8)
692	O3	U1	O22B	C21	-142(2)
693	O3	U1	O22B	U1B	52(1)
694	O3	U1	O22B	C30B	-124(1)
695	O4	U1	O22B	C21	37(2)
696	O4	U1	O22B	U1B	-130(1)
697	O4	U1	O22B	C30B	55(1)
698	O1	U1	O22B	C21	137(2)
699	O1	U1	O22B	U1B	-29(1)
700	O1	U1	O22B	C30B	155(1)
701	O2	U1	O22B	C21	124(2)
702	O2	U1	O22B	U1B	-42(1)
703	O2	U1	O22B	C30B	142(1)
704	O21	U1	O22B	C21	130(5)
705	O21	U1	O22B	U1B	-36(3)
706	O21	U1	O22B	C30B	148(4)
707	O22	U1	O22B	C21	-49(2)
708	O22	U1	O22B	U1B	145(1)
709	O22	U1	O22B	C30B	-31(1)
710	O5	U1	O22B	C21	-45(3)
711	O5	U1	O22B	U1B	148(1)
712	O5	U1	O22B	C30B	-28(2)
713	O21B	U1	O22B	C21	-62(2)
714	O21B	U1	O22B	U1B	131(1)
715	O21B	U1	O22B	C30B	-45(1)
716	U1	O21	C21	C22	14.2(4)
717	U1	O21	C21	C28	-166.2(2)
718	U1	O21	C21	O22B	35(3)
719	U1	O21	C21	C31B	-162(1)
720	U1B	O21	C21	C22	13.7(4)
721	U1B	O21	C21	C28	-166.7(2)
722	U1B	O21	C21	O22B	35(3)
723	U1B	O21	C21	C31B	-163(1)
724	C30B	O21	C21	C22	-5(2)
725	C30B	O21	C21	C28	175(2)
726	C30B	O21	C21	O22B	16(4)
727	C30B	O21	C21	C31B	179(2)
728	U1	O21	U1B	O3	-122(1)
729	U1	O21	U1B	O4	49(1)

730	U1	O21	U1B	O1	147(1)
731	U1	O21	U1B	O2	138(1)
732	U1	O21	U1B	O22	-32(1)
733	U1	O21	U1B	O5	-25(1)
734	U1	O21	U1B	O21B	-45(1)
735	U1	O21	U1B	O22B	-34(3)
736	C21	O21	U1B	O3	-115.9(3)
737	C21	O21	U1B	O4	55.9(3)
738	C21	O21	U1B	O1	153.1(2)
739	C21	O21	U1B	O2	144.4(3)
740	C21	O21	U1B	O22	-25.4(3)
741	C21	O21	U1B	O5	-18.1(4)
742	C21	O21	U1B	O21B	-38.1(3)
743	C21	O21	U1B	O22B	-27(3)
744	C30B	O21	U1B	O3	-110.3(5)
745	C30B	O21	U1B	O4	61.5(5)
746	C30B	O21	U1B	O1	158.8(5)
747	C30B	O21	U1B	O2	150.1(5)
748	C30B	O21	U1B	O22	-19.7(5)
749	C30B	O21	U1B	O5	-12.4(6)
750	C30B	O21	U1B	O21B	-32.5(5)
751	C30B	O21	U1B	O22B	-22(3)
752	U1	O21	C30B	C22	5(2)
753	U1	O21	C30B	C28	-167.7(2)
754	U1	O21	C30B	O22B	37(5)
755	U1	O21	C30B	C22B	13(1)
756	U1	O21	C30B	C31B	-164.8(7)
757	C21	O21	C30B	C22	169(3)
758	C21	O21	C30B	C28	-4(1)
759	C21	O21	C30B	O22B	-159(5)
760	C21	O21	C30B	C22B	177(2)
761	C21	O21	C30B	C31B	-1(1)
762	U1B	O21	C30B	C22	4(2)
763	U1B	O21	C30B	C28	-168.4(2)
764	U1B	O21	C30B	O22B	37(5)
765	U1B	O21	C30B	C22B	13(1)
766	U1B	O21	C30B	C31B	-165.6(7)
767	U1	O22	C30	C22	-36.6(3)
768	U1	O22	C30	C31	144.8(1)

769	U1	O22	C30	C21B	-177(1)
770	U1	O22	C30	C22B	-40.6(9)
771	U1B	O22	C30	C22	-34.9(3)
772	U1B	O22	C30	C31	146.5(2)
773	U1B	O22	C30	C21B	-176(1)
774	U1B	O22	C30	C22B	-39.0(9)
775	O21B	O22	C30	C22	108(1)
776	O21B	O22	C30	C31	-71(1)
777	O21B	O22	C30	C21B	-33(2)
778	O21B	O22	C30	C22B	104(2)
779	C21B	O22	C30	C22	141(1)
780	C21B	O22	C30	C31	-38(1)
781	C21B	O22	C30	C22B	137(2)
782	U1	O22	U1B	O3	172(2)
783	U1	O22	U1B	O4	-9(2)
784	U1	O22	U1B	O1	-96(2)
785	U1	O22	U1B	O2	67(2)
786	U1	O22	U1B	O21	82(2)
787	U1	O22	U1B	O5	-93(2)
788	U1	O22	U1B	O21B	-156(2)
789	U1	O22	U1B	O22B	83(2)
790	C30	O22	U1B	O3	126.4(2)
791	C30	O22	U1B	O4	-54.0(2)
792	C30	O22	U1B	O1	-141.6(2)
793	C30	O22	U1B	O2	21.7(3)
794	C30	O22	U1B	O21	36.8(2)
795	C30	O22	U1B	O5	-138.4(2)
796	C30	O22	U1B	O21B	158.7(9)
797	C30	O22	U1B	O22B	37.2(5)
798	O21B	O22	U1B	O3	-32.2(8)
799	O21B	O22	U1B	O4	147.3(8)
800	O21B	O22	U1B	O1	59.7(9)
801	O21B	O22	U1B	O2	-137.0(9)
802	O21B	O22	U1B	O21	-121.8(8)
803	O21B	O22	U1B	O5	62.9(8)
804	O21B	O22	U1B	O22B	-121(1)
805	C21B	O22	U1B	O3	120(2)
806	C21B	O22	U1B	O4	-60(2)
807	C21B	O22	U1B	O1	-148(2)
808	C21B	O22	U1B	O2	16(2)
809	C21B	O22	U1B	O21	31(2)

810	C21B	O22	U1B	O5	-145(2)
811	C21B	O22	U1B	O21B	153(2)
812	C21B	O22	U1B	O22B	31(2)
813	U1	O22	O21B	U1B	0.71(7)
814	U1	O22	O21B	C21B	170.9(8)
815	C30	O22	O21B	U1	-154(1)
816	C30	O22	O21B	U1B	-153(1)
817	C30	O22	O21B	C21B	17(1)
818	U1B	O22	O21B	U1	-0.71(7)
819	U1B	O22	O21B	C21B	170.2(8)
820	C21B	O22	O21B	U1	-170.9(8)
821	C21B	O22	O21B	U1B	-170.2(8)
822	U1	O22	C21B	C30	6(3)
823	U1	O22	C21B	O21B	-153(2)
824	U1	O22	C21B	C22B	-13(2)
825	U1	O22	C21B	C28B	137(1)
826	C30	O22	C21B	O21B	-158(1)
827	C30	O22	C21B	C22B	-19.0(7)
828	C30	O22	C21B	C28B	131(2)
829	U1B	O22	C21B	C30	10(3)
830	U1B	O22	C21B	O21B	-149(2)
831	U1B	O22	C21B	C22B	-9(3)
832	U1B	O22	C21B	C28B	141(1)
833	O21B	O22	C21B	C30	158(1)
834	O21B	O22	C21B	C22B	140(1)
835	O21B	O22	C21B	C28B	-70(2)
836	O21	C21	C22	C23	179.5(2)
837	O21	C21	C22	C30	7.1(3)
838	O21	C21	C22	C22B	12(1)
839	O21	C21	C22	C30B	25(8)
840	C28	C21	C22	C23	-0.2(2)
841	C28	C21	C22	C30	-172.6(2)
842	C28	C21	C22	C22B	-168(1)
843	C28	C21	C22	C30B	-154(8)
844	O22B	C21	C22	C23	174(1)
845	O22B	C21	C22	C30	1(1)
846	O22B	C21	C22	C22B	6(2)
847	O22B	C21	C22	C30B	20(8)
848	C31B	C21	C22	C23	-2.9(7)
849	C31B	C21	C22	C30	-175.3(7)
850	C31B	C21	C22	C22B	-170(1)

851	C31B	C21	C22	C30B	-157(8)
852	O21	C21	C28	C27	2.3(4)
853	O21	C21	C28	C29	-179.0(2)
854	O21	C21	C28	C30B	-175(2)
855	O21	C21	C28	C32B	-179(1)
856	O21	C21	C28	C36B	12.4(7)
857	C22	C21	C28	C27	-178.0(2)
858	C22	C21	C28	C29	0.7(3)
859	C22	C21	C28	C30B	5(2)
860	C22	C21	C28	C32B	1(1)
861	C22	C21	C28	C36B	-167.9(7)
862	O22B	C21	C28	C27	10(1)
863	O22B	C21	C28	C29	-171(1)
864	O22B	C21	C28	C30B	-167(2)
865	O22B	C21	C28	C32B	-171(2)
866	O22B	C21	C28	C36B	20(1)
867	C31B	C21	C28	C27	-167(3)
868	C31B	C21	C28	C29	12(3)
869	C31B	C21	C28	C30B	16(3)
870	C31B	C21	C28	C32B	12(3)
871	C31B	C21	C28	C36B	-157(3)
872	O21	C21	O22B	U1	-125(5)
873	O21	C21	O22B	U1B	-123(5)
874	O21	C21	O22B	C30B	-165(3)
875	C22	C21	O22B	U1	37(2)
876	C22	C21	O22B	U1B	38(3)
877	C22	C21	O22B	C30B	-4(1)
878	C28	C21	O22B	U1	-151(1)
879	C28	C21	O22B	U1B	-150(2)
880	C28	C21	O22B	C30B	168(2)
881	C31B	C21	O22B	U1	-150(2)
882	C31B	C21	O22B	U1B	-148(2)
883	C31B	C21	O22B	C30B	170(3)
884	O21	C21	C31B	C27	-5(2)
885	O21	C21	C31B	C29	-174(1)
886	O21	C21	C31B	C30B	-179(2)
887	O21	C21	C31B	C32B	-173(2)
888	O21	C21	C31B	C36B	3(2)
889	C22	C21	C31B	C27	178.1(8)
890	C22	C21	C31B	C29	9(2)
891	C22	C21	C31B	C30B	4(1)
892	C22	C21	C31B	C32B	10(3)

					-
893	C22	C21	C31B	C36B	174.2(8)
894	C28	C21	C31B	C27	9(2)
895	C28	C21	C31B	C29	-161(4)
896	C28	C21	C31B	C30B	-165(3)
897	C28	C21	C31B	C32B	-160(5)
898	C28	C21	C31B	C36B	16(2)
899	O22B	C21	C31B	C27	4(3)
900	O22B	C21	C31B	C29	-165(2)
901	O22B	C21	C31B	C30B	-170(3)
902	O22B	C21	C31B	C32B	-164(3)
903	O22B	C21	C31B	C36B	12(3)
904	C21	C22	C23	H23A	-119.5
905	C21	C22	C23	H23B	118.8
906	C21	C22	C23	C29	-0.4(2)
907	C21	C22	C23	C23B	175.8(4)
908	C21	C22	C23	H23D	-154.7
909	C21	C22	C23	C32B	-0(1)
910	C21	C22	C23	H32B	-8.6
911	C30	C22	C23	H23A	52.2
912	C30	C22	C23	H23B	-69.6
913	C30	C22	C23	C29	171.3(2)
914	C30	C22	C23	C23B	-12.5(4)
915	C30	C22	C23	H23D	17
916	C30	C22	C23	C32B	171(1)
917	C30	C22	C23	H32B	163.1
918	C22B	C22	C23	H23A	50
919	C22B	C22	C23	H23B	-72
920	C22B	C22	C23	C29	169(1)
921	C22B	C22	C23	C23B	-15(1)
922	C22B	C22	C23	H23D	14
923	C22B	C22	C23	C32B	169(1)
924	C22B	C22	C23	H32B	160
925	C30B	C22	C23	H23A	-117
926	C30B	C22	C23	H23B	121.2
927	C30B	C22	C23	C29	2.1(8)
928	C30B	C22	C23	C23B	178.3(9)
929	C30B	C22	C23	H23D	-152.2
930	C30B	C22	C23	C32B	2(1)
931	C30B	C22	C23	H32B	-6.1
932	C21	C22	C30	O22	3.2(3)
					-
933	C21	C22	C30	C31	178.3(2)
934	C21	C22	C30	C21B	44(1)
935	C21	C22	C30	C22B	157(5)

936	C23	C22	C30	O22	-167.5(2)
937	C23	C22	C30	C31	11.0(3)
938	C23	C22	C30	C21B	-127(1)
939	C23	C22	C30	C22B	-14(5)
940	C22B	C22	C30	O22	-154(5)
941	C22B	C22	C30	C31	25(5)
942	C22B	C22	C30	C21B	-113(6)
943	C30B	C22	C30	O22	1.4(8)
944	C30B	C22	C30	C31	179.9(8)
945	C30B	C22	C30	C21B	42(2)
946	C30B	C22	C30	C22B	155(5)
947	C21	C22	C22B	C30	-26(6)
948	C21	C22	C22B	C21B	11(4)
949	C21	C22	C22B	C23B	-179.6(5)
950	C21	C22	C22B	C30B	1.5(8)
951	C23	C22	C22B	C30	168(5)
952	C23	C22	C22B	C21B	-156(3)
953	C23	C22	C22B	C23B	14(1)
954	C23	C22	C22B	C30B	-165(1)
955	C30	C22	C22B	C21B	37(4)
956	C30	C22	C22B	C23B	-154(6)
957	C30	C22	C22B	C30B	28(6)
958	C30B	C22	C22B	C30	-28(6)
959	C30B	C22	C22B	C21B	9(4)
960	C30B	C22	C22B	C23B	179(1)
961	C21	C22	C30B	O21	-148(9)
962	C21	C22	C30B	C28	23(7)
963	C21	C22	C30B	O22B	-156(9)
964	C21	C22	C30B	C22B	-167(7)
965	C21	C22	C30B	C31B	21(7)
966	C23	C22	C30B	O21	-175(2)
967	C23	C22	C30B	C28	-4(1)
968	C23	C22	C30B	O22B	177(2)
969	C23	C22	C30B	C22B	166(1)
970	C23	C22	C30B	C31B	-6(1)
971	C30	C22	C30B	O21	14(2)
972	C30	C22	C30B	C28	-174.3(5)
973	C30	C22	C30B	O22B	6(2)
974	C30	C22	C30B	C22B	-5(1)
975	C30	C22	C30B	C31B	-176.5(7)
976	C22B	C22	C30B	O21	19(3)

977	C22B	C22	C30B	C28	-169(1)
978	C22B	C22	C30B	O22B	11(3)
979	C22B	C22	C30B	C31B	-172(1)
980	C22	C23	C29	C24	178.7(3)
981	C22	C23	C29	C28	0.8(3)
982	C22	C23	C29	C31B	3.7(7)
983	C22	C23	C29	H32B	-171.4
984	C22	C23	C29	N33B	177.6(9)
985	H23A	C23	C29	C24	-62.1
986	H23A	C23	C29	C28	119.9
987	H23A	C23	C29	C31B	122.8
988	H23A	C23	C29	H32B	-52.2
989	H23A	C23	C29	N33B	-63
990	H23B	C23	C29	C24	59.6
991	H23B	C23	C29	C28	-118.3
992	H23B	C23	C29	C31B	-115.5
993	H23B	C23	C29	H32B	69.5
994	H23B	C23	C29	N33B	58
995	C23B	C23	C29	C24	159(2)
996	C23B	C23	C29	C28	-19(2)
997	C23B	C23	C29	C31B	-16(2)
998	C23B	C23	C29	H32B	169
999	C23B	C23	C29	N33B	158(2)
1000	H23D	C23	C29	C24	-58.8
1001	H23D	C23	C29	C28	123.3
1002	H23D	C23	C29	C31B	126.1
1003	H23D	C23	C29	H32B	-48.9
1004	H23D	C23	C29	N33B	-60
1005	C32B	C23	C29	C24	-2(10)
1006	C32B	C23	C29	C28	180(10)
1007	C32B	C23	C29	C31B	-177(10)
1008	C32B	C23	C29	H32B	8
1009	C32B	C23	C29	N33B	-4(10)
1010	H32B	C23	C29	C24	-9.9
1011	H32B	C23	C29	C28	172.2
1012	H32B	C23	C29	C31B	175
1013	H32B	C23	C29	N33B	-11
1014	C22	C23	C23B	C31	21(1)
1015	C22	C23	C23B	C32	-120(2)
1016	C22	C23	C23B	C22B	5.2(4)
1017	C22	C23	C23B	H23C	-110.2
1018	C22	C23	C23B	H23D	129.6
1019	C22	C23	C23B	C29B	93(5)
1020	H23A	C23	C23B	C31	-99
1021	H23A	C23	C23B	C32	119

1022	H23A	C23	C23B	C22B	-114.8
1023	H23A	C23	C23B	H23C	129.7
1024	H23A	C23	C23B	H23D	9.5
1025	H23A	C23	C23B	C29B	-27
1026	H23B	C23	C23B	C31	145
1027	H23B	C23	C23B	C32	4
1028	H23B	C23	C23B	C22B	129.4
1029	H23B	C23	C23B	H23C	13.9
1030	H23B	C23	C23B	H23D	-106.3
1031	H23B	C23	C23B	C29B	-143
1032	C29	C23	C23B	C31	41(3)
1033	C29	C23	C23B	C32	-100(3)
1034	C29	C23	C23B	C22B	26(2)
1035	C29	C23	C23B	H23C	-89
1036	C29	C23	C23B	H23D	150
1037	C29	C23	C23B	C29B	114(4)
1038	H23D	C23	C23B	C31	-109
1039	H23D	C23	C23B	C32	110
1040	H23D	C23	C23B	C22B	-124.3
1041	H23D	C23	C23B	H23C	120.2
1042	H23D	C23	C23B	C29B	-36
1043	C32B	C23	C23B	C31	61(12)
1044	C32B	C23	C23B	C32	-80(12)
1045	C32B	C23	C23B	C22B	46(11)
1046	C32B	C23	C23B	H23C	-69
1047	C32B	C23	C23B	H23D	170
1048	C32B	C23	C23B	C29B	134(11)
1049	H32B	C23	C23B	C31	-153.3
1050	H32B	C23	C23B	C32	66
1051	H32B	C23	C23B	C22B	-168.7
1052	H32B	C23	C23B	H23C	76
1053	H32B	C23	C23B	H23D	-44
1054	H32B	C23	C23B	C29B	-81
1055	C22	C23	H23D	C23B	-45.4
1056	H23A	C23	H23D	C23B	-164.8
1057	H23B	C23	H23D	C23B	65.5
1058	C29	C23	H23D	C23B	-169.9
1059	C32B	C23	H23D	C23B	-178
1060	H32B	C23	H23D	C23B	158.6
1061	C22	C23	C32B	C24	178(3)
1062	C22	C23	C32B	C28	1(2)
1063	C22	C23	C32B	C31B	3(2)
1064	C22	C23	C32B	H32B	-170
1065	C22	C23	C32B	N33B	176(4)
1066	H23A	C23	C32B	C24	-61

1067	H23A	C23	C32B	C28	122
1068	H23A	C23	C32B	C31B	124
1069	H23A	C23	C32B	H32B	-49
1070	H23A	C23	C32B	N33B	-63
1071	H23B	C23	C32B	C24	57
1072	H23B	C23	C32B	C28	-120
1073	H23B	C23	C32B	C31B	-118
1074	H23B	C23	C32B	H32B	69
1075	H23B	C23	C32B	N33B	55
1076	C29	C23	C32B	C24	177(13)
1077	C29	C23	C32B	C28	-0(9)
1078	C29	C23	C32B	C31B	2(9)
1079	C29	C23	C32B	H32B	-171
1080	C29	C23	C32B	N33B	175(14)
1081	C23B	C23	C32B	C24	139(9)
1082	C23B	C23	C32B	C28	-39(12)
1083	C23B	C23	C32B	C31B	-36(12)
1084	C23B	C23	C32B	H32B	150
1085	C23B	C23	C32B	N33B	136(9)
1086	H23D	C23	C32B	C24	-52
1087	H23D	C23	C32B	C28	131
1088	H23D	C23	C32B	C31B	133
1089	H23D	C23	C32B	H32B	-41
1090	H23D	C23	C32B	N33B	-54
1091	H32B	C23	C32B	C24	-12
1092	H32B	C23	C32B	C28	171
1093	H32B	C23	C32B	C31B	174
1094	H32B	C23	C32B	N33B	-14
1095	C22	C23	H32B	C29	13.1
1096	C22	C23	H32B	C32B	15
1097	H23A	C23	H32B	C29	132.4
1098	H23A	C23	H32B	C32B	134
1099	H23B	C23	H32B	C29	-118.7
1100	H23B	C23	H32B	C32B	-117
1101	C29	C23	H32B	C32B	1
1102	C23B	C23	H32B	C29	-175.6
1103	C23B	C23	H32B	C32B	-174
1104	H23D	C23	H32B	C29	154
1105	H23D	C23	H32B	C32B	155
1106	C32B	C23	H32B	C29	-1
1107	C25	C24	H24	N33B	1
1108	C29	C24	H24	N33B	-179
1109	C32B	C24	H24	N33B	-180
1110	C34B	C24	H24	N33B	-4
1111	H24	C24	C25	H25	0.4

1112	H24	C24	C25	C26	-179.6
1113	H24	C24	C25	N33B	-176
1114	H24	C24	C25	C34B	-169
1115	H24	C24	C25	H34B	-37
1116	C29	C24	C25	H25	-179.6
1117	C29	C24	C25	C26	0.5(4)
1118	C29	C24	C25	N33B	4(10)
1119	C29	C24	C25	C34B	11(1)
1120	C29	C24	C25	H34B	143
1121	C32B	C24	C25	H25	-179
1122	C32B	C24	C25	C26	1(2)
1123	C32B	C24	C25	N33B	4(10)
1124	C32B	C24	C25	C34B	11(2)
1125	C32B	C24	C25	H34B	144
1126	C34B	C24	C25	H25	170
1127	C34B	C24	C25	C26	-10(1)
1128	C34B	C24	C25	N33B	-7(10)
1129	C34B	C24	C25	H34B	132
1130	H24	C24	C29	C23	1.9
1131	H24	C24	C29	C28	179.6
1132	H24	C24	C29	C31B	175
1133	H24	C24	C29	H32B	-4.9
1134	H24	C24	C29	N33B	179
1135	C25	C24	C29	C23	-178.2(3)
1136	C25	C24	C29	C28	-0.4(4)
1137	C25	C24	C29	C31B	-5(1)
1138	C25	C24	C29	H32B	175.1
1139	C25	C24	C29	N33B	-1(2)
1140	C32B	C24	C29	C23	156(97)
1141	C32B	C24	C29	C28	-26(97)
1142	C32B	C24	C29	C31B	-31(97)
1143	C32B	C24	C29	H32B	149
1144	C32B	C24	C29	N33B	-26(97)
1145	C34B	C24	C29	C23	-174.9(5)
1146	C34B	C24	C29	C28	2.8(5)
1147	C34B	C24	C29	C31B	-2(1)
1148	C34B	C24	C29	H32B	178.3
1149	C34B	C24	C29	N33B	2(2)
1150	H24	C24	C32B	C23	3
1151	H24	C24	C32B	C28	179
1152	H24	C24	C32B	C31B	174
1153	H24	C24	C32B	H32B	-4
1154	H24	C24	C32B	N33B	180

1155	C25	C24	C32B	C23	-178(2)
1156	C25	C24	C32B	C28	-1(3)
1157	C25	C24	C32B	C31B	-6(5)
1158	C25	C24	C32B	H32B	175
1159	C25	C24	C32B	N33B	-1(2)
1160	C29	C24	C32B	C23	-23(94)
1161	C29	C24	C32B	C28	153(99)
1162	C29	C24	C32B	C31B	148(100)
1163	C29	C24	C32B	H32B	-30
1164	C29	C24	C32B	N33B	154(97)
1165	C34B	C24	C32B	C23	-174(3)
1166	C34B	C24	C32B	C28	2(3)
1167	C34B	C24	C32B	C31B	-3(4)
1168	C34B	C24	C32B	H32B	179
1169	C34B	C24	C32B	N33B	3(2)
1170	H24	C24	C34B	C25	14
1171	H24	C24	C34B	H25	7.3
1172	H24	C24	C34B	C26	171
1173	H24	C24	C34B	N33B	26
1174	H24	C24	C34B	H34B	5
1175	H24	C24	C34B	C35B	-174.6
1176	C25	C24	C34B	H25	-6.5
1177	C25	C24	C34B	C26	157(3)
1178	C25	C24	C34B	N33B	12(16)
1179	C25	C24	C34B	H34B	-9.3
1180	C25	C24	C34B	C35B	172(2)
1181	C29	C24	C34B	C25	-170(1)
1182	C29	C24	C34B	H25	-176.7
1183	C29	C24	C34B	C26	-13(2)
1184	C29	C24	C34B	N33B	-158(17)
1185	C29	C24	C34B	H34B	-180
1186	C29	C24	C34B	C35B	1(1)
1187	C32B	C24	C34B	C25	-170(2)
1188	C32B	C24	C34B	H25	-176
1189	C32B	C24	C34B	C26	-13(2)
1190	C32B	C24	C34B	N33B	-158(17)
1191	C32B	C24	C34B	H34B	-179
1192	C32B	C24	C34B	C35B	2(2)
1193	C24	H24	N33B	C25	-178
1194	C24	H24	N33B	C29	1
1195	C24	H24	N33B	C32B	0
1196	C24	H24	N33B	C34B	172
1197	C24	C25	H25	C34B	-170
1198	C26	C25	H25	C34B	10
1199	N33B	C25	H25	C34B	-169

1200	H34B	C25	H25	C34B	3
1201	C24	C25	C26	H26	-179.9
1202	C24	C25	C26	C27	0.1(3)
1203	C24	C25	C26	C34B	85(8)
1204	C24	C25	C26	C35B	22(2)
1205	C24	C25	C26	H35B	139.9
1206	H25	C25	C26	H26	0.2
1207	H25	C25	C26	C27	-179.8
1208	H25	C25	C26	C34B	-95
1209	H25	C25	C26	C35B	-158
1210	H25	C25	C26	H35B	-40.1
1211	N33B	C25	C26	H26	179.8
1212	N33B	C25	C26	C27	-0(1)
1213	N33B	C25	C26	C34B	84(8)
1214	N33B	C25	C26	C35B	21(2)
1215	N33B	C25	C26	H35B	140
1216	C34B	C25	C26	H26	95
1217	C34B	C25	C26	C27	-85(8)
1218	C34B	C25	C26	C35B	-63(8)
1219	C34B	C25	C26	H35B	55
1220	H34B	C25	C26	H26	6.9
1221	H34B	C25	C26	C27	-173.2
1222	H34B	C25	C26	C34B	-89
1223	H34B	C25	C26	C35B	-151
1224	H34B	C25	C26	H35B	-33.4
1225	C24	C25	N33B	H24	3
1226	C24	C25	N33B	C29	-176(12)
1227	C24	C25	N33B	C32B	-175(12)
1228	C24	C25	N33B	C34B	173(10)
1229	H25	C25	N33B	H24	0
1230	H25	C25	N33B	C29	-179
1231	H25	C25	N33B	C32B	-179
1232	H25	C25	N33B	C34B	170
1233	C26	C25	N33B	H24	180
1234	C26	C25	N33B	C29	1(2)
1235	C26	C25	N33B	C32B	2(3)
1236	C26	C25	N33B	C34B	-10(1)
1237	C34B	C25	N33B	H24	-170
1238	C34B	C25	N33B	C29	11(2)
1239	C34B	C25	N33B	C32B	12(3)
1240	H34B	C25	N33B	H24	-62
1241	H34B	C25	N33B	C29	120
1242	H34B	C25	N33B	C32B	120
1243	H34B	C25	N33B	C34B	108
1244	C24	C25	C34B	H25	169

1245	C24	C25	C34B	C26	-101(7)
1246	C24	C25	C34B	N33B	-0.7(9)
1247	C24	C25	C34B	H34B	171.8
1248	C24	C25	C34B	C35B	-25(7)
1249	H25	C25	C34B	C24	-169
1250	H25	C25	C34B	C26	90
1251	H25	C25	C34B	N33B	-170
1252	H25	C25	C34B	H34B	2
1253	H25	C25	C34B	C35B	165
1254	C26	C25	C34B	C24	101(7)
1255	C26	C25	C34B	H25	-90
1256	C26	C25	C34B	N33B	100(7)
1257	C26	C25	C34B	H34B	-88
1258	C26	C25	C34B	C35B	75(8)
1259	N33B	C25	C34B	C24	0.7(9)
1260	N33B	C25	C34B	H25	170
1261	N33B	C25	C34B	C26	-100(7)
1262	N33B	C25	C34B	H34B	172
1263	N33B	C25	C34B	C35B	-25(6)
1264	H34B	C25	C34B	C24	-171.8
1265	H34B	C25	C34B	H25	-2
1266	H34B	C25	C34B	C26	88
1267	H34B	C25	C34B	N33B	-172
1268	H34B	C25	C34B	C35B	163
1269	C24	C25	H34B	C34B	-136
1270	H25	C25	H34B	C34B	-177
1271	C26	C25	H34B	C34B	10
1272	N33B	C25	H34B	C34B	-111
1273	C25	H25	C34B	C24	7
1274	C25	H25	C34B	C26	-155
1275	C25	H25	C34B	N33B	7
1276	C25	H25	C34B	H34B	-176
1277	C25	H25	C34B	C35B	-168
1278	C25	C26	H26	C35B	-166
1279	C27	C26	H26	C35B	14
1280	C34B	C26	H26	C35B	-158
1281	H35B	C26	H26	C35B	2
1282	C25	C26	C27	H27	179.3
1283	C25	C26	C27	C28	-0.8(3)
1284	C25	C26	C27	C31B	1.8(6)
1285	C25	C26	C27	C35B	144(2)
1286	C25	C26	C27	C36B	39(2)
1287	C25	C26	C27	H36B	146.6
1288	H26	C26	C27	H27	-0.8
1289	H26	C26	C27	C28	179.2

1290	H26	C26	C27	C31B	-178.2
1291	H26	C26	C27	C35B	-36
1292	H26	C26	C27	C36B	-141
1293	H26	C26	C27	H36B	-33.4
1294	C34B	C26	C27	H27	172
1295	C34B	C26	C27	C28	-8(1)
1296	C34B	C26	C27	C31B	-6(1)
1297	C34B	C26	C27	C35B	137(3)
1298	C34B	C26	C27	C36B	32(3)
1299	C34B	C26	C27	H36B	139
1300	C35B	C26	C27	H27	35
1301	C35B	C26	C27	C28	-145(2)
1302	C35B	C26	C27	C31B	-142(2)
1303	C35B	C26	C27	C36B	-105(3)
1304	C35B	C26	C27	H36B	2
1305	H35B	C26	C27	H27	9.1
1306	H35B	C26	C27	C28	-170.9
1307	H35B	C26	C27	C31B	-168.4
1308	H35B	C26	C27	C35B	-26
1309	H35B	C26	C27	C36B	-131
1310	H35B	C26	C27	H36B	-23.6
1311	C25	C26	C34B	C24	-84(8)
1312	C25	C26	C34B	H25	71
1313	C25	C26	C34B	N33B	-82(8)
1314	C25	C26	C34B	H34B	84
1315	C25	C26	C34B	C35B	-123(7)
1316	H26	C26	C34B	C24	-172
1317	H26	C26	C34B	C25	-88
1318	H26	C26	C34B	H25	-18
1319	H26	C26	C34B	N33B	-170
1320	H26	C26	C34B	H34B	-4
1321	H26	C26	C34B	C35B	148
1322	C27	C26	C34B	C24	16(2)
1323	C27	C26	C34B	C25	99(7)
1324	C27	C26	C34B	H25	170
1325	C27	C26	C34B	N33B	18(2)
1326	C27	C26	C34B	H34B	-176.4
1327	C27	C26	C34B	C35B	-24(2)
1328	C35B	C26	C34B	C24	40(3)
1329	C35B	C26	C34B	C25	123(7)
1330	C35B	C26	C34B	H25	-166
1331	C35B	C26	C34B	N33B	42(3)
1332	C35B	C26	C34B	H34B	-152
1333	H35B	C26	C34B	C24	135
1334	H35B	C26	C34B	C25	-141

1335	H35B	C26	C34B	H25	-70
1336	H35B	C26	C34B	N33B	137
1337	H35B	C26	C34B	H34B	-57
1338	H35B	C26	C34B	C35B	96
1339	C25	C26	C35B	H26	161
1340	C25	C26	C35B	C27	-50(3)
1341	C25	C26	C35B	C34B	8(1)
1342	C25	C26	C35B	H35B	163.1
1343	C25	C26	C35B	C36B	-33(3)
1344	H26	C26	C35B	C27	149
1345	H26	C26	C35B	C34B	-153
1346	H26	C26	C35B	H35B	2
1347	H26	C26	C35B	C36B	166
1348	C27	C26	C35B	H26	-149
1349	C27	C26	C35B	C34B	58(3)
1350	C27	C26	C35B	H35B	-147
1351	C27	C26	C35B	C36B	17(1)
1352	C34B	C26	C35B	H26	153
1353	C34B	C26	C35B	C27	-58(3)
1354	C34B	C26	C35B	H35B	155
1355	C34B	C26	C35B	C36B	-41(3)
1356	H35B	C26	C35B	H26	-2
1357	H35B	C26	C35B	C27	147
1358	H35B	C26	C35B	C34B	-155
1359	H35B	C26	C35B	C36B	164
1360	C25	C26	H35B	C35B	-130
1361	H26	C26	H35B	C35B	-177
1362	C27	C26	H35B	C35B	14
1363	C34B	C26	H35B	C35B	-113
1364	C26	H26	C35B	C27	-113
1365	C26	H26	C35B	C34B	11.6
1366	C26	H26	C35B	H35B	-177
1367	C26	H26	C35B	C36B	-134
1368	C26	C27	H27	C36B	-162
1369	C28	C27	H27	C36B	18
1370	C31B	C27	H27	C36B	15
1371	C35B	C27	H27	C36B	-150
1372	H36B	C27	H27	C36B	0
1373	C26	C27	C28	C21	179.4(2)
1374	C26	C27	C28	C29	0.8(3)
1375	C26	C27	C28	C30B	-179.3(7)
1376	C26	C27	C28	C32B	0(1)
1377	C26	C27	C28	C36B	153(2)
1378	H27	C27	C28	C21	-0.6

1379	H27	C27	C28	C29	-179.2
1380	H27	C27	C28	C30B	0.6
1381	H27	C27	C28	C32B	-180
1382	H27	C27	C28	C36B	-27
1383	C31B	C27	C28	C21	111(11)
1384	C31B	C27	C28	C29	-68(11)
1385	C31B	C27	C28	C30B	112(11)
1386	C31B	C27	C28	C32B	-68(11)
1387	C31B	C27	C28	C36B	85(11)
1388	C35B	C27	C28	C21	164(1)
1389	C35B	C27	C28	C29	-14(1)
1390	C35B	C27	C28	C30B	166(1)
1391	C35B	C27	C28	C32B	-15(2)
1392	C35B	C27	C28	C36B	138(2)
1393	C36B	C27	C28	C21	26(2)
1394	C36B	C27	C28	C29	-152(2)
1395	C36B	C27	C28	C30B	28(2)
1396	C36B	C27	C28	C32B	-153(2)
1397	H36B	C27	C28	C21	11.2
1398	H36B	C27	C28	C29	-167.4
1399	H36B	C27	C28	C30B	12.4
1400	H36B	C27	C28	C32B	-168
1401	H36B	C27	C28	C36B	-15
1402	C26	C27	C31B	C21	-177.2(7)
1403	C26	C27	C31B	C29	-5(1)
1404	C26	C27	C31B	C30B	-175(1)
1405	C26	C27	C31B	C32B	-4(2)
1406	C26	C27	C31B	C36B	155(2)
1407	H27	C27	C31B	C21	5
1408	H27	C27	C31B	C29	178
1409	H27	C27	C31B	C30B	8
1410	H27	C27	C31B	C32B	178
1411	H27	C27	C31B	C36B	-23
1412	C28	C27	C31B	C21	-65(11)
1413	C28	C27	C31B	C29	108(11)
1414	C28	C27	C31B	C30B	-62(11)
1415	C28	C27	C31B	C32B	108(11)
1416	C28	C27	C31B	C36B	-93(11)
1417	C35B	C27	C31B	C21	167(1)
1418	C35B	C27	C31B	C29	-20(2)
1419	C35B	C27	C31B	C30B	170(1)
1420	C35B	C27	C31B	C32B	-20(2)
1421	C35B	C27	C31B	C36B	139(2)
1422	C36B	C27	C31B	C21	28(2)

1423	C36B	C27	C31B	C29	-159(2)
1424	C36B	C27	C31B	C30B	31(2)
1425	C36B	C27	C31B	C32B	-159(2)
1426	H36B	C27	C31B	C21	15.4
1427	H36B	C27	C31B	C29	-172
1428	H36B	C27	C31B	C30B	18
1429	H36B	C27	C31B	C32B	-172
1430	H36B	C27	C31B	C36B	-13
1431	C26	C27	C35B	H26	91
1432	C26	C27	C35B	C34B	-24(2)
1433	C26	C27	C35B	H35B	142
1434	C26	C27	C35B	C36B	-111(3)
1435	H27	C27	C35B	C26	-149
1436	H27	C27	C35B	H26	-58
1437	H27	C27	C35B	C34B	-174
1438	H27	C27	C35B	H35B	-7
1439	H27	C27	C35B	C36B	100
1440	C28	C27	C35B	C26	44(3)
1441	C28	C27	C35B	H26	135
1442	C28	C27	C35B	C34B	20(2)
1443	C28	C27	C35B	H35B	-173.7
1444	C28	C27	C35B	C36B	-67(3)
1445	C31B	C27	C35B	C26	47(3)
1446	C31B	C27	C35B	H26	138
1447	C31B	C27	C35B	C34B	22(2)
1448	C31B	C27	C35B	H35B	-171.1
1449	C31B	C27	C35B	C36B	-64(3)
1450	C36B	C27	C35B	C26	111(3)
1451	C36B	C27	C35B	H26	-158
1452	C36B	C27	C35B	C34B	87(3)
1453	C36B	C27	C35B	H35B	-107
1454	H36B	C27	C35B	C26	-179
1455	H36B	C27	C35B	H26	-87
1456	H36B	C27	C35B	C34B	157.2
1457	H36B	C27	C35B	H35B	-36
1458	H36B	C27	C35B	C36B	71
1459	C26	C27	C36B	H27	146
1460	C26	C27	C36B	C28	-57(3)
1461	C26	C27	C36B	C31B	-53(3)
1462	C26	C27	C36B	C35B	41(2)
1463	C26	C27	C36B	H36B	147
1464	H27	C27	C36B	C28	157
1465	H27	C27	C36B	C31B	161
1466	H27	C27	C36B	C35B	-106
1467	H27	C27	C36B	H36B	0

1468	C28	C27	C36B	H27	-157
1469	C28	C27	C36B	C31B	3.7(7)
1470	C28	C27	C36B	C35B	97(3)
1471	C28	C27	C36B	H36B	-157
1472	C31B	C27	C36B	H27	-161
1473	C31B	C27	C36B	C28	-3.7(7)
1474	C31B	C27	C36B	C35B	94(3)
1475	C31B	C27	C36B	H36B	-160
1476	C35B	C27	C36B	H27	106
1477	C35B	C27	C36B	C28	-97(3)
1478	C35B	C27	C36B	C31B	-94(3)
1479	C35B	C27	C36B	H36B	106
1480	H36B	C27	C36B	H27	0
1481	H36B	C27	C36B	C28	157
1482	H36B	C27	C36B	C31B	160
1483	H36B	C27	C36B	C35B	-106
1484	C26	C27	H36B	C36B	-135
1485	H27	C27	H36B	C36B	-179
1486	C28	C27	H36B	C36B	16
1487	C31B	C27	H36B	C36B	13
1488	C35B	C27	H36B	C36B	-134
1489	C27	H27	C36B	C28	-94
1490	C27	H27	C36B	C31B	-113
1491	C27	H27	C36B	C35B	17.9
1492	C27	H27	C36B	H36B	-180
1493	C21	C28	C29	C23	-1.0(3)
1494	C21	C28	C29	C24	-179.1(2)
1495	C21	C28	C29	C31B	-12(3)
1496	C21	C28	C29	H32B	11.1
1497	C21	C28	C29	N33B	-179.0(5)
1498	C27	C28	C29	C23	177.9(2)
1499	C27	C28	C29	C24	-0.3(4)
1500	C27	C28	C29	C31B	167(3)
1501	C27	C28	C29	H32B	-170.1
1502	C27	C28	C29	N33B	-0.2(6)
1503	C30B	C28	C29	C23	-2.0(4)
1504	C30B	C28	C29	C24	179.8(5)
1505	C30B	C28	C29	C31B	-13(3)
1506	C30B	C28	C29	H32B	10
1507	C30B	C28	C29	N33B	179.9(7)
1508	C32B	C28	C29	C23	-180(8)
1509	C32B	C28	C29	C24	2(8)
1510	C32B	C28	C29	C31B	169(9)

1511	C32B	C28	C29	H32B	-168
1512	C32B	C28	C29	N33B	2(8)
1513	C36B	C28	C29	C23	162(1)
1514	C36B	C28	C29	C24	-16(1)
1515	C36B	C28	C29	C31B	151(3)
1516	C36B	C28	C29	H32B	174
1517	C36B	C28	C29	N33B	-16(1)
1518	C21	C28	C30B	O21	4(1)
1519	C21	C28	C30B	C22	-172(2)
1520	C21	C28	C30B	O22B	8(1)
1521	C21	C28	C30B	C22B	-178(3)
1522	C21	C28	C30B	C31B	-160(4)
1523	C27	C28	C30B	O21	-0(1)
1524	C27	C28	C30B	C22	-175.9(5)
1525	C27	C28	C30B	O22B	4(1)
1526	C27	C28	C30B	C22B	178.5(9)
1527	C27	C28	C30B	C31B	-164(3)
1528	C29	C28	C30B	O21	179.5(4)
1529	C29	C28	C30B	C22	4(1)
1530	C29	C28	C30B	O22B	-176.5(9)
1531	C29	C28	C30B	C22B	-2(1)
1532	C29	C28	C30B	C31B	16(3)
1533	C32B	C28	C30B	O21	180(1)
1534	C32B	C28	C30B	C22	4(2)
1535	C32B	C28	C30B	O22B	-176(1)
1536	C32B	C28	C30B	C22B	-1(2)
1537	C32B	C28	C30B	C31B	16(3)
1538	C36B	C28	C30B	O21	12(1)
1539	C36B	C28	C30B	C22	-164(1)
1540	C36B	C28	C30B	O22B	16(1)
1541	C36B	C28	C30B	C22B	-170(1)
1542	C36B	C28	C30B	C31B	-152(3)
1543	C21	C28	C32B	C23	-1(2)
1544	C21	C28	C32B	C24	-178(2)
1545	C21	C28	C32B	C31B	-11(3)
1546	C21	C28	C32B	H32B	6
1547	C21	C28	C32B	N33B	-179(1)
1548	C27	C28	C32B	C23	178.0(7)
1549	C27	C28	C32B	C24	1(3)
1550	C27	C28	C32B	C31B	168(3)
1551	C27	C28	C32B	H32B	-175
1552	C27	C28	C32B	N33B	0(2)
1553	C29	C28	C32B	C23	0(7)

1554	C29	C28	C32B	C24	-177(11)
1555	C29	C28	C32B	C31B	-10(8)
1556	C29	C28	C32B	H32B	7
1557	C29	C28	C32B	N33B	-177(10)
1558	C30B	C28	C32B	C23	-2(1)
1559	C30B	C28	C32B	C24	-180(2)
1560	C30B	C28	C32B	C31B	-12(3)
1561	C30B	C28	C32B	H32B	5
1562	C30B	C28	C32B	N33B	-180(2)
1563	C36B	C28	C32B	C23	164(1)
1564	C36B	C28	C32B	C24	-13(4)
1565	C36B	C28	C32B	C31B	154(3)
1566	C36B	C28	C32B	H32B	171
1567	C36B	C28	C32B	N33B	-13(3)
1568	C21	C28	C36B	C27	-159(1)
1569	C21	C28	C36B	H27	-75
1570	C21	C28	C36B	C31B	49(5)
1571	C21	C28	C36B	C35B	-178.1(8)
1572	C21	C28	C36B	H36B	-4
1573	C27	C28	C36B	H27	84
1574	C27	C28	C36B	C31B	-153(5)
1575	C27	C28	C36B	C35B	-19(1)
1576	C27	C28	C36B	H36B	155
1577	C29	C28	C36B	C27	38(2)
1578	C29	C28	C36B	H27	123
1579	C29	C28	C36B	C31B	-114(5)
1580	C29	C28	C36B	C35B	19(2)
1581	C29	C28	C36B	H36B	-167
1582	C30B	C28	C36B	C27	-161(1)
1583	C30B	C28	C36B	H27	-77
1584	C30B	C28	C36B	C31B	46(5)
1585	C30B	C28	C36B	C35B	179.7(8)
1586	C30B	C28	C36B	H36B	-6
1587	C32B	C28	C36B	C27	35(3)
1588	C32B	C28	C36B	H27	119
1589	C32B	C28	C36B	C31B	-118(6)
1590	C32B	C28	C36B	C35B	16(2)
1591	C32B	C28	C36B	H36B	-170
1592	C23	C29	C31B	C21	-9(2)
1593	C23	C29	C31B	C27	-178.8(7)
1594	C23	C29	C31B	C30B	-7(1)
1595	C23	C29	C31B	C32B	178(10)
1596	C23	C29	C31B	C36B	174(1)

1597	C24	C29	C31B	C21	177(1)
1598	C24	C29	C31B	C27	7(1)
1599	C24	C29	C31B	C30B	178.2(7)
1600	C24	C29	C31B	C32B	3(10)
1601	C24	C29	C31B	C36B	-0(2)
1602	C28	C29	C31B	C21	161(4)
1603	C28	C29	C31B	C27	-9(2)
1604	C28	C29	C31B	C30B	162(4)
1605	C28	C29	C31B	C32B	-13(10)
1606	C28	C29	C31B	C36B	-16(2)
1607	H32B	C29	C31B	C21	-4
1608	H32B	C29	C31B	C27	-173.6
1609	H32B	C29	C31B	C30B	-2
1610	H32B	C29	C31B	C32B	-177
1611	H32B	C29	C31B	C36B	179.6
1612	N33B	C29	C31B	C21	175(1)
1613	N33B	C29	C31B	C27	5(1)
1614	N33B	C29	C31B	C30B	177(1)
1615	N33B	C29	C31B	C32B	2(10)
1616	N33B	C29	C31B	C36B	-2(2)
1617	C23	C29	H32B	C32B	-175
1618	C24	C29	H32B	C23	172.3
1619	C24	C29	H32B	C32B	-2
1620	C28	C29	H32B	C23	-16.5
1621	C28	C29	H32B	C32B	169
1622	C31B	C29	H32B	C23	-8
1623	C31B	C29	H32B	C32B	178
1624	N33B	C29	H32B	C23	173.5
1625	N33B	C29	H32B	C32B	-1
1626	C23	C29	N33B	H24	3
1627	C23	C29	N33B	C25	-178(1)
1628	C23	C29	N33B	C32B	13(36)
1629	C23	C29	N33B	C34B	-173.0(7)
1630	C24	C29	N33B	H24	0
1631	C24	C29	N33B	C25	179(4)
1632	C24	C29	N33B	C32B	10(36)
1633	C24	C29	N33B	C34B	-176(3)
1634	C28	C29	N33B	H24	179.8
1635	C28	C29	N33B	C25	-1(2)
1636	C28	C29	N33B	C32B	-170(36)
1637	C28	C29	N33B	C34B	4(1)
1638	C31B	C29	N33B	H24	176
1639	C31B	C29	N33B	C25	-5(2)
1640	C31B	C29	N33B	C32B	-174(36)

1641	C31B	C29	N33B	C34B	0(2)
1642	H32B	C29	N33B	H24	-4.7
1643	H32B	C29	N33B	C25	174
1644	H32B	C29	N33B	C32B	5
1645	H32B	C29	N33B	C34B	179
1646	O22	C30	C31	C32	-144.3(2)
1647	O22	C30	C31	C36	30.3(2)
1648	O22	C30	C31	C23B	178.6(4)
1649	O22	C30	C31	C28B	23.6(8)
1650	O22	C30	C31	C29B	174.6(9)
1651	C22	C30	C31	C32	37.1(3)
1652	C22	C30	C31	C36	-148.3(2)
1653	C22	C30	C31	C23B	-0.0(4)
1654	C22	C30	C31	C28B	-155.0(8)
1655	C22	C30	C31	C29B	-4.0(9)
1656	C21B	C30	C31	C32	-165.3(8)
1657	C21B	C30	C31	C36	9.3(8)
1658	C21B	C30	C31	C23B	157.5(9)
1659	C21B	C30	C31	C28B	3(1)
1660	C21B	C30	C31	C29B	154(1)
1661	C22B	C30	C31	C32	40.4(8)
1662	C22B	C30	C31	C36	-145.0(7)
1663	C22B	C30	C31	C23B	3.2(8)
1664	C22B	C30	C31	C28B	-152(1)
1665	C22B	C30	C31	C29B	-1(1)
1666	O22	C30	C21B	O21B	13.9(8)
1667	O22	C30	C21B	C22B	81(2)
1668	O22	C30	C21B	C28B	-147(2)
1669	C22	C30	C21B	O22	-69(2)
1670	C22	C30	C21B	O21B	-55(2)
1671	C22	C30	C21B	C22B	12(1)
1672	C22	C30	C21B	C28B	144.2(9)
1673	C31	C30	C21B	O22	146(1)
1674	C31	C30	C21B	O21B	160(2)
1675	C31	C30	C21B	C22B	-133(2)
1676	C31	C30	C21B	C28B	-1.2(5)
1677	C22B	C30	C21B	O22	-81(2)
1678	C22B	C30	C21B	O21B	-67(3)
1679	C22B	C30	C21B	C28B	132(2)
1680	O22	C30	C22B	C22	29(6)

1681	O22	C30	C22B	C21B	-48(2)
1682	O22	C30	C22B	C23B	-178.0(4)
1683	O22	C30	C22B	C30B	9(2)
1684	C22	C30	C22B	C21B	-77(6)
1685	C22	C30	C22B	C23B	153(6)
1686	C22	C30	C22B	C30B	-20(4)
1687	C31	C30	C22B	C22	-157(5)
1688	C31	C30	C22B	C21B	126(2)
1689	C31	C30	C22B	C23B	-3.5(9)
1690	C31	C30	C22B	C30B	-177(1)
1691	C21B	C30	C22B	C22	77(6)
1692	C21B	C30	C22B	C23B	-130(2)
1693	C21B	C30	C22B	C30B	57(2)
1694	C30	C31	C32	H32	-2.4
1695	C30	C31	C32	N33	177.5(2)
1696	C30	C31	C32	C23B	-39.3(4)
1697	C30	C31	C32	C24B	-159.3(5)
1698	C30	C31	C32	C29B	-115(1)
1699	C36	C31	C32	H32	-177.1
1700	C36	C31	C32	N33	2.8(3)
1701	C36	C31	C32	C23B	146.0(4)
1702	C36	C31	C32	C24B	26.0(5)
1703	C36	C31	C32	C29B	70(1)
1704	C23B	C31	C32	H32	36.9
1705	C23B	C31	C32	N33	-143.2(4)
1706	C23B	C31	C32	C24B	-120.0(6)
1707	C23B	C31	C32	C29B	-76(1)
1708	C28B	C31	C32	H32	-162
1709	C28B	C31	C32	N33	18(1)
1710	C28B	C31	C32	C23B	161(1)
1711	C28B	C31	C32	C24B	41(1)
1712	C28B	C31	C32	C29B	85(2)
1713	C29B	C31	C32	H32	113
1714	C29B	C31	C32	N33	-67(1)
1715	C29B	C31	C32	C23B	76(1)
1716	C29B	C31	C32	C24B	-44(1)
1717	C30	C31	C36	C35	-178.8(2)
1718	C30	C31	C36	H36	1.2
1719	C30	C31	C36	C27B	-75(1)
1720	C30	C31	C36	C28B	-15(2)

1721	C30	C31	C36	C29B	152.7(7)
1722	C32	C31	C36	C35	-4.0(3)
1723	C32	C31	C36	H36	176
1724	C32	C31	C36	C27B	100(1)
1725	C32	C31	C36	C28B	160(2)
1726	C32	C31	C36	C29B	-32.6(7)
1727	C23B	C31	C36	C35	79.8(7)
1728	C23B	C31	C36	H36	-100.2
1729	C23B	C31	C36	C27B	-176(1)
1730	C23B	C31	C36	C28B	-116(2)
1731	C23B	C31	C36	C29B	51(1)
1732	C28B	C31	C36	C35	-164(2)
1733	C28B	C31	C36	H36	16
1734	C28B	C31	C36	C27B	-60(2)
1735	C28B	C31	C36	C29B	167(2)
1736	C29B	C31	C36	C35	28.5(7)
1737	C29B	C31	C36	H36	-151.5
1738	C29B	C31	C36	C27B	132(1)
1739	C29B	C31	C36	C28B	-167(2)
1740	C30	C31	C23B	C23	-17(1)
1741	C30	C31	C23B	C32	145.0(3)
1742	C30	C31	C23B	C22B	-1.6(4)
1743	C30	C31	C23B	H23C	104.3
1744	C30	C31	C23B	H23D	-107
1745	C30	C31	C23B	C29B	-176.5(8)
1746	C32	C31	C23B	C23	-162(1)
1747	C32	C31	C23B	C22B	-146.6(6)
1748	C32	C31	C23B	H23C	-40.7
1749	C32	C31	C23B	H23D	108
1750	C32	C31	C23B	C29B	38.5(8)
1751	C36	C31	C23B	C23	98(1)
1752	C36	C31	C23B	C32	-99.6(6)
1753	C36	C31	C23B	C22B	113.8(7)
1754	C36	C31	C23B	H23C	-140.4
1755	C36	C31	C23B	H23D	8
1756	C36	C31	C23B	C29B	-61(1)
1757	C28B	C31	C23B	C23	42(2)
1758	C28B	C31	C23B	C32	-156(2)
1759	C28B	C31	C23B	C22B	57(2)
1760	C28B	C31	C23B	H23C	163
1761	C28B	C31	C23B	H23D	-48
1762	C28B	C31	C23B	C29B	-118(2)
1763	C29B	C31	C23B	C23	159(2)

1764	C29B	C31	C23B	C32	-38.5(8)
1765	C29B	C31	C23B	C22B	175(1)
1766	C29B	C31	C23B	H23C	-79
1767	C29B	C31	C23B	H23D	69
1768	C30	C31	C28B	C36	167(1)
1769	C30	C31	C28B	H36	114
1770	C30	C31	C28B	C21B	-1.2(5)
1771	C30	C31	C28B	C27B	-161(2)
1772	C30	C31	C28B	C29B	151(1)
1773	C32	C31	C28B	C36	-30(2)
1774	C32	C31	C28B	H36	-83
1775	C32	C31	C28B	C21B	161.7(8)
1776	C32	C31	C28B	C27B	2(3)
1777	C32	C31	C28B	C29B	-46(1)
1778	C36	C31	C28B	H36	-53
1779	C36	C31	C28B	C21B	-169(2)
1780	C36	C31	C28B	C27B	32(1)
1781	C36	C31	C28B	C29B	-16(2)
1782	C23B	C31	C28B	C36	114(2)
1783	C23B	C31	C28B	H36	61
1784	C23B	C31	C28B	C21B	-54(2)
1785	C23B	C31	C28B	C27B	146(1)
1786	C23B	C31	C28B	C29B	98(2)
1787	C29B	C31	C28B	C36	16(2)
1788	C29B	C31	C28B	H36	-37
1789	C29B	C31	C28B	C21B	-152(1)
1790	C29B	C31	C28B	C27B	48(2)
1791	C30	C31	C29B	C32	90(1)
1792	C30	C31	C29B	C36	-148.9(8)
1793	C30	C31	C29B	C23B	4(1)
1794	C30	C31	C29B	C24B	174(1)
1795	C30	C31	C29B	C28B	-141(1)
1796	C32	C31	C29B	C36	121(1)
1797	C32	C31	C29B	C23B	-85(1)
1798	C32	C31	C29B	C24B	84(2)
1799	C32	C31	C29B	C28B	129(1)
1800	C36	C31	C29B	C32	-121(1)
1801	C36	C31	C29B	C23B	153.2(5)
1802	C36	C31	C29B	C24B	-37(2)
1803	C36	C31	C29B	C28B	8(1)
1804	C23B	C31	C29B	C32	85(1)
1805	C23B	C31	C29B	C36	-153.2(5)
1806	C23B	C31	C29B	C24B	169(2)

1807	C23B	C31	C29B	C28B	-146(1)
1808	C28B	C31	C29B	C32	-129(1)
1809	C28B	C31	C29B	C36	-8(1)
1810	C28B	C31	C29B	C23B	146(1)
1811	C28B	C31	C29B	C24B	-45(2)
1812	C31	C32	N33	C34	0.9(3)
1813	C31	C32	N33	C24B	45.2(9)
1814	C31	C32	N33	H24B	107.7
1815	C31	C32	N33	C25B	3.3(7)
1816	H32	C32	N33	C34	-179.1
1817	H32	C32	N33	C24B	-134.8
1818	H32	C32	N33	H24B	-72.3
1819	H32	C32	N33	C25B	-176.7
1820	C23B	C32	N33	C34	-100.3(6)
1821	C23B	C32	N33	C24B	-56(1)
1822	C23B	C32	N33	H24B	6.6
1823	C23B	C32	N33	C25B	-97.8(9)
1824	C24B	C32	N33	C34	-44.3(9)
1825	C24B	C32	N33	H24B	62.5
1826	C24B	C32	N33	C25B	-42(1)
1827	C29B	C32	N33	C34	-30.8(7)
1828	C29B	C32	N33	C24B	13(1)
1829	C29B	C32	N33	H24B	76
1830	C29B	C32	N33	C25B	-28(1)
1831	C31	C32	C23B	C23	155(2)
1832	C31	C32	C23B	C22B	36.0(6)
1833	C31	C32	C23B	H23C	145
1834	C31	C32	C23B	H23D	-108.1
1835	C31	C32	C23B	C29B	-35.9(7)
1836	H32	C32	C23B	C23	12
1837	H32	C32	C23B	C31	-142.8
1838	H32	C32	C23B	C22B	-106.8
1839	H32	C32	C23B	H23C	2.3
1840	H32	C32	C23B	H23D	109
1841	H32	C32	C23B	C29B	-178.7
1842	N33	C32	C23B	C23	-86(2)
1843	N33	C32	C23B	C31	119.1(5)
1844	N33	C32	C23B	C22B	155.0(5)
1845	N33	C32	C23B	H23C	-95.9
1846	N33	C32	C23B	H23D	11
1847	N33	C32	C23B	C29B	83.1(9)
1848	C24B	C32	C23B	C23	-118(2)
1849	C24B	C32	C23B	C31	87.1(6)
1850	C24B	C32	C23B	C22B	123.1(8)

1851	C24B	C32	C23B	H23C	-127.9
1852	C24B	C32	C23B	H23D	-21
1853	C24B	C32	C23B	C29B	51(1)
1854	C29B	C32	C23B	C23	-169(2)
1855	C29B	C32	C23B	C31	35.9(7)
1856	C29B	C32	C23B	C22B	72(1)
1857	C29B	C32	C23B	H23C	-179
1858	C29B	C32	C23B	H23D	-72
1859	C31	C32	C24B	N33	-143.6(7)
1860	C31	C32	C24B	C34	-76(1)
1861	C31	C32	C24B	H24B	156.4
1862	C31	C32	C24B	C25B	-58(1)
1863	C31	C32	C24B	C29B	23.1(7)
1864	H32	C32	C24B	N33	67
1865	H32	C32	C24B	C34	134.9
1866	H32	C32	C24B	H24B	7
1867	H32	C32	C24B	C25B	152
1868	H32	C32	C24B	C29B	-126.3
1869	N33	C32	C24B	C34	68(1)
1870	N33	C32	C24B	H24B	-60
1871	N33	C32	C24B	C25B	85(2)
1872	N33	C32	C24B	C29B	167(1)
1873	C23B	C32	C24B	N33	146.8(7)
1874	C23B	C32	C24B	C34	-145(1)
1875	C23B	C32	C24B	H24B	86.8
1876	C23B	C32	C24B	C25B	-128(1)
1877	C23B	C32	C24B	C29B	-46.6(9)
1878	C29B	C32	C24B	N33	-167(1)
1879	C29B	C32	C24B	C34	-99(1)
1880	C29B	C32	C24B	H24B	133
1881	C29B	C32	C24B	C25B	-81(2)
1882	C31	C32	C29B	C36	-56.8(9)
1883	C31	C32	C29B	C23B	89(1)
1884	C31	C32	C29B	C24B	-135(1)
1885	C31	C32	C29B	C28B	-22.6(6)
1886	H32	C32	C29B	C31	-88
1887	H32	C32	C29B	C36	-144.6
1888	H32	C32	C29B	C23B	1.4
1889	H32	C32	C29B	C24B	137.7
1890	H32	C32	C29B	C28B	-110
1891	N33	C32	C29B	C31	127.0(9)
1892	N33	C32	C29B	C36	70(1)
1893	N33	C32	C29B	C23B	-143.9(4)

1894	N33	C32	C29B	C24B	-7.6(6)
1895	N33	C32	C29B	C28B	104(1)
1896	C23B	C32	C29B	C31	-89(1)
1897	C23B	C32	C29B	C36	-146(1)
1898	C23B	C32	C29B	C24B	136.3(8)
1899	C23B	C32	C29B	C28B	-112(1)
1900	C24B	C32	C29B	C31	135(1)
1901	C24B	C32	C29B	C36	78(1)
1902	C24B	C32	C29B	C23B	-136.3(8)
1903	C24B	C32	C29B	C28B	112(1)
1904	C32	N33	C34	H34	176.4
1905	C32	N33	C34	C35	-3.6(4)
1906	C32	N33	C34	C24B	51(1)
1907	C32	N33	C34	H25B	169.3
1908	C32	N33	C34	C26B	-22.4(7)
1909	C24B	N33	C34	H34	126
1910	C24B	N33	C34	C35	-54(1)
1911	C24B	N33	C34	H25B	119
1912	C24B	N33	C34	C26B	-73(1)
1913	H24B	N33	C34	H34	69.8
1914	H24B	N33	C34	C35	-110.2
1915	H24B	N33	C34	C24B	-56
1916	H24B	N33	C34	H25B	62.6
1917	H24B	N33	C34	C26B	-129.1
1918	C25B	N33	C34	H34	154
1919	C25B	N33	C34	C35	-26(6)
1920	C25B	N33	C34	C24B	28(6)
1921	C25B	N33	C34	H25B	147
1922	C25B	N33	C34	C26B	-45(6)
1923	C32	N33	C24B	C34	-134.0(7)
1924	C32	N33	C24B	H24B	108.5
1925	C32	N33	C24B	C25B	-131(1)
1926	C32	N33	C24B	C29B	-8.2(7)
1927	C34	N33	C24B	C32	134.0(7)
1928	C34	N33	C24B	H24B	-117.5
1929	C34	N33	C24B	C25B	3.5(7)
1930	C34	N33	C24B	C29B	126(1)
1931	H24B	N33	C24B	C32	-108.5
1932	H24B	N33	C24B	C34	117.5
1933	H24B	N33	C24B	C25B	121
1934	H24B	N33	C24B	C29B	-117
1935	C25B	N33	C24B	C32	131(1)
1936	C25B	N33	C24B	C34	-3.5(7)

1937	C25B	N33	C24B	H24B	-121
1938	C25B	N33	C24B	C29B	122(1)
1939	C32	N33	H24B	C24B	-67.7
1940	C34	N33	H24B	C24B	51.7
1941	C25B	N33	H24B	C24B	46
1942	C32	N33	C25B	H34	180
1943	C32	N33	C25B	C35	-11(2)
1944	C32	N33	C25B	C24B	51(1)
1945	C32	N33	C25B	H25B	175
1946	C32	N33	C25B	C26B	-33(2)
1947	C34	N33	C25B	H34	-21
1948	C34	N33	C25B	C35	148(7)
1949	C34	N33	C25B	C24B	-150(6)
1950	C34	N33	C25B	H25B	-26
1951	C34	N33	C25B	C26B	125(7)
1952	C24B	N33	C25B	H34	129
1953	C24B	N33	C25B	C35	-62(2)
1954	C24B	N33	C25B	H25B	124
1955	C24B	N33	C25B	C26B	-85(2)
1956	H24B	N33	C25B	H34	75
1957	H24B	N33	C25B	C35	-115
1958	H24B	N33	C25B	C24B	-53
1959	H24B	N33	C25B	H25B	71
1960	H24B	N33	C25B	C26B	-138
1961	N33	C34	H34	C25B	-168
1962	C35	C34	H34	C25B	12
1963	C24B	C34	H34	C25B	-129
1964	H25B	C34	H34	C25B	-9
1965	C26B	C34	H34	C25B	26
1966	N33	C34	C35	H35	-177.7
1967	N33	C34	C35	C36	2.3(4)
1968	N33	C34	C35	C25B	158(5)
1969	N33	C34	C35	C26B	-127(1)
1970	N33	C34	C35	C27B	-29.0(7)
1971	H34	C34	C35	H35	2.3
1972	H34	C34	C35	C36	-177.7
1973	H34	C34	C35	C25B	-22
1974	H34	C34	C35	C26B	53
1975	H34	C34	C35	C27B	151
1976	C24B	C34	C35	H35	154.2
1977	C24B	C34	C35	C36	-25.7(6)
1978	C24B	C34	C35	C25B	130(5)
1979	C24B	C34	C35	C26B	-155(2)
1980	C24B	C34	C35	C27B	-57.0(9)
1981	H25B	C34	C35	H35	7.7

1982	H25B	C34	C35	C36	-172.2
1983	H25B	C34	C35	C25B	-17
1984	H25B	C34	C35	C26B	59
1985	H25B	C34	C35	C27B	156.5
1986	C26B	C34	C35	H35	-51
1987	C26B	C34	C35	C36	129(1)
1988	C26B	C34	C35	C25B	-76(5)
1989	C26B	C34	C35	C27B	98(2)
1990	N33	C34	C24B	C32	-63(1)
1991	N33	C34	C24B	H24B	59.8
1992	N33	C34	C24B	C25B	-165(3)
1993	N33	C34	C24B	C29B	-108(2)
1994	H34	C34	C24B	C32	-140.4
1995	H34	C34	C24B	N33	-78
1996	H34	C34	C24B	H24B	-18
1997	H34	C34	C24B	C25B	117
1998	H34	C34	C24B	C29B	174.2
1999	C35	C34	C24B	C32	74(1)
2000	C35	C34	C24B	N33	136.9(8)
2001	C35	C34	C24B	H24B	-163.3
2002	C35	C34	C24B	C25B	-28(3)
2003	C35	C34	C24B	C29B	29(1)
2004	H25B	C34	C24B	C32	-160.5
2005	H25B	C34	C24B	N33	-98
2006	H25B	C34	C24B	H24B	-38
2007	H25B	C34	C24B	C25B	97
2008	H25B	C34	C24B	C29B	154
2009	C26B	C34	C24B	C32	66(1)
2010	C26B	C34	C24B	N33	128.9(8)
2011	C26B	C34	C24B	H24B	-171.3
2012	C26B	C34	C24B	C25B	-36(3)
2013	C26B	C34	C24B	C29B	21(1)
2014	N33	C34	H25B	C25B	-164
2015	H34	C34	H25B	C25B	171
2016	C35	C34	H25B	C25B	9
2017	C24B	C34	H25B	C25B	-115
2018	C26B	C34	H25B	C25B	24
2019	N33	C34	C26B	C35	65(2)
2020	N33	C34	C26B	H35	150
2021	N33	C34	C26B	C25B	142(5)
2022	N33	C34	C26B	H26B	-154.5
2023	N33	C34	C26B	C27B	17(1)
2024	H34	C34	C26B	C35	-132
2025	H34	C34	C26B	H35	-48
2026	H34	C34	C26B	C25B	-56

2027	H34	C34	C26B	H26B	8
2028	H34	C34	C26B	C27B	179.7
2029	C35	C34	C26B	H35	85
2030	C35	C34	C26B	C25B	77(5)
2031	C35	C34	C26B	H26B	140
2032	C35	C34	C26B	C27B	-48(1)
2033	C24B	C34	C26B	C35	28(2)
2034	C24B	C34	C26B	H35	112
2035	C24B	C34	C26B	C25B	104(5)
2036	C24B	C34	C26B	H26B	168
2037	C24B	C34	C26B	C27B	-20(1)
2038	H25B	C34	C26B	C35	-124
2039	H25B	C34	C26B	H35	-39
2040	H25B	C34	C26B	C25B	-47
2041	H25B	C34	C26B	H26B	17
2042	H25B	C34	C26B	C27B	-171.6
2043	C34	H34	C25B	N33	8
2044	C34	H34	C25B	C35	-154
2045	C34	H34	C25B	C24B	30
2046	C34	H34	C25B	H25B	170
2047	C34	H34	C25B	C26B	-139
2048	C34	C35	H35	C26B	96
2049	C36	C35	H35	C26B	-84
2050	C25B	C35	H35	C26B	101
2051	C27B	C35	H35	C26B	-64
2052	C34	C35	C36	C31	1.7(3)
2053	C34	C35	C36	H36	-178.3
2054	C34	C35	C36	C27B	- 137.7(8)
2055	C34	C35	C36	C28B	-8(1)
2056	C34	C35	C36	C29B	14.3(4)
2057	H35	C35	C36	C31	-178.3
2058	H35	C35	C36	H36	1.7
2059	H35	C35	C36	C27B	42.4
2060	H35	C35	C36	C28B	172
2061	H35	C35	C36	C29B	-165.6
2062	C25B	C35	C36	C31	-4(1)
2063	C25B	C35	C36	H36	176
2064	C25B	C35	C36	C27B	-143(1)
2065	C25B	C35	C36	C28B	-14(2)
2066	C25B	C35	C36	C29B	9(1)
2067	C26B	C35	C36	C31	134(1)
2068	C26B	C35	C36	H36	-46
2069	C26B	C35	C36	C27B	-5(2)
2070	C26B	C35	C36	C28B	124(2)

2071	C26B	C35	C36	C29B	147(1)
2072	C27B	C35	C36	C31	139.3(8)
2073	C27B	C35	C36	H36	-40.7
2074	C27B	C35	C36	C28B	129(1)
2075	C27B	C35	C36	C29B	152.0(9)
2076	C34	C35	C25B	N33	-17(4)
2077	C34	C35	C25B	H34	143
2078	C34	C35	C25B	C24B	-41(4)
2079	C34	C35	C25B	H25B	155
2080	C34	C35	C25B	C26B	109(5)
2081	H35	C35	C25B	N33	-174.2
2082	H35	C35	C25B	H34	-14
2083	H35	C35	C25B	C24B	161.4
2084	H35	C35	C25B	H25B	-2
2085	H35	C35	C25B	C26B	-49
2086	C36	C35	C25B	N33	11(2)
2087	C36	C35	C25B	H34	171
2088	C36	C35	C25B	C24B	-13(2)
2089	C36	C35	C25B	H25B	-177
2090	C36	C35	C25B	C26B	137(2)
2091	C26B	C35	C25B	N33	-126(2)
2092	C26B	C35	C25B	H34	34
2093	C26B	C35	C25B	C24B	-150(2)
2094	C26B	C35	C25B	H25B	46
2095	C27B	C35	C25B	N33	-25(3)
2096	C27B	C35	C25B	H34	135
2097	C27B	C35	C25B	C24B	-50(2)
2098	C27B	C35	C25B	H25B	147
2099	C27B	C35	C25B	C26B	100(2)
2100	C34	C35	C26B	H35	-109
2101	C34	C35	C26B	C25B	-11(1)
2102	C34	C35	C26B	H26B	-88
2103	C34	C35	C26B	C27B	137.3(6)
2104	H35	C35	C26B	C34	109
2105	H35	C35	C26B	C25B	98
2106	H35	C35	C26B	H26B	21
2107	H35	C35	C26B	C27B	-113
2108	C36	C35	C26B	C34	-134(1)
2109	C36	C35	C26B	H35	116
2110	C36	C35	C26B	C25B	-146(1)
2111	C36	C35	C26B	H26B	137
2112	C36	C35	C26B	C27B	2.8(8)
2113	C25B	C35	C26B	C34	11(1)
2114	C25B	C35	C26B	H35	-98
2115	C25B	C35	C26B	H26B	-77

2116	C25B	C35	C26B	C27B	148(1)
2117	C27B	C35	C26B	C34	-137.3(6)
2118	C27B	C35	C26B	H35	113
2119	C27B	C35	C26B	C25B	-148(1)
2120	C27B	C35	C26B	H26B	134
2121	C34	C35	C27B	C36	62(1)
2122	C34	C35	C27B	H36	114
2123	C34	C35	C27B	C26B	-112(1)
2124	C34	C35	C27B	H27B	-147
2125	C34	C35	C27B	C28B	45.6(9)
2126	H35	C35	C27B	C36	-144.3
2127	H35	C35	C27B	H36	-93
2128	H35	C35	C27B	C26B	41
2129	H35	C35	C27B	H27B	6
2130	H35	C35	C27B	C28B	-161.1
2131	C36	C35	C27B	H36	51.3
2132	C36	C35	C27B	C26B	-175(2)
2133	C36	C35	C27B	H27B	150
2134	C36	C35	C27B	C28B	-16.8(5)
2135	C25B	C35	C27B	C36	65(2)
2136	C25B	C35	C27B	H36	116
2137	C25B	C35	C27B	C26B	-110(2)
2138	C25B	C35	C27B	H27B	-145
2139	C25B	C35	C27B	C28B	48(2)
2140	C26B	C35	C27B	C36	175(2)
2141	C26B	C35	C27B	H36	-134
2142	C26B	C35	C27B	H27B	-35
2143	C26B	C35	C27B	C28B	158(1)
2144	C35	H35	C26B	C34	-49
2145	C35	H35	C26B	C25B	-47
2146	C35	H35	C26B	H26B	-166
2147	C35	H35	C26B	C27B	85
2148	C31	C36	H36	C27B	-132.1
2149	C31	C36	H36	C28B	-6.3
2150	C35	C36	H36	C27B	48
2151	C35	C36	H36	C28B	173.8
2152	C27B	C36	H36	C28B	126
2153	C28B	C36	H36	C27B	-126
2154	C29B	C36	H36	C27B	-153
2155	C29B	C36	H36	C28B	-27.5
2156	C31	C36	C27B	C35	-119.2(9)
2157	C31	C36	C27B	H36	101.9
2158	C31	C36	C27B	C26B	-117(1)

2159	C31	C36	C27B	H27B	89
2160	C31	C36	C27B	C28B	24.7(9)
2161	C35	C36	C27B	H36	-138.9
2162	C35	C36	C27B	C26B	2.0(6)
2163	C35	C36	C27B	H27B	-152
2164	C35	C36	C27B	C28B	143.9(9)
2165	H36	C36	C27B	C35	138.9
2166	H36	C36	C27B	C26B	141
2167	H36	C36	C27B	H27B	-13
2168	H36	C36	C27B	C28B	-77
2169	C28B	C36	C27B	C35	-143.9(9)
2170	C28B	C36	C27B	H36	77
2171	C28B	C36	C27B	C26B	-142(1)
2172	C28B	C36	C27B	H27B	64
2173	C29B	C36	C27B	C35	-75(2)
2174	C29B	C36	C27B	H36	146
2175	C29B	C36	C27B	C26B	-73(2)
2176	C29B	C36	C27B	H27B	133
2177	C29B	C36	C27B	C28B	69(2)
2178	C31	C36	C28B	H36	166
2179	C31	C36	C28B	C21B	160(3)
2180	C31	C36	C28B	C27B	-136(2)
2181	C31	C36	C28B	C29B	8(1)
2182	C35	C36	C28B	C31	22(2)
2183	C35	C36	C28B	H36	-171
2184	C35	C36	C28B	C21B	-177.5(9)
2185	C35	C36	C28B	C27B	-114(1)
2186	C35	C36	C28B	C29B	30(1)
2187	H36	C36	C28B	C31	-166
2188	H36	C36	C28B	C21B	-6
2189	H36	C36	C28B	C27B	57
2190	H36	C36	C28B	C29B	-158.8
2191	C27B	C36	C28B	C31	136(2)
2192	C27B	C36	C28B	H36	-57
2193	C27B	C36	C28B	C21B	-63(2)
2194	C27B	C36	C28B	C29B	144(1)
2195	C29B	C36	C28B	C31	-8(1)
2196	C29B	C36	C28B	H36	158.8
2197	C29B	C36	C28B	C21B	153(2)
2198	C29B	C36	C28B	C27B	-144(1)
2199	C31	C36	C29B	C32	90(1)
2200	C31	C36	C29B	C23B	-39.1(7)
2201	C31	C36	C29B	C24B	156(1)

2202	C31	C36	C29B	C28B	-6.5(9)
2203	C35	C36	C29B	C31	-155.2(6)
2204	C35	C36	C29B	C32	-65(1)
2205	C35	C36	C29B	C23B	166(1)
2206	C35	C36	C29B	C24B	0.7(7)
2207	C35	C36	C29B	C28B	-161.8(9)
2208	H36	C36	C29B	C31	43
2209	H36	C36	C29B	C32	132.7
2210	H36	C36	C29B	C23B	4
2211	H36	C36	C29B	C24B	-161.1
2212	H36	C36	C29B	C28B	36
2213	C27B	C36	C29B	C31	-84(2)
2214	C27B	C36	C29B	C32	5(2)
2215	C27B	C36	C29B	C23B	-124(2)
2216	C27B	C36	C29B	C24B	71(2)
2217	C27B	C36	C29B	C28B	-91(2)
2218	C28B	C36	C29B	C31	6.5(9)
2219	C28B	C36	C29B	C32	96(1)
2220	C28B	C36	C29B	C23B	-33(1)
2221	C28B	C36	C29B	C24B	162(1)
2222	C36	H36	C27B	C35	-51.1
2223	C36	H36	C27B	C26B	-76
2224	C36	H36	C27B	H27B	171
2225	C36	H36	C27B	C28B	30.8
2226	C28B	H36	C27B	C35	-82
2227	C28B	H36	C27B	C36	-30.8
2228	C28B	H36	C27B	C26B	-107
2229	C28B	H36	C27B	H27B	140.1
2230	C36	H36	C28B	C31	63
2231	C36	H36	C28B	C21B	176
2232	C36	H36	C28B	C27B	-39.1
2233	C36	H36	C28B	C29B	35
2234	C27B	H36	C28B	C31	102
2235	C27B	H36	C28B	C36	39.1
2236	C27B	H36	C28B	C21B	-144.5
2237	C27B	H36	C28B	C29B	74
2238	C17	S1	O5	U1	139.9(1)
2239	C17	S1	O5	U1B	141.6(1)
2240	C18	S1	O5	U1	-118.3(1)
2241	C18	S1	O5	U1B	-116.7(1)
2242	O5	S1	C17	H17A	172.8

2243	O5	S1	C17	H17B	-67.2
2244	O5	S1	C17	H17C	52.8
2245	C18	S1	C17	H17A	65.8
2246	C18	S1	C17	H17B	-174.2
2247	C18	S1	C17	H17C	-54.2
2248	O5	S1	C18	H18A	-52.2
2249	O5	S1	C18	H18B	67.9
2250	O5	S1	C18	H18C	-172.2
2251	C17	S1	C18	H18A	54.9
2252	C17	S1	C18	H18B	174.9
2253	C17	S1	C18	H18C	-65.1
2254	U1	O5	U1B	O3	119(1)
2255	U1	O5	U1B	O4	-52(1)
2256	U1	O5	U1B	O1	-151(1)
2257	U1	O5	U1B	O2	-128(1)
2258	U1	O5	U1B	O21	23(1)
2259	U1	O5	U1B	O22	31(1)
2260	U1	O5	U1B	O21B	45(1)
2261	U1	O5	U1B	O22B	25(1)
2262	S1	O5	U1B	O3	-39.1(2)
2263	S1	O5	U1B	O4	149.1(1)
2264	S1	O5	U1B	O1	50.1(1)
2265	S1	O5	U1B	O2	73.6(3)
2266	S1	O5	U1B	O21	-135.2(2)
2267	S1	O5	U1B	O22	-128.0(1)
2268	S1	O5	U1B	O21B	-113.8(3)
2269	S1	O5	U1B	O22B	-133.4(6)
2270	C10	C11B	C12B	C11	148(26)
2271	C10	C11B	C12B	C15	-173(3)
2272	C10	C11B	C12B	H16	-12
2273	C10	C11B	C12B	H12B	0
2274	C10	C11B	C12B	N13B	180(3)
2275	C12	C11B	C12B	C11	-21(21)
2276	C12	C11B	C12B	C15	18(4)
2277	C12	C11B	C12B	H16	178
2278	C12	C11B	C12B	H12B	-170
2279	C12	C11B	C12B	N13B	10(4)
2280	C16	C11B	C12B	C11	-179(24)
2281	C16	C11B	C12B	C15	-139(7)
2282	C16	C11B	C12B	H16	21
2283	C16	C11B	C12B	H12B	33

2284	C16	C11B	C12B	N13B	-147(7)
2285	C16B	C11B	C12B	C11	-24(21)
2286	C16B	C11B	C12B	C15	15(4)
2287	C16B	C11B	C12B	H16	176
2288	C16B	C11B	C12B	H12B	-172
2289	C16B	C11B	C12B	N13B	8(4)
2290	C10	C11B	C16B	C11	-9(9)
2291	C10	C11B	C16B	H12	-10
2292	C10	C11B	C16B	N13	-176(2)
2293	C10	C11B	C16B	C15B	176(3)
2294	C10	C11B	C16B	H16B	-3
2295	C12	C11B	C16B	C11	142(18)
2296	C12	C11B	C16B	H12	142
2297	C12	C11B	C16B	N13	-25(12)
2298	C12	C11B	C16B	C15B	-33(12)
2299	C12	C11B	C16B	H16B	148
2300	C16	C11B	C16B	C11	172(13)
2301	C16	C11B	C16B	H12	171
2302	C16	C11B	C16B	N13	4(5)
2303	C16	C11B	C16B	C15B	-3(5)
2304	C16	C11B	C16B	H16B	178
2305	C12B	C11B	C16B	C11	166(12)
2306	C12B	C11B	C16B	H12	166
2307	C12B	C11B	C16B	N13	-1(4)
2308	C12B	C11B	C16B	C15B	-9(5)
2309	C12B	C11B	C16B	H16B	172
2310	C11	C12B	H12B	C16	-24
2311	C15	C12B	H12B	C16	151
2312	H16	C12B	H12B	C16	6
2313	C11B	C12B	H12B	C16	-22
2314	N13B	C12B	H12B	C16	158
2315	C11	C12B	N13B	C14	-3(3)
2316	C11	C12B	N13B	H15	165
2317	C11	C12B	N13B	C16	54(9)
2318	C11	C12B	N13B	C14B	-4(4)
2319	C15	C12B	N13B	C14	-111(11)
2320	C15	C12B	N13B	H15	58
2321	C15	C12B	N13B	C16	-54(15)
2322	C15	C12B	N13B	C14B	-112(11)
2323	H16	C12B	N13B	C14	-166
2324	H16	C12B	N13B	H15	2
2325	H16	C12B	N13B	C16	-110
2326	H16	C12B	N13B	C14B	-167
2327	C11B	C12B	N13B	C14	-5(4)
2328	C11B	C12B	N13B	H15	163

2329	C11B	C12B	N13B	C16	52(9)
2330	C11B	C12B	N13B	C14B	-6(5)
2331	H12B	C12B	N13B	C14	175
2332	H12B	C12B	N13B	H15	-17
2333	H12B	C12B	N13B	C16	-128
2334	H12B	C12B	N13B	C14B	174
2335	C14	N13B	C14B	N13	-109(130)
2336	C14	N13B	C14B	H14	73
2337	C14	N13B	C14B	C15	-77(129)
2338	C14	N13B	C14B	H14B	79
2339	C14	N13B	C14B	C15B	-100(129)
2340	H15	N13B	C14B	N13	-166
2341	H15	N13B	C14B	H14	16
2342	H15	N13B	C14B	C15	-133
2343	H15	N13B	C14B	H14B	22
2344	H15	N13B	C14B	C15B	-157
2345	C16	N13B	C14B	N13	-8(7)
2346	C16	N13B	C14B	H14	175
2347	C16	N13B	C14B	C15	25(6)
2348	C16	N13B	C14B	H14B	-179
2349	C16	N13B	C14B	C15B	2(6)
2350	C12B	N13B	C14B	N13	-3(7)
2351	C12B	N13B	C14B	H14	179
2352	C12B	N13B	C14B	C15	30(6)
2353	C12B	N13B	C14B	H14B	-174
2354	C12B	N13B	C14B	C15B	7(7)
2355	N13	C14B	H14B	C14	175
2356	H14	C14B	H14B	C14	10
2357	C15	C14B	H14B	C14	-8
2358	N13B	C14B	H14B	C14	-12
2359	C15B	C14B	H14B	C14	167
2360	N13	C14B	C15B	C12	106(11)
2361	N13	C14B	C15B	C14	133(37)
2362	N13	C14B	C15B	H15B	-68
2363	N13	C14B	C15B	C16B	111(11)
2364	H14	C14B	C15B	C12	176
2365	H14	C14B	C15B	C14	-157
2366	H14	C14B	C15B	H15B	2
2367	H14	C14B	C15B	C16B	-179
2368	C15	C14B	C15B	C12	-17(6)
2369	C15	C14B	C15B	C14	10(31)
2370	C15	C14B	C15B	H15B	169
2371	C15	C14B	C15B	C16B	-12(6)

2372	N13B	C14B	C15B	C12	-13(7)
2373	N13B	C14B	C15B	C14	13(31)
2374	N13B	C14B	C15B	H15B	173
2375	N13B	C14B	C15B	C16B	-8(7)
2376	H14B	C14B	C15B	C12	168
2377	H14B	C14B	C15B	C14	-166
2378	H14B	C14B	C15B	H15B	-6
2379	H14B	C14B	C15B	C16B	173
2380	C12	C15B	H15B	N13	-125
2381	C14	C15B	H15B	N13	51
2382	C14B	C15B	H15B	N13	49
2383	C16B	C15B	H15B	N13	-130
2384	C12	C15B	C16B	C11	127(18)
2385	C12	C15B	C16B	H12	-49
2386	C12	C15B	C16B	N13	54(19)
2387	C12	C15B	C16B	C11B	128(18)
2388	C12	C15B	C16B	H16B	-53
2389	C14	C15B	C16B	C11	7(5)
2390	C14	C15B	C16B	H12	-169
2391	C14	C15B	C16B	N13	-66(11)
2392	C14	C15B	C16B	C11B	7(4)
2393	C14	C15B	C16B	H16B	-173
2394	C14B	C15B	C16B	C11	8(6)
2395	C14B	C15B	C16B	H12	-167
2396	C14B	C15B	C16B	N13	-65(11)
2397	C14B	C15B	C16B	C11B	9(5)
2398	C14B	C15B	C16B	H16B	-172
2399	H15B	C15B	C16B	C11	-173
2400	H15B	C15B	C16B	H12	12
2401	H15B	C15B	C16B	N13	115
2402	H15B	C15B	C16B	C11B	-172
2403	H15B	C15B	C16B	H16B	7
2404	C11	C16B	H16B	C12	-156
2405	H12	C16B	H16B	C12	11
2406	N13	C16B	H16B	C12	16
2407	C11B	C16B	H16B	C12	-157
2408	C15B	C16B	H16B	C12	24
2409	O3	U1B	O21B	U1	164(2)
2410	O3	U1B	O21B	O22	147.0(9)
2411	O3	U1B	O21B	C21B	140.2(8)
2412	O4	U1B	O21B	U1	-15(2)
2413	O4	U1B	O21B	O22	-32.3(8)
2414	O4	U1B	O21B	C21B	-39.1(8)
2415	O1	U1B	O21B	U1	-118(2)

2416	O1	U1B	O21B	O22	-135.1(7)
2417	O1	U1B	O21B	C21B	-141.9(7)
2418	O2	U1B	O21B	U1	76(2)
2419	O2	U1B	O21B	O22	59(1)
2420	O2	U1B	O21B	C21B	52(1)
2421	O21	U1B	O21B	U1	72(2)
2422	O21	U1B	O21B	O22	54.4(8)
2423	O21	U1B	O21B	C21B	47.7(8)
2424	O22	U1B	O21B	U1	17(2)
2425	O22	U1B	O21B	C21B	-6.8(6)
2426	O5	U1B	O21B	U1	-95(2)
2427	O5	U1B	O21B	O22	-112.2(9)
2428	O5	U1B	O21B	C21B	-118.9(8)
2429	O22B	U1B	O21B	U1	70(2)
2430	O22B	U1B	O21B	O22	52.8(9)
2431	O22B	U1B	O21B	C21B	46.1(9)
2432	O3	U1B	O22B	U1	-125(1)
2433	O3	U1B	O22B	C21	-139(2)
2434	O3	U1B	O22B	C30B	-120(1)
2435	O4	U1B	O22B	U1	48(1)
2436	O4	U1B	O22B	C21	33(2)
2437	O4	U1B	O22B	C30B	52(1)
2438	O1	U1B	O22B	U1	145(2)
2439	O1	U1B	O22B	C21	130(2)
2440	O1	U1B	O22B	C30B	149(1)
2441	O2	U1B	O22B	U1	136(1)
2442	O2	U1B	O22B	C21	121(2)
2443	O2	U1B	O22B	C30B	140(2)
2444	O21	U1B	O22B	U1	144(3)
2445	O21	U1B	O22B	C21	129(5)
2446	O21	U1B	O22B	C30B	148(4)
2447	O22	U1B	O22B	U1	-34(1)
2448	O22	U1B	O22B	C21	-49(2)
2449	O22	U1B	O22B	C30B	-30(1)
2450	O5	U1B	O22B	U1	-28(1)
2451	O5	U1B	O22B	C21	-43(3)
2452	O5	U1B	O22B	C30B	-24(2)
2453	O21B	U1B	O22B	U1	-47(1)
2454	O21B	U1B	O22B	C21	-62(2)
2455	O21B	U1B	O22B	C30B	-43(1)
2456	U1	O21B	C21B	O22	10.6(9)

2457	U1	O21B	C21B	C30	-20(2)
2458	U1	O21B	C21B	C22B	-41(1)
2459	U1	O21B	C21B	C28B	138.1(8)
2460	O22	O21B	C21B	C30	-30(2)
2461	O22	O21B	C21B	C22B	-52(1)
2462	O22	O21B	C21B	C28B	128(1)
2463	U1B	O21B	C21B	O22	11.7(9)
2464	U1B	O21B	C21B	C30	-19(2)
2465	U1B	O21B	C21B	C22B	-40(1)
2466	U1B	O21B	C21B	C28B	139.2(7)
2467	U1	O22B	C30B	O21	-134(5)
2468	U1	O22B	C30B	C22	20(3)
2469	U1	O22B	C30B	C28	-160(1)
2470	U1	O22B	C30B	C22B	24(2)
2471	U1	O22B	C30B	C31B	-158(1)
2472	C21	O22B	C30B	O21	19(4)
2473	C21	O22B	C30B	C22	172(3)
2474	C21	O22B	C30B	C28	-7(1)
2475	C21	O22B	C30B	C22B	177(2)
2476	C21	O22B	C30B	C31B	-5(1)
2477	U1B	O22B	C30B	O21	-135(5)
2478	U1B	O22B	C30B	C22	19(3)
2479	U1B	O22B	C30B	C28	-160(1)
2480	U1B	O22B	C30B	C22B	24(2)
2481	U1B	O22B	C30B	C31B	-158(1)
2482	O22	C21B	C22B	C22	-30(3)
2483	O22	C21B	C22B	C30	107(2)
2484	O22	C21B	C22B	C23B	160.4(9)
2485	O22	C21B	C22B	C30B	-24(1)
2486	C30	C21B	C22B	C22	-138(4)
2487	C30	C21B	C22B	C23B	53(2)
2488	C30	C21B	C22B	C30B	-132(2)
2489	O21B	C21B	C22B	C22	-9(4)
2490	O21B	C21B	C22B	C30	128(2)
2491	O21B	C21B	C22B	C23B	-178(1)
2492	O21B	C21B	C22B	C30B	-3(2)
2493	C28B	C21B	C22B	C22	171(3)
2494	C28B	C21B	C22B	C30	-51(2)
2495	C28B	C21B	C22B	C23B	2(1)
2496	C28B	C21B	C22B	C30B	177.6(9)
2497	O22	C21B	C28B	C31	-135(1)
2498	O22	C21B	C28B	C36	61(2)
2499	O22	C21B	C28B	H36	56
2500	O22	C21B	C28B	C27B	31(2)
2501	O22	C21B	C28B	C29B	-148(1)

2502	C30	C21B	C28B	C31	3(1)
2503	C30	C21B	C28B	C36	-162(2)
2504	C30	C21B	C28B	H36	-166.5
2505	C30	C21B	C28B	C27B	168(1)
2506	C30	C21B	C28B	C29B	-10(1)
2507	O21B	C21B	C28B	C31	-163(1)
2508	O21B	C21B	C28B	C36	32(2)
2509	O21B	C21B	C28B	H36	28
2510	O21B	C21B	C28B	C27B	2(2)
2511	O21B	C21B	C28B	C29B	-176.0(9)
2512	C22B	C21B	C28B	C31	16(1)
2513	C22B	C21B	C28B	C36	-148(2)
2514	C22B	C21B	C28B	H36	-152.7
2515	C22B	C21B	C28B	C27B	-178.4(9)
2516	C22B	C21B	C28B	C29B	3(1)
2517	C22	C22B	C23B	C23	-12.5(9)
2518	C22	C22B	C23B	C31	175(1)
2519	C22	C22B	C23B	C32	148.2(9)
2520	C22	C22B	C23B	H23C	59
2521	C22	C22B	C23B	H23D	-63
2522	C22	C22B	C23B	C29B	178(1)
2523	C30	C22B	C23B	C23	174.9(8)
2524	C30	C22B	C23B	C31	2.8(7)
2525	C30	C22B	C23B	C32	-24(1)
2526	C30	C22B	C23B	H23C	-114
2527	C30	C22B	C23B	H23D	124
2528	C30	C22B	C23B	C29B	5(1)
2529	C21B	C22B	C23B	C23	163.0(7)
2530	C21B	C22B	C23B	C31	-9.1(7)
2531	C21B	C22B	C23B	C32	-36(1)
2532	C21B	C22B	C23B	H23C	-125.7
2533	C21B	C22B	C23B	H23D	112.6
2534	C21B	C22B	C23B	C29B	-7(1)
2535	C30B	C22B	C23B	C23	-12(1)
2536	C30B	C22B	C23B	C31	176(1)
2537	C30B	C22B	C23B	C32	149.0(9)
2538	C30B	C22B	C23B	H23C	59
2539	C30B	C22B	C23B	H23D	-62
2540	C30B	C22B	C23B	C29B	179(1)
2541	C22	C22B	C30B	O21	-167(2)
2542	C22	C22B	C30B	C28	14(2)
2543	C22	C22B	C30B	O22B	-172(2)
2544	C22	C22B	C30B	C31B	10(2)

2545	C30	C22B	C30B	O21	3(2)
2546	C30	C22B	C30B	C22	170(2)
2547	C30	C22B	C30B	C28	-176(1)
2548	C30	C22B	C30B	O22B	-2(2)
2549	C30	C22B	C30B	C31B	-180(1)
2550	C21B	C22B	C30B	O21	17(2)
2551	C21B	C22B	C30B	C22	-176(2)
2552	C21B	C22B	C30B	C28	-161(1)
2553	C21B	C22B	C30B	O22B	13(2)
2554	C21B	C22B	C30B	C31B	-165(1)
2555	C23B	C22B	C30B	O21	-168.7(8)
2556	C23B	C22B	C30B	C22	-1(1)
2557	C23B	C22B	C30B	C28	13(2)
2558	C23B	C22B	C30B	O22B	-173(1)
2559	C23B	C22B	C30B	C31B	9(2)
2560	C31	C23B	H23D	C23	144.9
2561	C32	C23B	H23D	C23	-155.2
2562	C22B	C23B	H23D	C23	59.3
2563	H23C	C23B	H23D	C23	-63.5
2564	C29B	C23B	H23D	C23	173.6
2565	C23	C23B	C29B	C31	-91(5)
2566	C23	C23B	C29B	C32	156(4)
2567	C23	C23B	C29B	C36	-62(5)
2568	C23	C23B	C29B	C24B	99(5)
2569	C23	C23B	C29B	C28B	-77(5)
2570	C31	C23B	C29B	C32	-113(1)
2571	C31	C23B	C29B	C36	29.0(5)
2572	C31	C23B	C29B	C24B	-171(2)
2573	C31	C23B	C29B	C28B	13.8(5)
2574	C32	C23B	C29B	C31	113(1)
2575	C32	C23B	C29B	C36	142(1)
2576	C32	C23B	C29B	C24B	-58(1)
2577	C32	C23B	C29B	C28B	127(1)
2578	C22B	C23B	C29B	C31	-5(1)
2579	C22B	C23B	C29B	C32	-118.1(8)
2580	C22B	C23B	C29B	C36	24(1)
2581	C22B	C23B	C29B	C24B	-176(1)
2582	C22B	C23B	C29B	C28B	9(1)
2583	H23C	C23B	C29B	C31	114
2584	H23C	C23B	C29B	C32	1
2585	H23C	C23B	C29B	C36	143.1
2586	H23C	C23B	C29B	C24B	-57
2587	H23C	C23B	C29B	C28B	127.9

2588	H23D	C23B	C29B	C31	-124
2589	H23D	C23B	C29B	C32	122.7
2590	H23D	C23B	C29B	C36	-95
2591	H23D	C23B	C29B	C24B	65
2592	H23D	C23B	C29B	C28B	-110
2593	C32	C24B	H24B	N33	66.4
2594	C34	C24B	H24B	N33	-72.4
2595	C25B	C24B	H24B	N33	-84
2596	C29B	C24B	H24B	N33	96
2597	C32	C24B	C25B	N33	-74(1)
2598	C32	C24B	C25B	H34	-129
2599	C32	C24B	C25B	C35	53(2)
2600	C32	C24B	C25B	H25B	-140
2601	C32	C24B	C25B	C26B	41(2)
2602	N33	C24B	C25B	H34	-55
2603	N33	C24B	C25B	C35	127(1)
2604	N33	C24B	C25B	H25B	-66
2605	N33	C24B	C25B	C26B	115(2)
2606	C34	C24B	C25B	N33	15(3)
2607	C34	C24B	C25B	H34	-40
2608	C34	C24B	C25B	C35	142(4)
2609	C34	C24B	C25B	H25B	-51
2610	C34	C24B	C25B	C26B	130(4)
2611	H24B	C24B	C25B	N33	65
2612	H24B	C24B	C25B	H34	10
2613	H24B	C24B	C25B	C35	-168
2614	H24B	C24B	C25B	H25B	-1
2615	H24B	C24B	C25B	C26B	180
2616	C29B	C24B	C25B	N33	-115(2)
2617	C29B	C24B	C25B	H34	-170
2618	C29B	C24B	C25B	C35	12(2)
2619	C29B	C24B	C25B	H25B	179
2620	C29B	C24B	C25B	C26B	-0(2)
2621	C32	C24B	C29B	C31	-102(2)
2622	C32	C24B	C29B	C36	-<br>129.7(8)
2623	C32	C24B	C29B	C23B	64(1)
2624	C32	C24B	C29B	C28B	-121(1)
2625	N33	C24B	C29B	C31	-88(2)
2626	N33	C24B	C29B	C32	13(1)
2627	N33	C24B	C29B	C36	-116(1)
2628	N33	C24B	C29B	C23B	78(2)
2629	N33	C24B	C29B	C28B	-107(1)
2630	C34	C24B	C29B	C31	8(2)
2631	C34	C24B	C29B	C32	110(1)

2632	C34	C24B	C29B	C36	-20(1)
2633	C34	C24B	C29B	C23B	174(1)
2634	C34	C24B	C29B	C28B	-11(2)
2635	H24B	C24B	C29B	C31	-159
2636	H24B	C24B	C29B	C32	-57
2637	H24B	C24B	C29B	C36	173
2638	H24B	C24B	C29B	C23B	7
2639	H24B	C24B	C29B	C28B	-178
2640	C25B	C24B	C29B	C31	21(2)
2641	C25B	C24B	C29B	C32	123(1)
2642	C25B	C24B	C29B	C36	-7(1)
2643	C25B	C24B	C29B	C23B	-173(1)
2644	C25B	C24B	C29B	C28B	2(2)
2645	N33	C25B	H25B	C34	9
2646	H34	C25B	H25B	C34	-9
2647	C35	C25B	H25B	C34	-163
2648	C24B	C25B	H25B	C34	35
2649	C26B	C25B	H25B	C34	-146
2650	N33	C25B	C26B	C34	-28(4)
2651	N33	C25B	C26B	C35	68(2)
2652	N33	C25B	C26B	H35	159
2653	N33	C25B	C26B	H26B	-149
2654	N33	C25B	C26B	C27B	31(2)
2655	H34	C25B	C26B	C34	107
2656	H34	C25B	C26B	C35	-158
2657	H34	C25B	C26B	H35	-66
2658	H34	C25B	C26B	H26B	-14
2659	H34	C25B	C26B	C27B	166
2660	C35	C25B	C26B	C34	-96(5)
2661	C35	C25B	C26B	H35	92
2662	C35	C25B	C26B	H26B	143
2663	C35	C25B	C26B	C27B	-37(1)
2664	C24B	C25B	C26B	C34	-61(5)
2665	C24B	C25B	C26B	C35	35(2)
2666	C24B	C25B	C26B	H35	127
2667	C24B	C25B	C26B	H26B	179
2668	C24B	C25B	C26B	C27B	-2(2)
2669	H25B	C25B	C26B	C34	120
2670	H25B	C25B	C26B	C35	-144
2671	H25B	C25B	C26B	H35	-52
2672	H25B	C25B	C26B	H26B	-1
2673	H25B	C25B	C26B	C27B	179
2674	C34	C26B	C27B	C35	33.9(8)
2675	C34	C26B	C27B	C36	29(1)
2676	C34	C26B	C27B	H36	98

2677	C34	C26B	C27B	H27B	-170.8
2678	C34	C26B	C27B	C28B	9(1)
2679	C35	C26B	C27B	C36	-5(1)
2680	C35	C26B	C27B	H36	64
2681	C35	C26B	C27B	H27B	155
2682	C35	C26B	C27B	C28B	-25(2)
2683	H35	C26B	C27B	C35	-97
2684	H35	C26B	C27B	C36	-102
2685	H35	C26B	C27B	H36	-33
2686	H35	C26B	C27B	H27B	58
2687	H35	C26B	C27B	C28B	-122
2688	C25B	C26B	C27B	C35	26(1)
2689	C25B	C26B	C27B	C36	21(2)
2690	C25B	C26B	C27B	H36	90
2691	C25B	C26B	C27B	H27B	-179
2692	C25B	C26B	C27B	C28B	1(2)
2693	H26B	C26B	C27B	C35	-154
2694	H26B	C26B	C27B	C36	-159
2695	H26B	C26B	C27B	H36	-90
2696	H26B	C26B	C27B	H27B	1
2697	H26B	C26B	C27B	C28B	-179
2698	C35	C27B	C28B	C31	-33(2)
2699	C35	C27B	C28B	C36	34.4(9)
2700	C35	C27B	C28B	H36	126.8
2701	C35	C27B	C28B	C21B	173.1(8)
2702	C35	C27B	C28B	C29B	-9(1)
2703	C36	C27B	C28B	C31	-67(2)
2704	C36	C27B	C28B	H36	92
2705	C36	C27B	C28B	C21B	139(2)
2706	C36	C27B	C28B	C29B	-43(1)
2707	H36	C27B	C28B	C31	-160
2708	H36	C27B	C28B	C36	-92
2709	H36	C27B	C28B	C21B	46
2710	H36	C27B	C28B	C29B	-136
2711	C26B	C27B	C28B	C31	-24(2)
2712	C26B	C27B	C28B	C36	44(1)
2713	C26B	C27B	C28B	H36	136
2714	C26B	C27B	C28B	C21B	-178(1)
2715	C26B	C27B	C28B	C29B	0(1)
2716	H27B	C27B	C28B	C31	156
2717	H27B	C27B	C28B	C36	-136
2718	H27B	C27B	C28B	H36	-44
2719	H27B	C27B	C28B	C21B	2
2720	H27B	C27B	C28B	C29B	-179.5
2721	C31	C28B	C29B	C32	62(1)

2722	C31	C28B	C29B	C36	167(2)
2723	C31	C28B	C29B	C23B	-37(1)
2724	C31	C28B	C29B	C24B	147(2)
2725	C36	C28B	C29B	C31	-167(2)
2726	C36	C28B	C29B	C32	-105(1)
2727	C36	C28B	C29B	C23B	155.8(9)
2728	C36	C28B	C29B	C24B	-20(1)
2729	H36	C28B	C29B	C31	168
2730	H36	C28B	C29B	C32	-129
2731	H36	C28B	C29B	C36	-24.6
2732	H36	C28B	C29B	C23B	131
2733	H36	C28B	C29B	C24B	-45
2734	C21B	C28B	C29B	C31	29(1)
2735	C21B	C28B	C29B	C32	92(1)
2736	C21B	C28B	C29B	C36	-164(1)
2737	C21B	C28B	C29B	C23B	-8(1)
2738	C21B	C28B	C29B	C24B	176.2(9)
2739	C27B	C28B	C29B	C31	-149(2)
2740	C27B	C28B	C29B	C32	-87(1)
2741	C27B	C28B	C29B	C36	18.0(6)
2742	C27B	C28B	C29B	C23B	173.9(9)
2743	C27B	C28B	C29B	C24B	-2(1)
2744	O21	C30B	C31B	C21	0.7(9)
2745	O21	C30B	C31B	C27	-7(2)
2746	O21	C30B	C31B	C29	-176(1)
2747	O21	C30B	C31B	C32B	-175(2)
2748	O21	C30B	C31B	C36B	3(1)
2749	C22	C30B	C31B	C21	-174(2)
2750	C22	C30B	C31B	C27	179(1)
2751	C22	C30B	C31B	C29	10(2)
2752	C22	C30B	C31B	C32B	11(2)
2753	C22	C30B	C31B	C36B	-172(1)
2754	C28	C30B	C31B	C21	18(3)
2755	C28	C30B	C31B	C27	10(2)
2756	C28	C30B	C31B	C29	-159(4)
2757	C28	C30B	C31B	C32B	-158(5)
2758	C28	C30B	C31B	C36B	19(2)
2759	O22B	C30B	C31B	C21	4(1)
2760	O22B	C30B	C31B	C27	-3(2)
2761	O22B	C30B	C31B	C29	-172(1)
2762	O22B	C30B	C31B	C32B	-171(2)
2763	O22B	C30B	C31B	C36B	6(2)
2764	C22B	C30B	C31B	C21	-177(2)
2765	C22B	C30B	C31B	C27	175(1)
2766	C22B	C30B	C31B	C29	6(2)

2767	C22B	C30B	C31B	C32B	7(3)
2768	C22B	C30B	C31B	C36B	-175(1)
2769	C21	C31B	C32B	C23	-10(3)
2770	C21	C31B	C32B	C24	177(3)
2771	C21	C31B	C32B	C28	160(5)
2772	C21	C31B	C32B	H32B	-5
2773	C21	C31B	C32B	N33B	175(2)
2774	C27	C31B	C32B	C23	-178.7(9)
2775	C27	C31B	C32B	C24	8(4)
2776	C27	C31B	C32B	C28	-9(2)
2777	C27	C31B	C32B	H32B	-174
2778	C27	C31B	C32B	N33B	6(3)
2779	C29	C31B	C32B	C23	-2(9)
2780	C29	C31B	C32B	C24	-176(13)
2781	C29	C31B	C32B	C28	168(10)
2782	C29	C31B	C32B	H32B	2
2783	C29	C31B	C32B	N33B	-178(12)
2784	C30B	C31B	C32B	C23	-7(2)
2785	C30B	C31B	C32B	C24	179(3)
2786	C30B	C31B	C32B	C28	163(4)
2787	C30B	C31B	C32B	H32B	-3
2788	C30B	C31B	C32B	N33B	177(2)
2789	C36B	C31B	C32B	C23	175(1)
2790	C36B	C31B	C32B	C24	1(5)
2791	C36B	C31B	C32B	C28	-15(2)
2792	C36B	C31B	C32B	H32B	179
2793	C36B	C31B	C32B	N33B	-1(3)
2794	C21	C31B	C36B	C27	-154(2)
2795	C21	C31B	C36B	H27	-50
2796	C21	C31B	C36B	C28	-125(6)
2797	C21	C31B	C36B	C35B	-175(1)
2798	C21	C31B	C36B	H36B	6
2799	C27	C31B	C36B	H27	104
2800	C27	C31B	C36B	C28	29(5)
2801	C27	C31B	C36B	C35B	-20(1)
2802	C27	C31B	C36B	H36B	160
2803	C29	C31B	C36B	C27	24(2)
2804	C29	C31B	C36B	H27	128
2805	C29	C31B	C36B	C28	53(5)
2806	C29	C31B	C36B	C35B	3(2)
2807	C29	C31B	C36B	H36B	-177
2808	C30B	C31B	C36B	C27	-155(2)
2809	C30B	C31B	C36B	H27	-51
2810	C30B	C31B	C36B	C28	-126(6)

2811	C30B	C31B	C36B	C35B	-175(1)
2812	C30B	C31B	C36B	H36B	5
2813	C32B	C31B	C36B	C27	23(3)
2814	C32B	C31B	C36B	H27	127
2815	C32B	C31B	C36B	C28	52(5)
2816	C32B	C31B	C36B	C35B	3(2)
2817	C32B	C31B	C36B	H36B	-177
2818	C23	C32B	H32B	C29	6
2819	C24	C32B	H32B	C23	171
2820	C24	C32B	H32B	C29	177
2821	C28	C32B	H32B	C23	-12
2822	C28	C32B	H32B	C29	-6
2823	C31B	C32B	H32B	C23	-7
2824	C31B	C32B	H32B	C29	-1
2825	N33B	C32B	H32B	C23	173
2826	N33B	C32B	H32B	C29	179
2827	C23	C32B	N33B	H24	5
2828	C23	C32B	N33B	C25	-177(3)
2829	C23	C32B	N33B	C29	-165(41)
2830	C23	C32B	N33B	C34B	-172(4)
2831	C24	C32B	N33B	H24	0
2832	C24	C32B	N33B	C25	178(4)
2833	C24	C32B	N33B	C29	-170(37)
2834	C24	C32B	N33B	C34B	-176(3)
2835	C28	C32B	N33B	H24	179
2836	C28	C32B	N33B	C25	-2(4)
2837	C28	C32B	N33B	C29	9(35)
2838	C28	C32B	N33B	C34B	3(3)
2839	C31B	C32B	N33B	H24	176
2840	C31B	C32B	N33B	C25	-6(5)
2841	C31B	C32B	N33B	C29	6(34)
2842	C31B	C32B	N33B	C34B	-0(3)
2843	H32B	C32B	N33B	H24	-4
2844	H32B	C32B	N33B	C25	174
2845	H32B	C32B	N33B	C29	-174
2846	H32B	C32B	N33B	C34B	180
2847	H24	N33B	C34B	C24	-150
2848	H24	N33B	C34B	C25	17
2849	H24	N33B	C34B	H25	11
2850	H24	N33B	C34B	C26	173
2851	H24	N33B	C34B	H34B	9
2852	H24	N33B	C34B	C35B	-171
2853	C25	N33B	C34B	C24	-168(17)
2854	C25	N33B	C34B	H25	-6
2855	C25	N33B	C34B	C26	156(3)

2856	C25	N33B	C34B	H34B	-8
2857	C25	N33B	C34B	C35B	172(2)
2858	C29	N33B	C34B	C24	20(16)
2859	C29	N33B	C34B	C25	-172(2)
2860	C29	N33B	C34B	H25	-178
2861	C29	N33B	C34B	C26	-16(3)
2862	C29	N33B	C34B	H34B	180
2863	C29	N33B	C34B	C35B	-0(2)
2864	C32B	N33B	C34B	C24	21(16)
2865	C32B	N33B	C34B	C25	-172(2)
2866	C32B	N33B	C34B	H25	-178
2867	C32B	N33B	C34B	C26	-16(3)
2868	C32B	N33B	C34B	H34B	-180
2869	C32B	N33B	C34B	C35B	0(2)
2870	C24	C34B	H34B	C25	6.5
2871	H25	C34B	H34B	C25	2
2872	C26	C34B	H34B	C25	-164
2873	N33B	C34B	H34B	C25	5.6
2874	C35B	C34B	H34B	C25	-174
2875	C24	C34B	C35B	C26	-150(2)
2876	C24	C34B	C35B	H26	-171
2877	C24	C34B	C35B	C27	-13(2)
2878	C24	C34B	C35B	H35B	-179
2879	C24	C34B	C35B	C36B	1(2)
2880	C25	C34B	C35B	C26	-130(7)
2881	C25	C34B	C35B	H26	-151
2882	C25	C34B	C35B	C27	7(7)
2883	C25	C34B	C35B	H35B	-159
2884	C25	C34B	C35B	C36B	21(7)
2885	H25	C34B	C35B	C26	25
2886	H25	C34B	C35B	H26	4
2887	H25	C34B	C35B	C27	162
2888	H25	C34B	C35B	H35B	-4
2889	H25	C34B	C35B	C36B	176
2890	C26	C34B	C35B	H26	-21
2891	C26	C34B	C35B	C27	137(3)
2892	C26	C34B	C35B	H35B	-29
2893	C26	C34B	C35B	C36B	151(2)
2894	N33B	C34B	C35B	C26	-149(2)
2895	N33B	C34B	C35B	H26	-170
2896	N33B	C34B	C35B	C27	-12(2)
2897	N33B	C34B	C35B	H35B	-178
2898	N33B	C34B	C35B	C36B	2(2)
2899	H34B	C34B	C35B	C26	31
2900	H34B	C34B	C35B	H26	10

2901	H34B	C34B	C35B	C27	168
2902	H34B	C34B	C35B	H35B	2
2903	H34B	C34B	C35B	C36B	-178
2904	H26	C35B	H35B	C26	2
2905	C27	C35B	H35B	C26	-155
2906	C34B	C35B	H35B	C26	11.9
2907	C36B	C35B	H35B	C26	-168
2908	C26	C35B	C36B	C27	-82(3)
2909	C26	C35B	C36B	H27	-150
2910	C26	C35B	C36B	C28	9(3)
2911	C26	C35B	C36B	C31B	15(3)
2912	C26	C35B	C36B	H36B	-165
2913	H26	C35B	C36B	C27	43
2914	H26	C35B	C36B	H27	-25
2915	H26	C35B	C36B	C28	134
2916	H26	C35B	C36B	C31B	140
2917	H26	C35B	C36B	H36B	-40
2918	C27	C35B	C36B	H27	-67
2919	C27	C35B	C36B	C28	91(3)
2920	C27	C35B	C36B	C31B	97(3)
2921	C27	C35B	C36B	H36B	-83
2922	C34B	C35B	C36B	C27	-100(3)
2923	C34B	C35B	C36B	H27	-168
2924	C34B	C35B	C36B	C28	-9(2)
2925	C34B	C35B	C36B	C31B	-3(2)
2926	C34B	C35B	C36B	H36B	177
2927	H35B	C35B	C36B	C27	80
2928	H35B	C35B	C36B	H27	12
2929	H35B	C35B	C36B	C28	171
2930	H35B	C35B	C36B	C31B	177
2931	H35B	C35B	C36B	H36B	-3
2932	H27	C36B	H36B	C27	0
2933	C28	C36B	H36B	C27	-154
2934	C31B	C36B	H36B	C27	-161
2935	C35B	C36B	H36B	C27	19

Deposition Number <url href="https://www.ccdc.cam.ac.uk/services/structures?id=doi:10.1002/open.202300219"> 2260655 (for **2b**)</url> contains the supplementary crystallographic data for this paper. These data are provided free of charge by the joint Cambridge Crystallographic Data Centre and Fachinformationszentrum Karlsruhe <url href="http://www.ccdc.cam.ac.uk/structures">Access Structures service</url>.
